# Molecular Architecture and Clinical Landscape of Immune Checkpoint Receptors and Ligands

**DOI:** 10.3390/antib15050084

**Published:** 2026-09-09

**Authors:** Milena Czosnek, Agata Sowa, Łucja Rolek, Ewelina Grywalska, Sebastian Mertowski, Paulina Mertowska

**Affiliations:** 1 Department of Experimental Immunology, Medical University of Lublin, 20-093 Lublin, Polandewelina.grywalska@umlub.edu.pl (E.G.); paulina.mertowska@umlub.edu.pl (P.M.); 2 Student Research Group of Experimental Immunology, Medical University of Lublin, 20-093 Lublin, Poland

**Keywords:** immune checkpoints, biomarkers, immune regulation, tumor microenvironment, immunotherapy, bioinformatics

## Abstract

Immune checkpoints (ICPs) are essential regulators of immune homeostasis, maintaining the balance between effective immune responses and tolerance to self-antigens. Dysregulation of ICP signaling may contribute to impaired immune surveillance, immune evasion, chronic inflammation, autoimmunity, persistent infections, and tumor progression. Consequently, ICP molecules are increasingly recognized not only as therapeutic targets but also as potential diagnostic, prognostic, predictive, and treatment-monitoring biomarkers. This review provides a comprehensive overview of the biological functions, signaling mechanisms, and clinical significance of major co-inhibitory and co-stimulatory ICP pathways, including PD-1/PD-L1/PD-L2, CTLA-4/CD28/CD80/CD86, LAG-3, TIM-3, TIGIT, BTLA, VISTA, ICOS, OX40, 4-1BB, GITR, CD27, CD40, and CD2, together with their corresponding ligands. Particular emphasis is placed on their biomarker potential in cancer and immune-mediated diseases. In addition, the review presents a bioinformatic characterization of ICP receptors and ligands based primarily on data available in UniProtKB and complementary bioinformatic resources. The analysis includes protein sequence length, molecular weight, theoretical isoelectric point, amino acid composition, subcellular localization, conserved and functional domains, protein family classification, post-translational modifications, isoforms, and selected structural features. Collectively, the available evidence indicates that ICPs constitute a structurally and functionally diverse group of immunoregulatory molecules with substantial biomarker potential. Integrating their molecular, structural, functional, and bioinformatic characteristics may improve disease classification, prognosis, patient stratification, treatment selection, and therapeutic monitoring. Such an integrated approach may also support the identification of novel biomarkers and therapeutic targets and contribute to the further development of precision and personalized medicine.

## 1. Introduction

The human immune system is a highly organized and precisely regulated biological system whose primary function is to recognize and eliminate pathogens, infected cells, and cancer cells while maintaining tolerance to self-antigens. An effective immune response requires maintaining a dynamic balance between signals that activate immune cells and mechanisms that limit excessive, inappropriate, or chronic activation. Disturbance of this balance can lead to chronic inflammation, tissue damage, and autoimmune diseases, as well as impaired immune surveillance, thereby allowing cancer cells to evade the immune response and further disease progression [1,2,3,4,5,6,7,8]. Immune checkpoints (ICPs) play a key role in maintaining immune homeostasis. They constitute a complex network of membrane receptors, their corresponding ligands, and the associated intracellular signaling pathways. These molecules regulate the activation, proliferation, differentiation, survival, and effector functions of immune cells, influencing the intensity, duration, and nature of the immune response. Depending on the signal they transmit, immune checkpoints can exhibit co-inhibitory effects, limiting immune cell activity, or costimulatory effects, enhancing their activation and effector functions [9,10,11,12,13,14].

ICPs do not function as single, independent elements, but as a dynamic system of interconnected interactions between T lymphocytes, B lymphocytes, NK cells, antigen-presenting cells (APCs), myeloid cells, and non-immune cells. Their interactions with tumor cells and other components of the tissue microenvironment are particularly important. Coordinated receptor–ligand interactions enable precise adjustment of the immune response to the type and duration of the stimulus, ensuring effective elimination of threats while limiting the risk of autoimmunity and excessive tissue damage [9,10,11,12,13,14]. Under physiological conditions, co-inhibitory pathways are responsible for, among other things, quenching the immune response following antigen elimination, maintaining peripheral tolerance, and preventing uncontrolled lymphocyte activation. Co-stimulatory pathways, in turn, provide additional signals necessary for full activation, clonal expansion, differentiation, and survival of immune cells. A balance between inhibitory and activating signals is therefore essential for proper immune system function. Disruption of this balance can lead to effector cell exhaustion, excessive immunosuppression, loss of immune tolerance, or uncontrolled perpetuation of the inflammatory response [1,2,3,4,5,6,7,8].

In recent years, immune checkpoints have become one of the most intensively developing areas of research in immunology, oncology, and translational medicine. Initial interest in these molecules stemmed primarily from their role in regulating T cell activation and their potential as targets for anticancer immunotherapy by exploiting inhibitory pathways. Antibodies blocking the programmed cell death protein 1/programmed death-ligand 1 (PD-1/PD-L1) and cytotoxic T-lymphocyte-associated protein 4 (CTLA-4) pathways are of particular clinical importance, enabling partial restoration of the antitumor activity of immune cells. However, the biological and clinical significance of immune checkpoints extends far beyond the regulation of antitumor responses. Alterations in the expression, localization, ligand availability, and activity of immune checkpoint pathways are observed in many cancers, autoimmune diseases, chronic inflammatory and infectious diseases, and conditions associated with immunodeficiency, immunosenescence, and chronic antigenic stimulation. Depending on the biological context, they may reflect activation of the immune system, development of tolerance, functional exhaustion of lymphocytes, immunosuppression or adaptive mechanisms of immune evasion by diseased cells [1,2,3,4,5,6,7,8,9,10,11,12,13,14].

A growing body of research also indicates that immune checkpoint receptors and ligands may have potential biomarker relevance. Their expression on cell surfaces, the concentration of soluble forms in biological fluids, the presence of specific isoforms, transcriptional changes, post-translational modifications, and structural properties have been investigated in relation to disease presence, activity and progression, prognosis, treatment response, and therapeutic efficacy. At the same time, heterogeneity in checkpoint expression, dependence on cell type, microenvironment, and disease stage, and the existence of mutually compensating signaling pathways complicate the use of single molecules as universal biomarkers. Therefore, multiparametric profiles, which involve the simultaneous analysis of multiple receptors, ligands, and their associated molecular mechanisms, are becoming increasingly important. A comprehensive understanding of the importance of immune checkpoints requires not only analysis of their biological and clinical functions but also consideration of their molecular and structural properties. Information on protein sequence length, molecular weight, isoelectric point, amino acid composition, subcellular localization, domain organization, protein family affiliation, post-translational modifications, isoforms, and spatial structural elements can facilitate interpretation of their function, stability, ligand interactions, and regulatory susceptibility. Bioinformatic characterization can also support the identification of potential binding sites, functional regions, clinically significant variants, and novel diagnostic and therapeutic targets.

This review aims to comprehensively systematize current knowledge of the most important co-inhibitory and co-stimulatory immune checkpoint receptors and their corresponding ligands. This review discusses their biological functions, signaling mechanisms, and clinical relevance, with particular emphasis on their potential diagnostic, prognostic, and predictive roles in cancer and immune-mediated diseases. An additional goal of this work is to systematically characterize selected immune checkpoint receptors and ligands using data from the UniProtKB database [15] and complementary bioinformatics resources and tools. The analysis includes length and basic properties of protein sequences, molecular weight, theoretical isoelectric point, amino acid composition, subcellular localization, functional domains, protein family classification, post-translational modifications, isoforms, and selected structural features. This integrated approach allows us to present immune checkpoints as key regulators of homeostasis and the immune response, as well as promising biomarkers and targets for translational research that supports the development of precision and personalized medicine.

## 2. Materials and Methods

### 2.1. Selection of Immune Checkpoint Pathways and Analyzed Molecules

The scope of the analysis included selected immune checkpoint receptors and ligands involved in co-inhibitory and costimulatory regulation of the immune response. The co-inhibitory receptors included B- and T-lymphocyte attenuator (BTLA), CTLA-4 (CD152), PD-1 (CD279), T-cell immunoreceptor with Ig and ITIM domains (TIGIT), lymphocyte activation gene 3 (LAG-3/CD223), T-cell immunoglobulin and mucin-domain containing-3/hepatitis A virus cellular receptor 2 (TIM-3/HAVCR2), V-domain Ig suppressor of T-cell activation/V-set immunoregulatory receptor (VISTA/VSIR), CD112R/poliovirus receptor-related immunoglobulin domain-containing protein (PVRIG), carcinoembryonic antigen-related cell adhesion molecule 1 (CEACAM1), CD96, CD160, and P-selectin glycoprotein ligand-1/selectin P ligand gene (PSGL-1/SELPLG). The analysis also included ligands involved in inhibitory signaling, including PD-L1 (CD274), programmed death-ligand 2 (PD-L2/CD273), galectin-9, fibrinogen-like protein 1 (FGL1), CD155/poliovirus receptor (PVR), CD112/nectin cell adhesion molecule 2 (NECTIN2), CEACAM1, VISTA, B7-H3 (CD276), and B7-H4 (VTCN1). Co-stimulatory molecules included CD28, inducible T-cell costimulator (ICOS/CD278), OX40 receptor/tumor necrosis factor superfamily 4 (CD134/TNFRSF4), 4-1BB receptor (CD137/TNFRSF9), glucocorticoid-induced TNFR-related protein (GITR/TNFRSF18), CD27, CD40, CD226/DNAX accessory molecule-1 (DNAM-1), CD2, and herpesvirus entry mediator (HVEM/TNFRSF14), as well as their corresponding ligands: CD80 (B7-1), CD86 (B7-2), inducible T-cell costimulator ligand (ICOSL), OX40 ligand (OX40L), 4-1BB ligand (4-1BBL), glucocorticoid-induced TNFR-related protein ligand (GITRL), CD70, CD40L (CD154), CD58 (LFA-3), and homologous to lymphotoxins, exhibits inducible expression (LIGHT/TNFSF14). The selection of molecules was based on their documented roles in regulating the activation, proliferation, differentiation, survival, and effector functions of immune cells. The selection included well-characterized immune checkpoint pathways with established or context-dependent clinical relevance, including pathways targeted in immunotherapy, as well as emerging receptors and ligands for which biological, biomarker, or therapeutic relevance is currently being investigated.

### 2.2. Molecular and Physicochemical Characterization of Analyzed Proteins

Molecular characterization of the analyzed proteins was performed primarily using data from the publicly available UniProt Knowledgebase (UniProtKB) [15]. For each molecule, information was obtained regarding the official protein name, gene symbol, amino acid sequence length, predicted or experimentally determined molecular weight, theoretical isoelectric point (pI), subcellular localization, isoform occurrence, protein family affiliation, domain organization, the presence of a signal peptide and transmembrane segments, post-translational modifications, and processes related to protein maturation and proteolytic processing. These parameters were selected to provide complementary information on protein identity, sequence-derived physicochemical properties, structural organization, cellular localization, and molecular processing. Comparative physicochemical characterization was performed using the canonical protein sequences obtained from UniProtKB. Protein length, molecular weight, relative proportions of hydrophobic and hydrophilic amino acid residues, and theoretical pI were evaluated. The proportions of hydrophobic and hydrophilic residues were calculated as percentages of the total number of amino acid residues in each canonical sequence. The theoretical pI was calculated from the canonical amino acid sequence using the Isoelectric Point Calculator (IPC), based on optimized pKa datasets for protein pI prediction [16].

For proteins with multiple described isoforms, the canonical UniProtKB sequence was used as the standardized reference for comparative physicochemical analyses, while information on alternative isoforms was retained for descriptive characterization. Structural and functional annotations obtained from UniProtKB were used to provide additional information on protein organization, localization, maturation, and molecular processing. The selected parameters were used to establish a standardized molecular profile for comparative characterization of the analyzed immune checkpoint proteins.

### 2.3. Molecular and Physicochemical Characterization of Analyzed Proteins

Figures included in this review were prepared using BioRender.com, (August 2026), including AI-assisted features available within the BioRender platform to support the arrangement and composition of graphical elements. These tools were used solely for figure preparation and did not contribute to the generation, analysis, or interpretation of scientific data or to the formulation of scientific conclusions. All graphical content was reviewed and verified by the authors, who take full responsibility for the accuracy of the final figures.

## 3. Mechanisms of Action and Classification of ICPs

**Figure 1:**
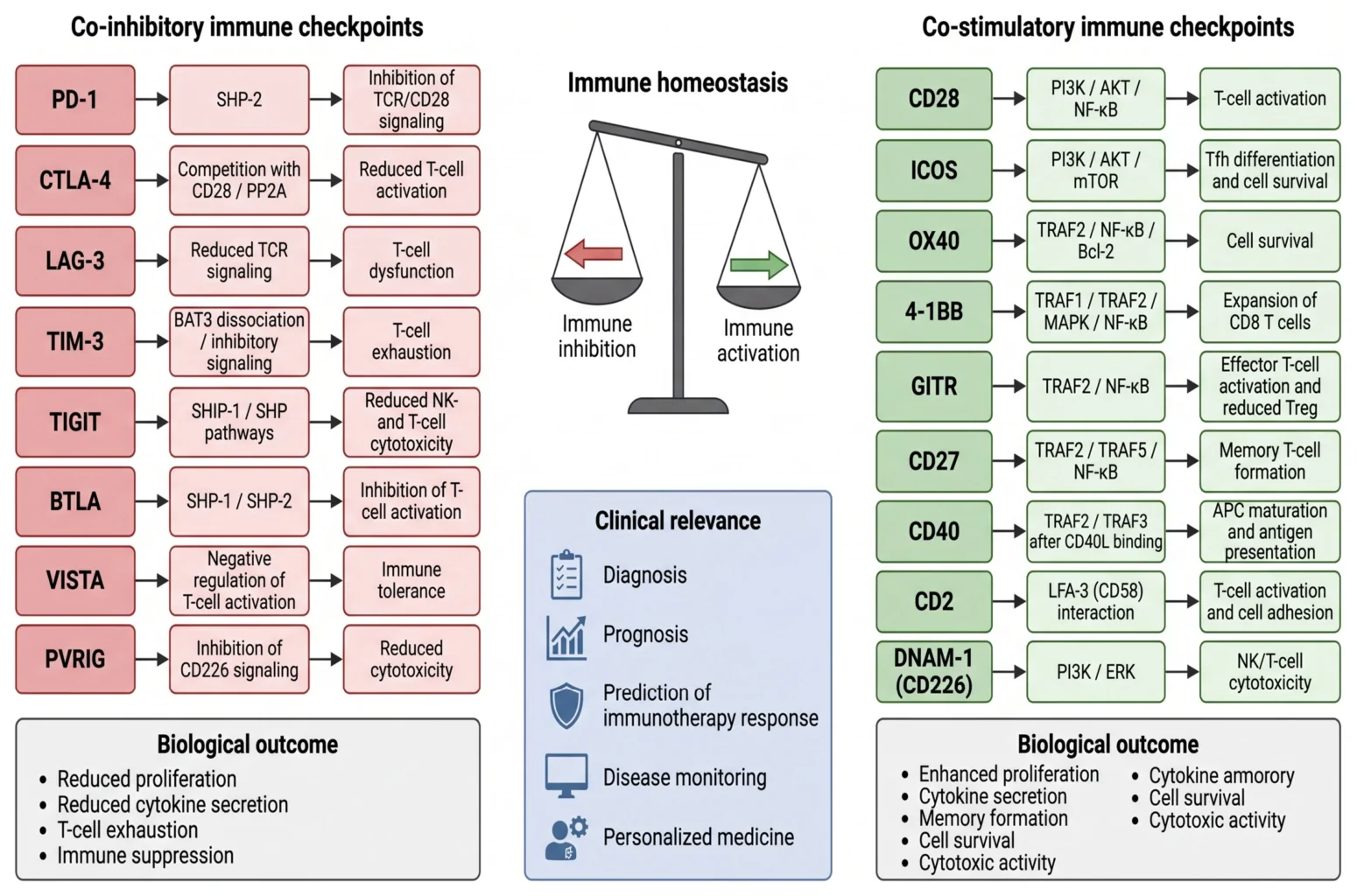
Functional balance between co-inhibitory and co-stimulatory immune checkpoints [17,18,19,20,21]. Arrows indicate the direction of signaling/functional relationships from immune checkpoint receptors through downstream signaling pathways to their functional outcomes. Abbreviations: AKT, protein kinase B; APC, antigen-presenting cell; BAT3, HLA-B-associated transcript 3; Bcl-2, B-cell lymphoma 2; BTLA, B- and T-lymphocyte attenuator; CD, cluster of differentiation; CTLA-4, cytotoxic T-lymphocyte-associated protein 4; DNAM-1, DNAX accessory molecule 1; ERK, extracellular signal-regulated kinase; GITR, glucocorticoid-induced TNFR-related protein; ICOS, inducible T-cell co-stimulator; LAG-3, lymphocyte activation gene 3; LFA-3, lymphocyte function-associated antigen 3; MAPK, mitogen-activated protein kinase; mTOR, mechanistic target of rapamycin; NF-κB, nuclear factor kappa B; NK, natural killer; OX40, tumor necrosis factor receptor superfamily member 4; PD-1, programmed cell death protein 1; PI3K, phosphoinositide 3-kinase; PP2A, protein phosphatase 2A; PVRIG, poliovirus receptor-related immunoglobulin domain-containing protein; SHIP-1, SH2 domain-containing inositol 5-phosphatase 1; SHP-1/2, Src homology 2 domain-containing protein tyrosine phosphatase 1/2; TCR, T-cell receptor; Tfh, follicular helper T cell; TIGIT, T-cell immunoreceptor with immunoglobulin and ITIM domains; TIM-3, T-cell immunoglobulin and mucin-domain containing-3; TRAF, TNF receptor-associated factor; Treg, regulatory T cell; VISTA, V-domain immunoglobulin suppressor of T-cell activation, 4-1BB/CD137 tumor necrosis factor receptor superfamily member 9; TNFRSF9. Created in BioRender. Mertowski, S. (2026) https://BioRender.com/5zo4a5t. However, inadequate costimulation with retained antigen recognition can lead to anergy, a state in which the lymphocyte is functionally unable to respond to restimulation. This phenomenon is one of the fundamental mechanisms that maintain peripheral tolerance and prevent the undesirable activation of autoreactive cells [17,18,19,20,21]. Activating and costimulatory signals are counterbalanced by signals transmitted by coinhibitory receptors. After binding appropriate ligands, these receptors limit lymphocyte activation by attenuating TCR-dependent signaling and costimulatory receptors. Some inhibitory receptors contain ITIM (immunoreceptor tyrosine-based inhibitory motif) and ITSM (immunoreceptor tyrosine-based switch motif) motifs in their cytoplasmic domains, which enable the recruitment of phosphatases such as src homology 2 domain-containing protein tyrosine phosphatase 1 (SHP-) and src homology 2 domain-containing protein tyrosine phosphatase 2 (SHP-2). These enzymes dephosphorylate key elements of signaling pathways, leading to inhibition of PI3K/AKT activation, reduced proliferation, reduced cytokine production, and impaired cytotoxic effector cell function. However, the mechanisms of action of individual coinhibitory receptors are not uniform, and not all utilize classic ITIM or ITSM motifs [17,18,19,20,21,22]. The ultimate effect of the immune response therefore depends on the relative predominance of activating, costimulatory, and inhibitory signals. This balance enables effective elimination of pathogens, infected cells, and cancer cells while preventing excessive immune activation, tissue damage, and the development of autoimmunity [15,16,17,18,19,20]. This balance is influenced by receptor and ligand expression levels, the cell types involved, their activation stage, and the conditions in the tissue microenvironment. Disturbance of the normal balance between activating and inhibitory signals leads to dysregulation of the immune response. A predominance of costimulatory signals can promote chronic activation of immune cells, perpetuate inflammation, and lead to the development of autoimmune diseases. In turn, excessive activation of co-inhibitory pathways leads to immunosuppression, impaired effector function, and impaired immune surveillance. These mechanisms are important in the pathogenesis of cancer, chronic infections, inflammatory and autoimmune diseases, and justify the use of immune checkpoints as biomarkers and therapeutic targets [20,21,22,23].

T cell activation is a multistage process dependent on the integration of activating, costimulatory, and inhibitory signals. The first signal is generated when the T-cell receptor (TCR) recognizes a specific antigen presented in a complex with major histocompatibility complex (MHC) molecules on the surface of APCs. Recognition of the antigen–MHC complex determines the specificity of the response, but it is not sufficient in itself for full T cell activation [17,18,19]. An additional costimulatory signal is necessary to develop an effective effector response. A classic example is the interaction between the CD28 receptor on T lymphocytes and the CD80 and CD86 ligands on the surface of APCs. Simultaneous activation of TCR and costimulatory receptors activates intracellular pathways, including phosphoinositide 3-kinase/protein kinase B signaling pathway/mechanistic target of rapamycin (PI3K/AKT/mTOR), nuclear factor kappa B (NF-κB), and mitogen-activated protein kinases (MAPKs). For receptors in the tumor necrosis factor receptor superfamily, adaptor proteins from the TRAF family also play an important role in signal transduction. Activation of these mechanisms promotes T cell proliferation, survival, differentiation, metabolic activity, and effector functions [17,18,19,20] (Figure 1).

Based on the direction of signal transduction and its biological consequences, immune checkpoints are divided into costimulatory and co-inhibitory checkpoints. Co-stimulatory checkpoints enhance the activation and effector function of immune cells, whereas co-inhibitory checkpoints limit the intensity and duration of the response. Both groups form an interconnected regulatory system whose proper functioning is essential for maintaining immune homeostasis [17,18,19,20,21,22,23] (Figure 1).

### 3.1. Co-Stimulatory ICPs

Co-stimulatory ICPs amplify signals initiated by antigen recognition, enabling full activation, clonal expansion, and differentiation of immune cells. Their action extends not only to T lymphocytes but also to B lymphocytes, NK cells, and antigen-presenting cells. Receptors belonging to the immunoglobulin superfamily, such as CD28 and ICOS, primarily activate the PI3K/AKT and mTOR pathways, while receptors from the TNFR superfamily, including OX40, 4-1BB, GITR, CD27, CD40, and HVEM, transmit signals primarily through TRAF adaptor proteins, leading to the activation of NF-κB and MAPK. Activation of these pathways promotes immune cell survival and proliferation, cytokine production, enhanced cytotoxicity, and the development of memory cells [23,24,25]. However, their excessive or prolonged stimulation may sustain chronic inflammation and promote loss of immune tolerance. Due to their ability to enhance antitumor responses, costimulatory molecules are being evaluated as targets for agonist and combination therapies, and their expression may have prognostic and predictive significance [24,25,26,27,28]. Characteristics of selected costimulatory receptors and their ligands are presented in Table 1 and Table 2.

### 3.2. Co-Inhibitory ICPs

Co-inhibitory ICPs limit the intensity and duration of the immune response, supporting the quenching of activation after antigen elimination, maintaining peripheral tolerance, and protecting tissues from immune damage [1,13,17]. Their expression often transiently increases following lymphocyte activation as part of a physiological negative feedback loop. Under conditions of chronic antigen stimulation, sustained expression of multiple inhibitory receptors can promote T cell exhaustion, characterized by a gradual loss of effector function and by metabolic, transcriptional, and epigenetic changes. In the tumor microenvironment, these pathways are often utilized to suppress the antitumor response by limiting the proliferation, cytokine production, and cytotoxic activity of effector cells. Blocking co-inhibitory receptors or their ligands has enabled the development of immune checkpoint inhibitors, including therapies targeting CTLA-4 and PD-1/PD-L1 [21,24,25,26,49]. At the same time, membrane expression, the presence of soluble forms, and the coexistence of multiple inhibitory pathways may have diagnostic, prognostic, and predictive significance. Characteristics of selected coinhibitory receptors and their ligands are presented in Table 3 and Table 4.

## 4. Molecular Characterization of Immune Checkpoints and Pathways Regulating the Immune Response

### 4.1. The PD-1/PD-L1/PD-L2 Axis

The PD-1/PD-L1/PD-L2 axis is one of the best-characterized co-inhibitory mechanisms regulating the immune response. Its physiological function is to limit excessive activation of effector cells, especially T lymphocytes, thereby suppressing the response after antigen elimination, maintaining peripheral tolerance, and protecting tissues from immune damage. However, under chronic antigen stimulation, particularly in the tumor microenvironment, this pathway can permanently impair effector functions and facilitate tumor cell evasion of immune surveillance [70,71,72,73]. PD-1 (CD279) is a type I transmembrane protein belonging to the CD28 receptor family, encoded by the *PDCD1* gene located on chromosome 2q37.3. Its expression is induced primarily following antigenic activation and involves primarily CD4^+^ and CD8^+^ T lymphocytes, but also B lymphocytes, NK cells, and selected dendritic cell populations. Transient increases in PD-1 expression constitute part of a physiological negative feedback loop, whereas its persistence under long-term antigen exposure is characteristic of T cell exhaustion, accompanied by a gradual loss of proliferative capacity, cytokine secretion, and cytotoxic activity [71,72,73,74,75].

**Figure 2:**
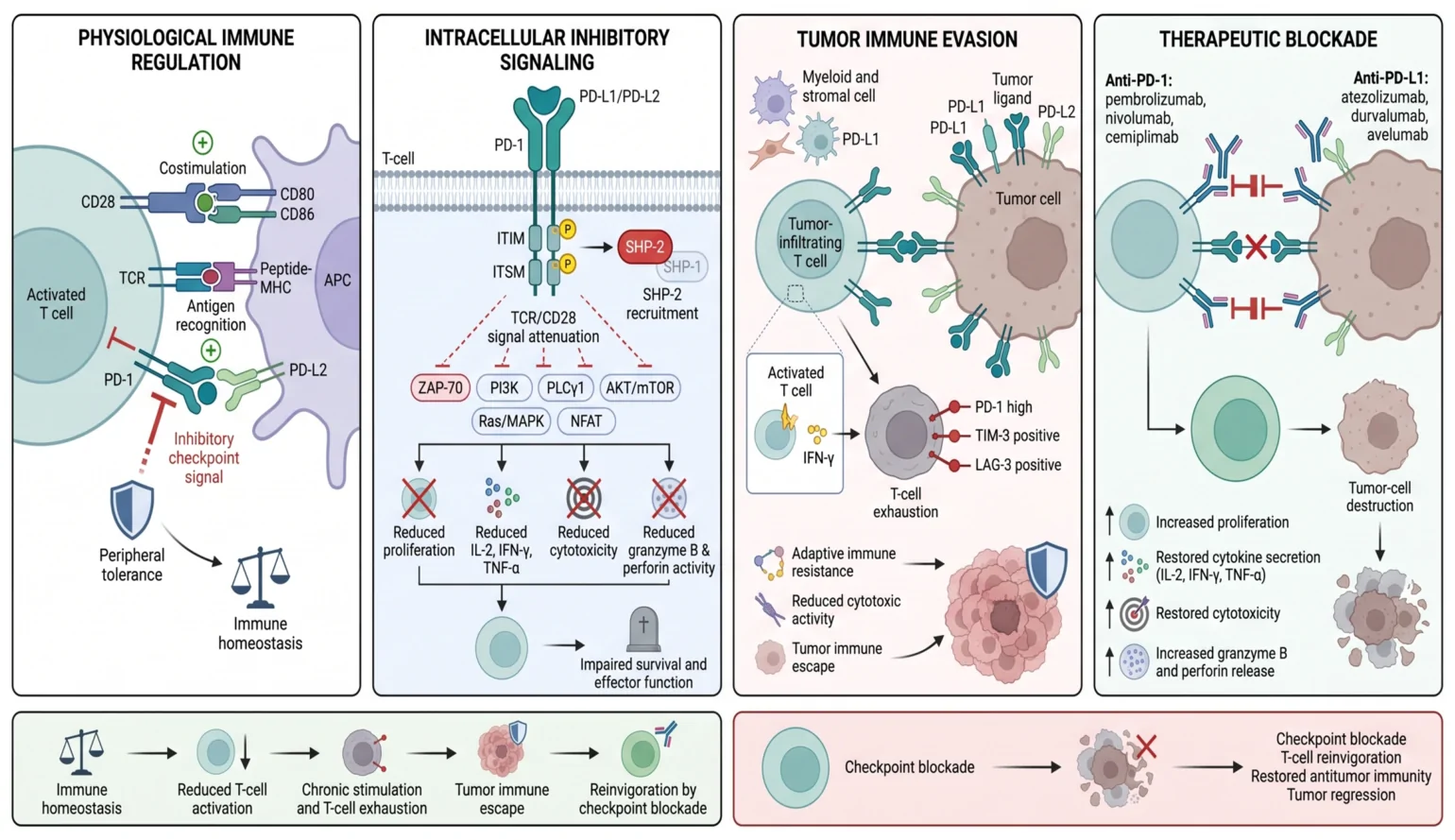
Structural organization and functional annotation of the PD-1/PD-L1/PD-L2 immune checkpoint axis [52,64,65]. Solid arrows indicate the direction of signaling, functional relationships, or biological progression. Dashed red lines indicate inhibitory or attenuating effects on downstream signaling. Green plus symbols indicate stimulatory/activating effects, red inhibitory symbols indicate inhibition or blockade, and red crosses indicate disruption of immune-checkpoint interactions by therapeutic antibodies. Abbreviations:AKT, protein kinase B; APC, antigen-presenting cell; CD28, cluster of differentiation 28; CD80, cluster of differentiation 80; CD86, cluster of differentiation 86; IFN-γ, interferon gamma; IL-2, interleukin-2; ITIM, immunoreceptor tyrosine-based inhibitory motif; ITSM, immunoreceptor tyrosine-based switch motif; LAG-3, lymphocyte activation gene 3; MAPK, mitogen-activated protein kinase; MHC, major histocompatibility complex; mTOR, mechanistic target of rapamycin; NFAT, nuclear factor of activated T cells; PD-1, programmed cell death protein 1; PD-L1, programmed death-ligand 1; PD-L2, programmed death-ligand 2; PI3K, phosphoinositide 3-kinase; PLCγ1, phospholipase C gamma 1; Ras, rat sarcoma GTPase; SHP-1, Src homology 2 domain-containing phosphatase 1; SHP-2, Src homology 2 domain-containing phosphatase 2; TCR, T-cell receptor; TIM-3, T-cell immunoglobulin and mucin-domain containing-3; TNF-α, tumor necrosis factor alpha; ZAP-70, zeta-chain-associated protein kinase 70. Created in BioRender. Mertowski, S. (2026) https://BioRender.com/5zo4a5t.

The PD-1 ligands are PD-L1 (CD274, B7-H1) and PD-L2 (CD273, B7-DC), members of the B7 family. PD-L1 has a broad expression profile and is found on both immune and non-hematopoietic cells, including endothelial, epithelial, and tumor cells. PD-L2 exhibits a more restricted expression, primarily on activated dendritic cells, macrophages, and selected B cell populations. Despite its narrower expression spectrum, PD-L2 exhibits higher affinity for PD-1 and can effectively modulate T cell responses at sites of active immune response [72,73,74]. The biological properties of the axis components are closely related to their structural organization. PD-1 contains an extracellular immunoglobulin-like V domain, a single transmembrane segment, and a cytoplasmic domain with ITIM and ITSM motifs that mediate inhibitory signaling. The extracellular IgV domain forms the principal ligand-binding interface, while the ITIM and ITSM motifs are essential for the recruitment of intracellular signaling molecules and initiation of inhibitory signaling [72,73,74]. PD-L1 and PD-L2 are type I transmembrane glycoproteins containing IgV and IgC2 domains that mediate receptor interactions. The transmembrane segments anchor PD-L1 and PD-L2 in the plasma membrane, allowing their extracellular domains to interact with PD-1 on neighboring cells. The stability, localization, and activity of these proteins are further modulated by posttranslational modifications, including N-linked glycosylation, phosphorylation, ubiquitination, and disulfide bond formation, as well as by the presence of isoforms and sequence variants [72,73,74]. In particular, N-linked glycosylation of PD-L1 contributes to protein stability and may affect the accessibility of epitopes recognized by anti-PD-L1 antibodies. Experimental evidence indicates that the glycosylation state of PD-L1 can therefore influence its detection by immunohistochemistry, linking a structural feature of the molecule with the analytical measurement of this biomarker [73,74]. An integrated characterization of the membrane topology, domain organization, posttranslational modification sites, and selected functional annotations of PD-1, PD-L1, and PD-L2 is presented in Figure 2 [52,64,65].

**Figure 3:**
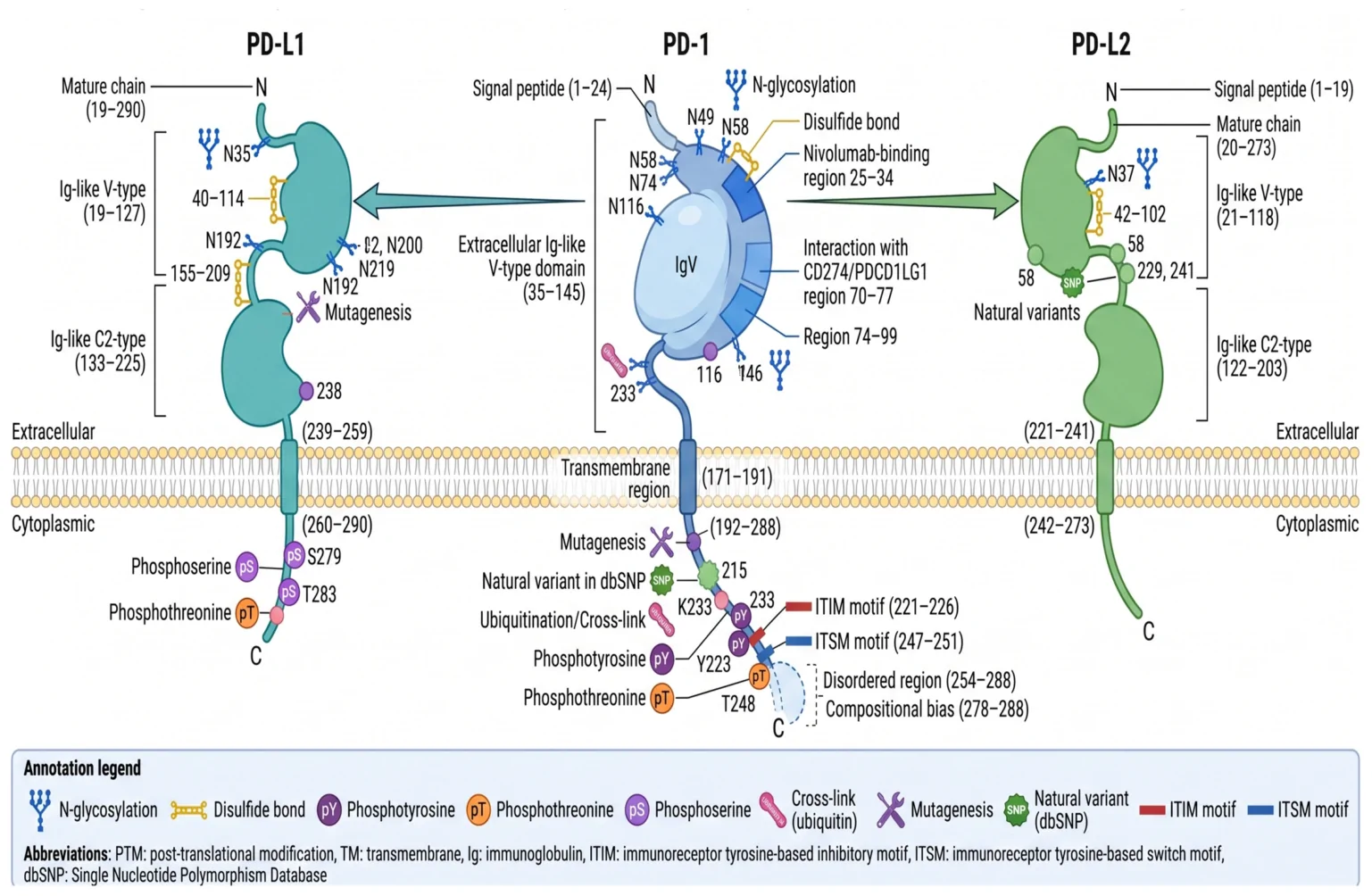
Mechanism of action and therapeutic modulation of the PD-1/PD-L1/PD-L2 immune checkpoint axis [70,71,72,73,74,75,76,77,78]. Abbreviations: CD274, cluster of differentiation 274; dbSNP, Single Nucleotide Polymorphism Database; Ig, immunoglobulin; IgC2, immunoglobulin-like C2-type domain; IgV, immunoglobulin-like V-type domain; ITIM, immunoreceptor tyrosine-based inhibitory motif; ITSM, immunoreceptor tyrosine-based switch motif; PD-1, programmed cell death protein 1; PD-L1, programmed death-ligand 1; PD-L2, programmed death-ligand 2; PDCD1LG1, programmed cell death 1 ligand 1; PTM, post-translational modification; pS, phosphoserine; pT, phosphothreonine; pY, phosphotyrosine; SNP, single-nucleotide polymorphism; TM, transmembrane; N, N-terminus; C, C-terminus. Created in BioRender. Mertowski, S. (2026) https://BioRender.com/5zo4a5t.

Binding of PD-1 to PD-L1 or PD-L2 leads to phosphorylation of ITIM and ITSM motifs in the cytoplasmic domain of the receptor and to the recruitment of tyrosine phosphatases, primarily SHP-2 and, to a lesser extent, SHP-1. These phosphatases attenuate TCR- and CD28-dependent signaling by dephosphorylating key signaling molecules, including ZAP-70, PI3K, and PLCγ1 [73,74,75,76]. This results in reduced T cell proliferation, reduced production of IL-2, IFN-γ, and TNF-α, reduced cytotoxicity, and impaired cell survival and effector function. Short-term activation of the axis therefore plays a homeostatic role, whereas chronic stimulation promotes T cell exhaustion. The mechanism of action of the PD-1/PD-L1/PD-L2 axis, including physiological regulation of the response, intracellular inhibitory pathways, role in tumor escape from immune surveillance, and the effects of therapeutic blockade, is presented in Figure 3 [70,71,72,73,74,75,76,77,78,79].

In the tumor microenvironment, PD-L1 expression can be increased both due to the activation of oncogenic pathways and in response to interferon γ secreted by activated T lymphocytes. This phenomenon, referred to as adaptive immune resistance, leads to increased PD-1 signaling, inhibition of tumor-infiltrating lymphocytes’ activity, and limited elimination of tumor cells. Consequently, the PD-1/PD-L1/PD-L2 axis combines the function of a physiological regulator of homeostasis with the immunosuppressive mechanism used by tumors, which justifies its crucial biological, biomarker, and therapeutic importance. From a biomarker perspective, these structural characteristics are particularly relevant for PD-L1, as protein stability, posttranslational modification, and epitope accessibility may influence the level detected by immunohistochemical assays [76,77,78,79].

### 4.2. CTLA-4/CD28-CD80/CD86

The CTLA-4/CD28–CD80/CD86 axis is a prototypical regulatory system integrating costimulatory and coinhibitory signals during T cell activation. CD28 and CTLA-4 receptors compete for common ligands, CD80 (B7-1) and CD86 (B7-2), present primarily on the surface of professional antigen-presenting cells. The balance between CD28-dependent signaling and the inhibitory effects of CTLA-4 determines the threshold for T cell activation, the intensity of the effector response, and the maintenance of peripheral tolerance. CD28 is a transmembrane glycoprotein in the immunoglobulin superfamily and is the primary costimulatory receptor for T lymphocytes. It is constitutively expressed on most CD4^+^ T cells and on a significant proportion of CD8^+^ T cells [80,81,82]. Simultaneous recognition of the peptide–MHC complex by the TCR and binding of CD28 to CD80 or CD86 lead to activation of the PI3K/AKT, NF-κB, NFAT, and AP-1 pathways [14,79,80,81]. This results in enhanced IL-2 synthesis, proliferation, survival, metabolic reprogramming, and differentiation of effector cells. A lack of appropriate costimulation can lead to anergy or the initiation of other mechanisms of peripheral tolerance. CD28 also participates in the development, homeostasis, and maintenance of regulatory T cell function, so disruption of this pathway can lead to both attenuated antitumor responses and inappropriate autoimmune activation [79,80]. CTLA-4 is a coinhibitory receptor belonging to the same protein superfamily. It is encoded by the CTLA4 gene, located on chromosome 2q33. Unlike CD28, CTLA-4 expression on naive T cells is low, but increases rapidly following TCR activation. This receptor is also constitutively expressed on regulatory T cells, where it plays a key role in maintaining immune tolerance and limiting excessive immune activation [10,80,81,82].

CTLA-4 binds CD80 and CD86 with greater affinity than CD28, effectively limiting ligand availability to the costimulatory receptor. This leads to impaired T cell activation, reduced proliferation, and reduced IL-2 production. Additionally, the cytoplasmic domain of CTLA-4 recruits molecules that inhibit TCR- and CD28-dependent signaling. Transendocytosis of CD80 and CD86 from the surface of antigen-presenting cells is also a key regulatory element, leading to their internalization and degradation, and a persistent reduction in the costimulatory potential of APCs. This mechanism is particularly important for the immunosuppressive function of regulatory T cells [79,80,82]. CD80 and CD86 ligands belong to the B7 family, but differ in their expression dynamics and involvement in specific stages of the immune response. CD86 can be constitutively present on some APCs and is rapidly induced upon activation, playing a crucial role in the early phase of T cell response initiation. CD80 expression typically increases later, and its relatively stronger interaction with CTLA-4 promotes response limitation and restoration of immune homeostasis [81,82,83].

**Figure 4:**
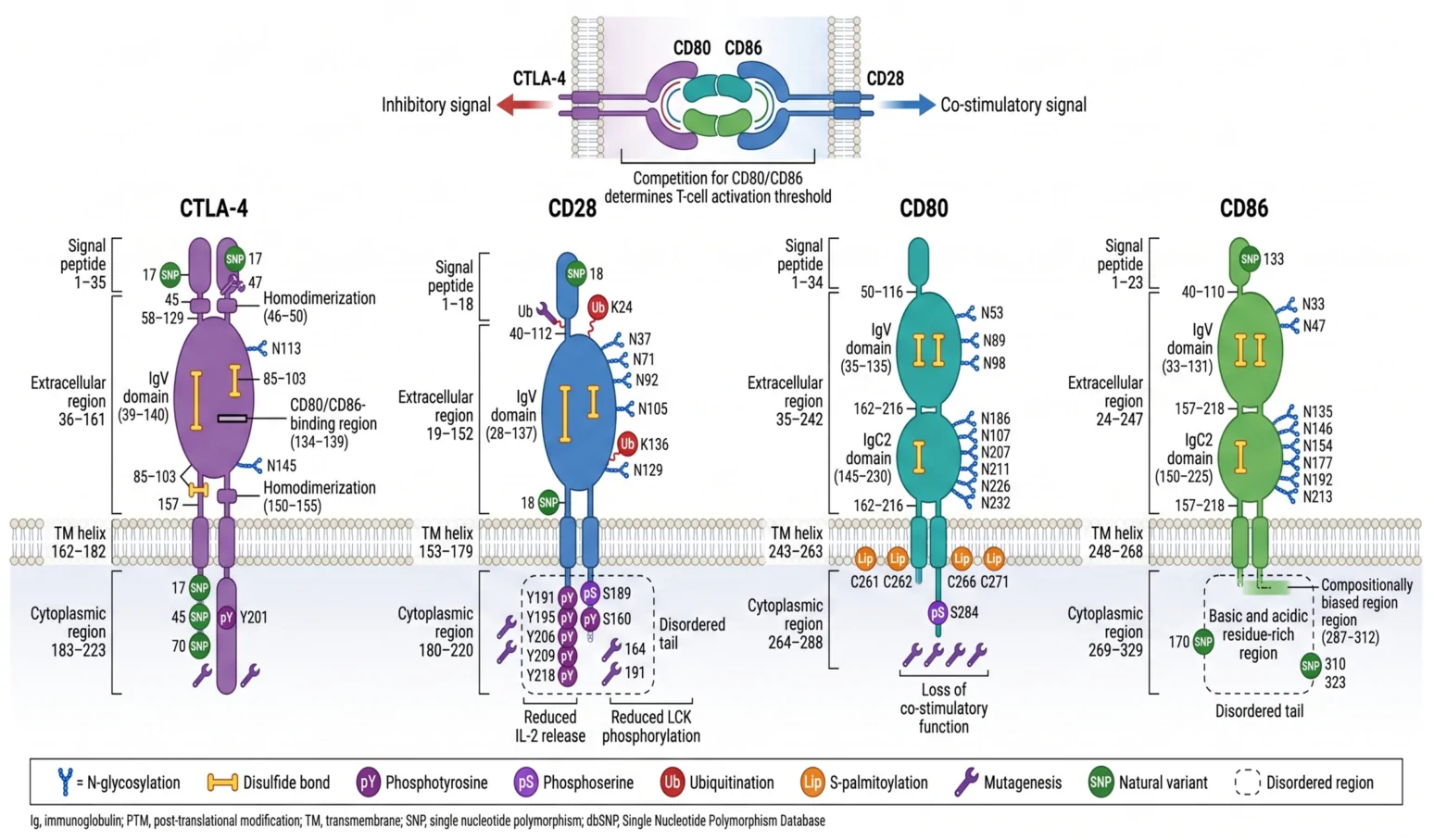
Structural and functional organization of the CTLA-4/CD28–CD80/CD86 immune checkpoint axis [31,39,40,51]. The upper schematic illustrates the competition between CTLA-4 and CD28 for binding to the co-stimulatory ligands CD80 and CD86, which contributes to determining the T-cell activation threshold. The central green/teal structures represent CD80 and CD86 ligands interacting with CTLA-4 and CD28. The red arrow indicates inhibitory signaling mediated by CTLA-4, whereas the blue arrow indicates co-stimulatory signaling mediated by CD28. Abbreviations: CD28, cluster of differentiation 28; CD80, cluster of differentiation 80; CD86, cluster of differentiation 86; CTLA-4, cytotoxic T-lymphocyte-associated protein 4; dbSNP, Single Nucleotide Polymorphism Database; Ig, immunoglobulin; IgC2, immunoglobulin-like C2-type domain; IgV, immunoglobulin-like V-type domain; IL-2, interleukin-2; LCK, lymphocyte-specific protein tyrosine kinase; Lp, S-palmitoylation; PTM, post-translational modification; pS, phosphoserine; pY, phosphotyrosine; SNP, single-nucleotide polymorphism; TM, transmembrane; Ub, ubiquitination. Created in BioRender. Mertowski, S. (2026) https://BioRender.com/5zo4a5t.

The structural organization of the components of this axis determines their functional properties. CTLA-4 and CD28 are type I transmembrane proteins containing an extracellular IgV domain, a single transmembrane segment, and a cytoplasmic domain. The extracellular IgV domains of CTLA-4 and CD28 form the principal interfaces for binding CD80 and CD86, while differences in the organization and binding properties of these receptors contribute to their opposing functional effects despite recognizing the same ligands. CD80 and CD86, on the other hand, contain extracellular IgV and IgC2 domains that mediate receptor interactions. Their extracellular domains determine the ligand-binding interface, whereas the transmembrane regions maintain CD80 and CD86 at the cell surface, enabling interaction with CD28 and CTLA-4 on T cells [82,83,84]. The stability, localization, and activity of these molecules are regulated by, among others, N-glycosylation, phosphorylation, ubiquitination, S-palmitoylation, and disulfide bond formation. These posttranslational modifications can influence receptor and ligand stability, trafficking, cell-surface expression, and intracellular signaling, thereby affecting the availability and functional activity of the CTLA-4/CD28–CD80/CD86 axis. In particular, ubiquitination and phosphorylation of CTLA-4 contribute to the regulation of its intracellular trafficking and surface availability, which may influence the strength and duration of CTLA-4-mediated inhibition [82,83,84]. An integrated characterization of the membrane topology, domain organization, posttranslational modification sites, sequence variants, and functional regions of CTLA-4, CD28, CD80, and CD86 is presented in Figure 4 [31,39,40,51]. The isoforms of individual axis components are summarized in Supplementary Tables S3 and S4.

**Figure 5:**
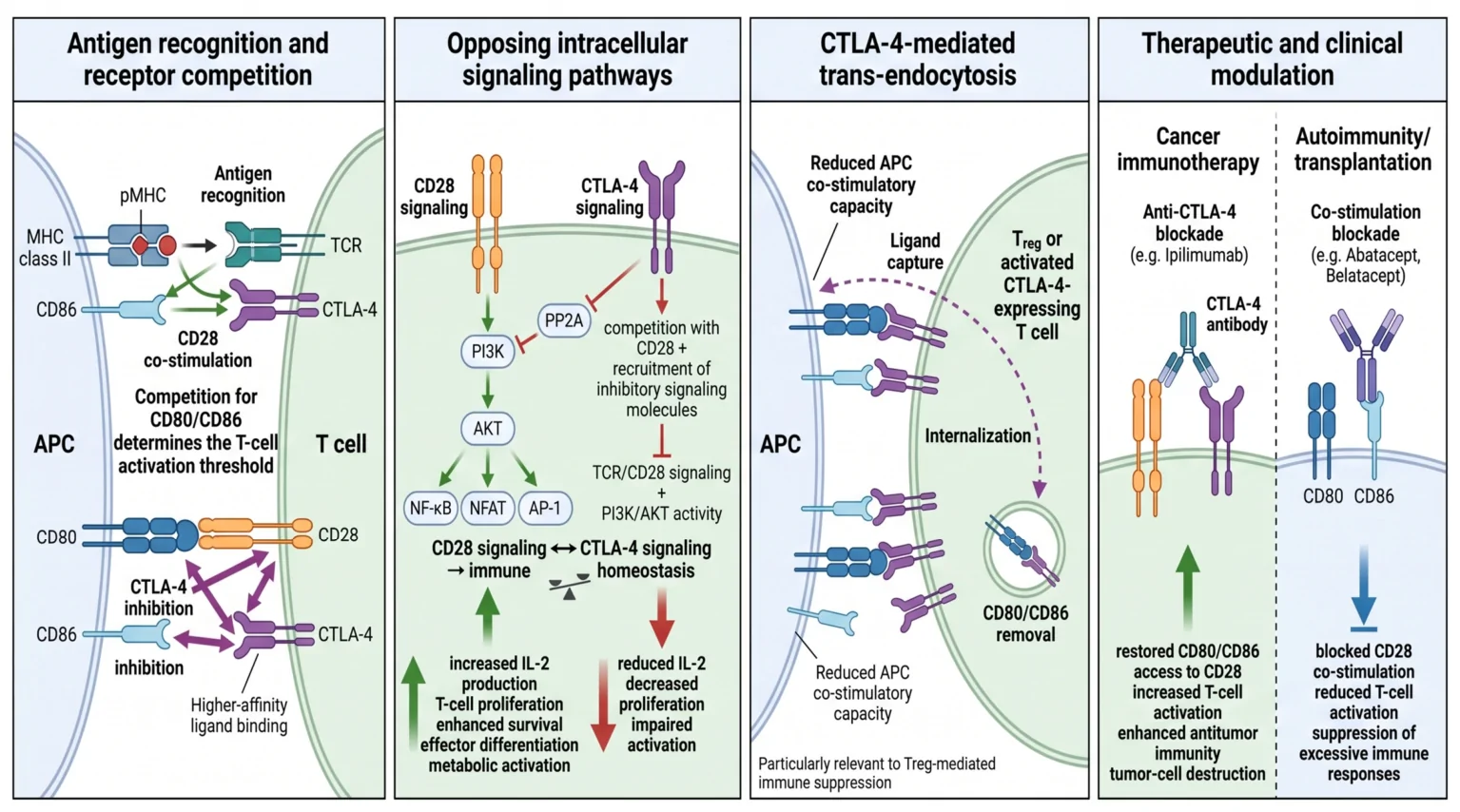
Functional organization and therapeutic modulation of the CTLA-4/CD28–CD80/CD86 immune checkpoint axis [83,84,85,86]. The figure illustrates antigen recognition and competition between CD28 and CTLA-4 for CD80/CD86 ligands, the opposing intracellular signaling pathways mediated by CD28 and CTLA-4, CTLA-4-mediated trans-endocytosis of CD80/CD86, and therapeutic modulation of this axis in cancer immunotherapy and autoimmune diseases/transplantation. Solid arrows indicate the direction of receptor–ligand interactions, intracellular signaling, or functional effects. Green arrows indicate stimulatory or restorative effects associated with enhanced T-cell activation, whereas red arrows and inhibitory lines indicate suppressive effects or attenuation of T-cell activation. Purple arrows indicate CTLA-4-associated inhibitory interactions and ligand capture; dashed purple arrows indicate CTLA-4-mediated trans-endocytosis/internalization of CD80/CD86. Blue arrows indicate the functional consequences of co-stimulation blockade. The dashed vertical line in the therapeutic panel separates the two therapeutic contexts: cancer immunotherapy and autoimmunity/transplantation. The balance symbol represents immune homeostasis resulting from the balance between CD28-mediated co-stimulatory signaling and CTLA-4-mediated inhibitory signaling. Abbreviations: AKT, protein kinase B; APC, antigen-presenting cell; AP-1, activator protein 1; CD28, cluster of differentiation 28; CD80, cluster of differentiation 80; CD86, cluster of differentiation 86; CTLA-4, cytotoxic T-lymphocyte-associated protein 4; IL-2, interleukin-2; MHC class II, major histocompatibility complex class II; NFAT, nuclear factor of activated T cells; NF-κB, nuclear factor kappa B; PI3K, phosphoinositide 3-kinase; pMHC, peptide–major histocompatibility complex; PP2A, protein phosphatase 2A; TCR, T-cell receptor; Treg, regulatory T cell. Created in BioRender. Mertowski, S. (2026) https://BioRender.com/5zo4a5t.

In the tumor microenvironment, the predominance of CTLA-4-dependent signaling, particularly within tumor-infiltrating regulatory T cells, leads to limited CD28-dependent costimulation, impaired expansion of effector T cells, and reduced antitumor activity. Consequently, a local immunosuppressive environment develops, promoting tumor progression [83,85]. The mechanism of CD28 and CTLA-4 competition for CD80/CD86, its consequences for T cell activation, and the potential for therapeutic modulation are presented in Figure 5 [83,84,85,86].

A different mechanism occurs in some T-cell malignancies, including adult T-cell leukemia/lymphoma (ATL). CTLA4–CD28 and ICOS–CD28 gene fusions, as well as CD28-activating mutations, have been described, leading to constitutive activation of costimulatory signals. These changes enhance tumor cell proliferation and survival and may be associated with a more aggressive disease course [83,84,85,86].

### 4.3. BTLA/CD160-HVEM/LIGHT

**Figure 6:**
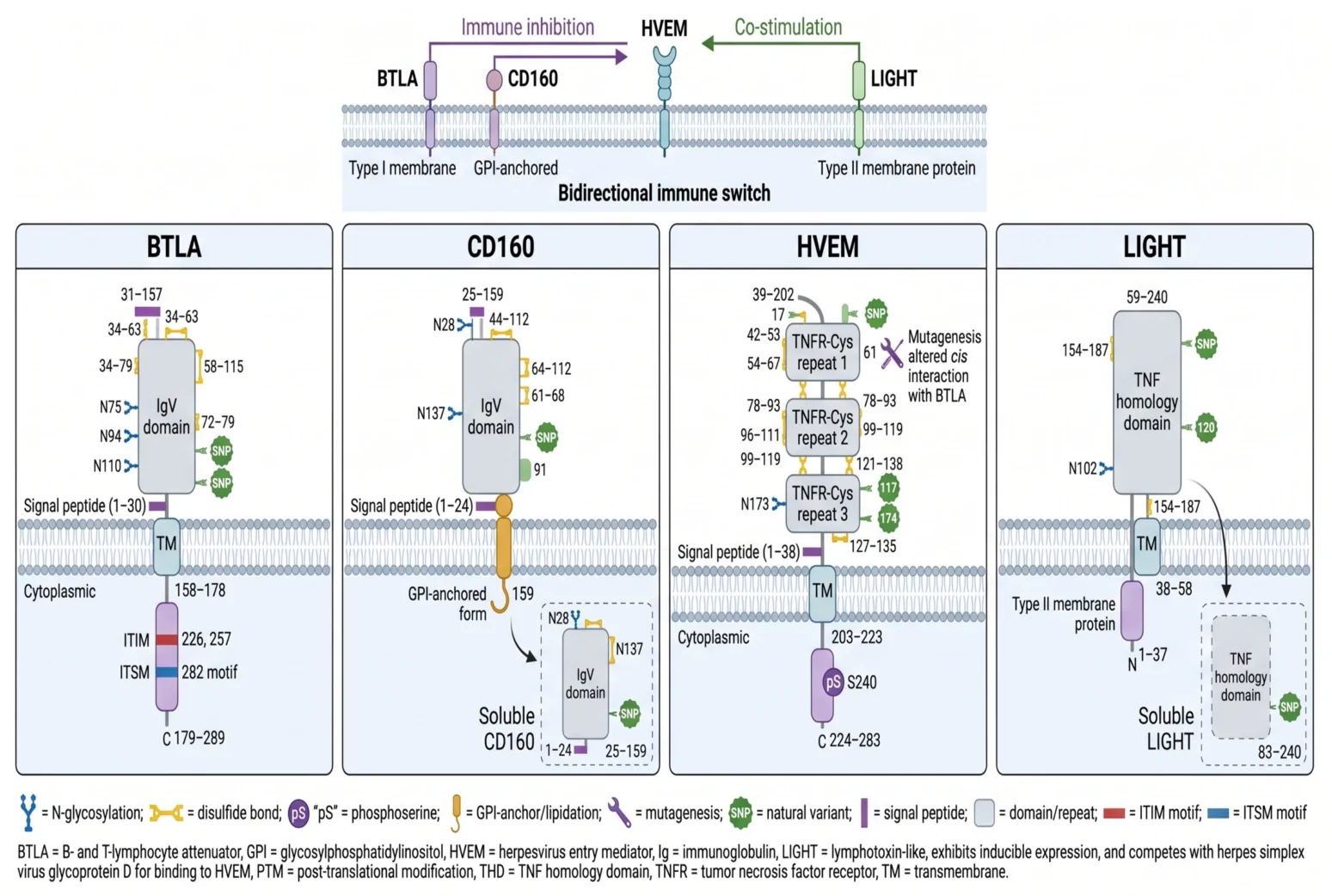
Structural and functional organization of the BTLA/CD160–HVEM/LIGHT immune checkpoint axis [38,48,50,63]. The schematic illustrates the bidirectional immune switch mediated by HVEM. Interactions of BTLA and CD160 with HVEM are associated with immune-inhibitory signaling, whereas the interaction of LIGHT with HVEM promotes co-stimulatory signaling. Purple arrows indicate immune-inhibitory signaling, whereas the green arrow indicates co-stimulatory signaling. The lower panels illustrate the structural organization, membrane topology, selected post-translational modifications, and natural variants of BTLA, CD160, HVEM, and LIGHT. Abbreviations: BTLA, B- and T-lymphocyte attenuator; CD160, cluster of differentiation 160; GPI, glycosylphosphatidylinositol; HVEM, herpesvirus entry mediator; IgV, immunoglobulin-like V-type domain; ITIM, immunoreceptor tyrosine-based inhibitory motif; ITSM, immunoreceptor tyrosine-based switch motif; LIGHT, homologous to lymphotoxins, inducible expression, and competes with herpes simplex virus glycoprotein D for HVEM, a receptor expressed by T lymphocytes; N, N-terminus; pS, phosphoserine; SNP, single-nucleotide polymorphism; THD, TNF homology domain; TM, transmembrane; TNFR-Cys, tumor necrosis factor receptor cysteine-rich repeat. Created in BioRender. Mertowski, S. (2026) https://BioRender.com/5zo4a5t.

The BTLA/CD160-HVEM/LIGHT axis constitutes a complex regulatory network integrating co-inhibitory and co-stimulatory signals. Due to its ability to transmit opposing biological signals, it is sometimes referred to as a bidirectional immune switch. This system connects molecules of the immunoglobulin superfamily, BTLA and CD160, to the HVEM receptor, a member of the tumor necrosis factor receptor superfamily, and to its ligand, LIGHT. Depending on the type of interaction, the cis or trans configuration, and the cellular context, this axis can limit immune cell activation or enhance the effector response, contributing to the maintenance of homeostasis, immune tolerance, and antitumor immunity [87,88,89,90]. Individual components of the axis exhibit distinct expression profiles. BTLA is found primarily on B lymphocytes, but also on CD4^+^ and CD8^+^ T lymphocytes, NK cells, dendritic cells, and macrophages. Its expression increases following activation of naive T lymphocytes and may subsequently decrease with further cell differentiation. CD160 is present primarily on NK cells, NKT cells, γδ T lymphocytes, and cytotoxic CD8^+^ T lymphocytes, particularly those with the CD28^−^ phenotype, while it is present to a lesser extent on CD4^+^ T lymphocytes. HVEM is characterized by broad expression, encompassing naive T and B lymphocytes, dendritic cells, monocytes, and neutrophils, among others. LIGHT expression, on the other hand, is inducible and increases primarily following T cell activation and on selected dendritic cell populations [87,88,89]. Functional differences between the axis components also result from their structural organization. BTLA is a type I transmembrane receptor containing an extracellular IgV domain and cytoplasmic signaling motifs enabling the transmission of inhibitory signals. The extracellular IgV domain mediates interactions with HVEM, whereas the cytoplasmic ITIM and ITSM motifs determine the ability of BTLA to recruit intracellular phosphatases and transmit inhibitory signals [87,88,89]. CD160 contains an IgV domain and can exist as either a membrane-anchored protein via a GPI anchor or a soluble form. The GPI anchor localizes CD160 to the outer leaflet of the plasma membrane and may facilitate its organization within membrane microdomains, whereas soluble CD160 has a different distribution and may contribute to systemic rather than strictly cell-contact-dependent signaling. HVEM is a type I transmembrane receptor containing extracellular cysteine-rich repeats characteristic of TNFRSF, while LIGHT is a type II membrane protein belonging to the TNF ligand superfamily and can also be released in a soluble form. The cysteine-rich domains of HVEM form the extracellular interaction surface for its ligands, while the membrane anchoring of HVEM and LIGHT determines their availability for cell-surface receptor–ligand interactions. The stability, localization, and activity of these molecules are further modulated by N-glycosylation, disulfide bond formation, phosphorylation, lipidation, and proteolytic processing. An integrated characterization of the membrane topology, domain organization, post-translational modifications, sequence variants, and functional regions of BTLA, CD160, HVEM, and LIGHT is presented in Figure 6 [38,48,50,63]. The described and potential isoforms of the individual axis components are summarized in Supplementary Tables S5 and S6.

**Figure 7:**
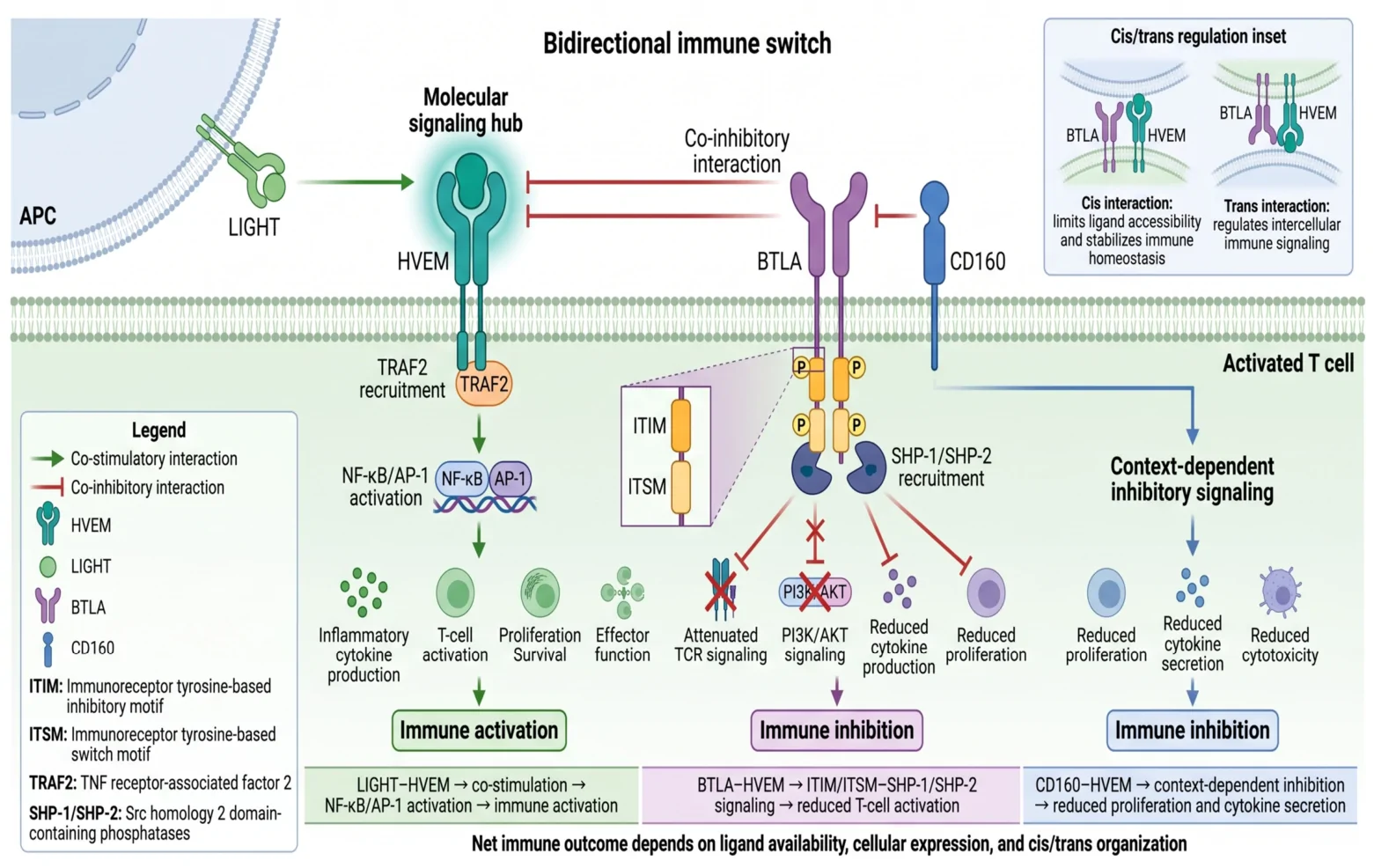
Bidirectional regulation of immune responses by the BTLA/CD160–HVEM/LIGHT axis [87,88,89,90,91,92]. The figure illustrates the bidirectional immune switch mediated by HVEM and its interactions with LIGHT, BTLA, and CD160. LIGHT–HVEM interaction promotes co-stimulatory signaling through TRAF2 recruitment and activation of NF-κB/AP-1, resulting in T-cell activation, proliferation, survival, effector function, and inflammatory cytokine production. In contrast, BTLA–HVEM interaction induces inhibitory signaling through phosphorylation of the intracellular ITIM and ITSM motifs and recruitment of SHP-1/SHP-2, leading to attenuation of TCR and PI3K/AKT signaling, reduced cytokine production, proliferation, and T-cell activation. CD160–HVEM interaction mediates context-dependent inhibitory signaling. Blue arrows indicate the direction and downstream consequences of CD160–HVEM-mediated context-dependent inhibitory signaling. Red cross marks indicate inhibition or suppression of the indicated downstream signaling pathways or cellular processes. Yellow circles containing the letter “P” indicate phosphorylation of intracellular receptor residues/motifs. The cis/trans regulation inset illustrates that cis interactions can limit ligand accessibility and stabilize immune homeostasis, whereas trans interactions regulate intercellular immune signaling. Abbreviations: AKT, protein kinase B; AP-1, activator protein 1; APC, antigen-presenting cell; BTLA, B- and T-lymphocyte attenuator; CD160, cluster of differentiation 160; HVEM, herpesvirus entry mediator; ITIM, immunoreceptor tyrosine-based inhibitory motif; ITSM, immunoreceptor tyrosine-based switch motif; LIGHT, homologous to lymphotoxins, inducible expression, and competes with herpes simplex virus glycoprotein D for HVEM, a receptor expressed by T lymphocytes; NF-κB, nuclear factor kappa B; PI3K, phosphoinositide 3-kinase; SHP-1, Src homology 2 domain-containing protein tyrosine phosphatase 1; SHP-2, Src homology 2 domain-containing protein tyrosine phosphatase 2; TCR, T-cell receptor; TRAF2, TNF receptor-associated factor 2. Created in BioRender. Mertowski, S. (2026) https://BioRender.com/5zo4a5t.

The central element of this network is HVEM (TNFRSF14), which can act as both a receptor and a binding partner for molecules with distinct biological properties. Interaction of HVEM with BTLA initiates a coinhibitory signal dependent on the ITIM and ITSM motifs in the cytoplasmic domain of BTLA. Phosphorylation of these motifs enables the recruitment of SHP-1 and SHP-2 phosphatases, leading to attenuation of TCR-dependent signaling, reduced proliferation, and reduced cytokine production by T lymphocytes. Interaction between HVEM and CD160 can also inhibit immune cell activation, but its effects depend on the cell type, CD160 isoform, and microenvironmental conditions [86,87,88,89]. HVEM binding to LIGHT has the opposite effect. This interaction leads to the recruitment of TRAF family adaptor proteins and activation of the NF-κB and AP-1 pathways, promoting the survival, proliferation, and differentiation of effector cells and increasing the production of proinflammatory cytokines. The biological effect of HVEM activation depends on the type of ligand, the relative expression level of individual molecules, their distribution on cells, and competition between binding partners. Thus, structural features that determine membrane localization and ligand accessibility can directly influence which HVEM interaction predominates in a given cellular context [65,66]. The bidirectional mechanism of immune response regulation via the BTLA/CD160–HVEM/LIGHT axis is illustrated in Figure 7 [87,88,89,90,91,92].

An additional level of regulation is the ability of BTLA and HVEM to form complexes in cis and trans configurations. Cis interactions occur between molecules on the surface of the same cell and can limit HVEM access to ligands on neighboring cells. This arrangement stabilizes the quiescent state and prevents uncontrolled activation. Upon cell activation, changes in expression and membrane organization enable trans-interactions between BTLA or CD160 and HVEM present on another cell, allowing for dynamic adjustment of the immune response [90]. Therefore, membrane organization and the relative accessibility of binding partners represent important structural determinants of the functional output of this axis. The importance of the BTLA/CD160–HVEM/LIGHT axis is particularly evident in the tumor microenvironment. Increased expression of BTLA and CD160 on tumor-infiltrating lymphocytes may be associated with an exhaustion phenotype, limited cytokine production, and impaired cytotoxicity. This phenomenon has been described in melanoma, lung cancer, hepatocellular carcinoma, and lymphomas, among others. Elevated levels of soluble BTLA may also be associated with an unfavorable prognosis. In turn, disruption of BTLA inhibitory signaling in autoimmune diseases may promote loss of tolerance and excessive lymphocyte activation. In chronic viral infections, including hepatitis B virus (HBV), cytomegalovirus (CMV), human immunodeficiency virus (HIV), and severe acute respiratory syndrome coronavirus 2 (SARS-CoV-2), increased expression of BTLA and CD160 is associated with persistent antigenic stimulation and the development of T lymphocyte dysfunction [89,90,91,92].

### 4.4. CD226/TIGIT/CD96/CD112R-CD155/CD112

PVR axis constitutes a complex network regulating the activity of NK cells and T lymphocytes. It comprises the activating receptor CD226 (DNAM-1) and inhibitory or context-dependent receptors such as TIGIT, CD112R (PVRIG), and CD96, which compete for the shared ligands CD155 (PVR) and CD112 (Nectin-2/PVRL2). Under physiological conditions, the balance between CD226-dependent activating signaling and inhibitory signals mediated by TIGIT, CD112R, and, to some extent, CD96, enables control of effector functions and maintenance of immune homeostasis. In the tumor microenvironment, however, this balance shifts toward immunosuppression, promoting effector cell dysfunction and tumor escape from immune surveillance [93,94,95,96]. The central ligands of this axis are CD155 and CD112, members of the nectin and nectin-like molecule family. In addition to participating in cell adhesion and the organization of intercellular junctions, they regulate communication between immune cells and target cells. Their expression can be increased in many cancers, including melanoma, lung cancer, breast cancer, ovarian cancer, pancreatic cancer, hepatocellular carcinoma, gliomas, and acute myeloid leukemia. However, increased ligand availability does not necessarily enhance the activating response, as TIGIT and CD112R can bind CD155 and CD112 with greater affinity than CD226, thereby preferentially activating inhibitory pathways [94,95,96,97]. CD226 is the main activating receptor of this network and is expressed primarily on NK cells, CD8^+^ T cells, some CD4^+^ T cells, monocytes, and dendritic cells. Binding to CD155 or CD112 promotes immunological synapse formation, cytoskeletal reorganization, degranulation, and the production of IFN-γ and TNF-α. Activation of CD226, therefore, enhances the cytotoxicity of NK and T cells and promotes the elimination of tumor cells [93,94,95,96,97].

TIGIT is a coinhibitory receptor present on NK cells, CD8^+^ and CD4^+^ T cells, regulatory T cells, NKT cells, and follicular helper T cells (Tfh). It exhibits high affinity for CD155 and a weaker affinity for CD112, effectively competing with CD226 for access to ligands. Upon binding to CD155, the ITIM- and ITT-like motifs in the cytoplasmic domain of TIGIT are phosphorylated, recruiting adaptor molecules and the SHIP-1 phosphatase. This leads to attenuation of the PI3K/AKT, MAPK, and NF-κB pathways, reduced cytokine production, and reduced cytotoxic activity of NK cells and T lymphocytes [94,95,96].

CD112R (PVRIG) is a coinhibitory receptor with a relatively limited ligand profile, as its best-characterized partner is CD112. This receptor is expressed primarily on NK cells and effector and memory CD8^+^ T cells, while its expression on naive cells is low. CD112R binds CD112 with higher affinity than CD226, thereby limiting activating signaling. In the tumor microenvironment, increased expression of CD112R on tumor-infiltrating lymphocytes and NK cells often coexists with PD-1 and TIGIT, suggesting a role for CD112R in perpetuating the effector cell exhaustion phenotype [97,98].

CD96 (TACTILE) binds primarily to CD155 and is expressed primarily on NK cells and T cells, including selected Treg populations. Unlike TIGIT, its function is ambiguous and varies across species, cell types, and microenvironmental conditions. Both inhibitory effects, associated with limited cytotoxicity, and costimulatory properties, influencing proliferation and cytokine production, have been described. Therefore, CD96 should be considered a receptor with a context-dependent function, not a purely coinhibitory one [95,96,97].

**Figure 8:**
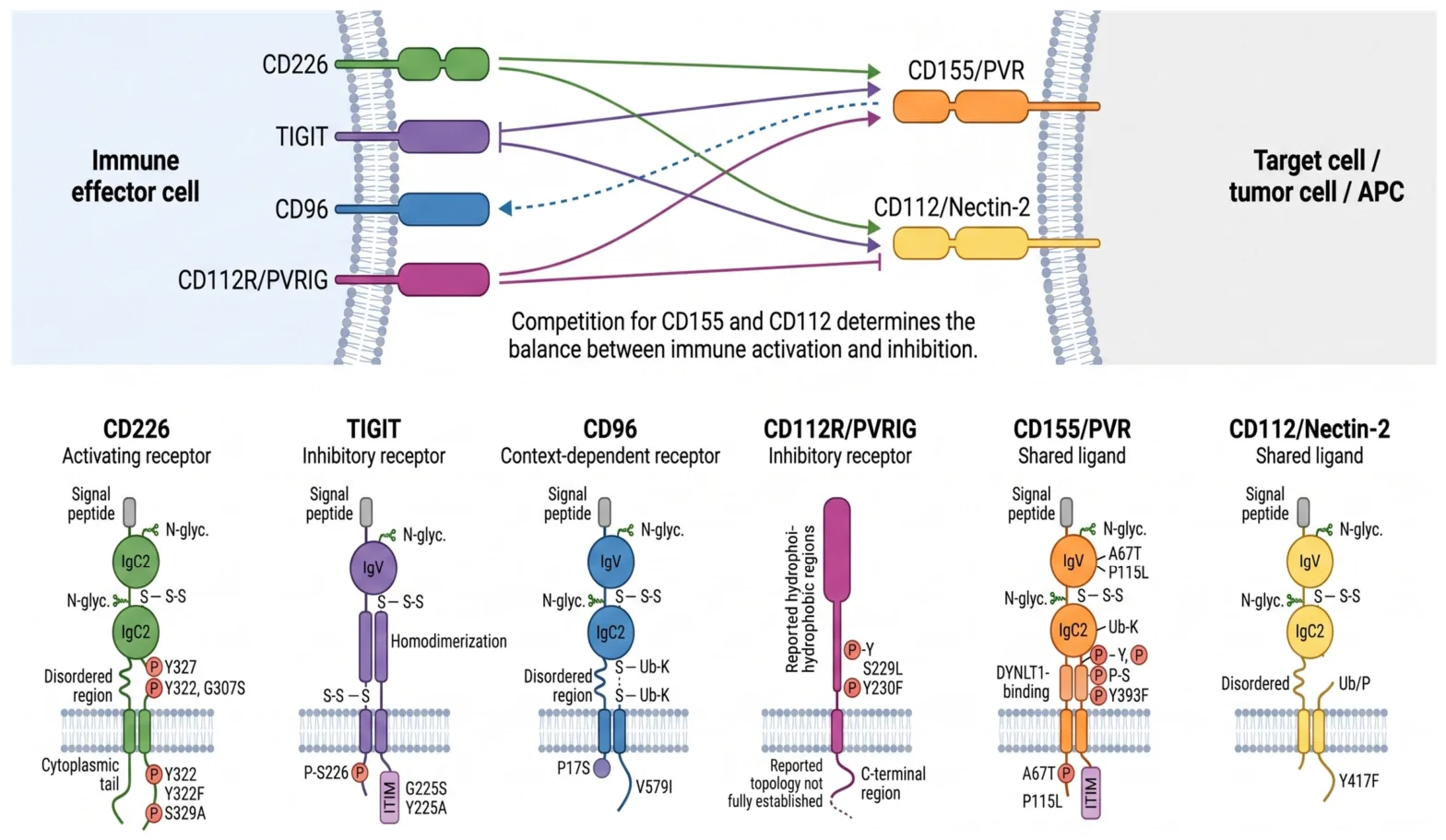
Structural and functional organization of the CD226/TIGIT/CD96/CD112R–CD155/CD112 immune checkpoint axis [37,53,57,68,69]. Solid colored arrows indicate receptor–ligand interactions within the checkpoint network, whereas the dashed blue arrow indicates the context-dependent CD96–CD155/PVR interaction. Structural annotations and post-translational modifications are defined in the abbreviations below. Abbreviations: APC, antigen-presenting cell; CD112, cluster of differentiation 112; CD112R, CD112 receptor; CD155, cluster of differentiation 155; CD226, cluster of differentiation 226; CD96, cluster of differentiation 96; DYNLL1, dynein light chain LC8-type 1; IgC2, immunoglobulin-like C2-type domain; IgV, immunoglobulin-like V-type domain; ITIM, immunoreceptor tyrosine-based inhibitory motif; N-glyc., N-glycosylation; PVR, poliovirus receptor; PVRIG, poliovirus receptor-related immunoglobulin domain-containing protein; pS, phosphoserine; pT, phosphothreonine; pY, phosphotyrosine; S–S, disulfide bond; TIGIT, T-cell immunoreceptor with immunoglobulin and ITIM domains; TM, transmembrane; Ub, ubiquitination. Created in BioRender. Mertowski, S. (2026) https://BioRender.com/5zo4a5t.

The functional properties of the PVR axis components are closely related to their structural organization. CD226 contains two extracellular Ig-like C2 domains, TIGIT contains a single IgV domain and a cytoplasmic ITIM motif, whereas CD96 has an extensive extracellular portion comprising two IgV domains and one IgC2 domain. CD155 and CD112 each contain one IgV domain and two IgC2 domains, enabling them to form extensive receptor–ligand interaction networks. The extracellular immunoglobulin domains form the principal receptor–ligand binding interfaces, and differences in their organization can affect receptor accessibility, binding stoichiometry, and affinity. Structural studies have shown that the ligand-binding surface of CD96 can be partially occluded in its homodimeric state, whereas the corresponding surface of TIGIT remains accessible, resulting in differences in receptor–ligand complex formation [97,98,99]. Individual proteins undergo numerous post-translational modifications, including N-glycosylation, phosphorylation, ubiquitination, and disulfide bond formation, which influence their stability, localization, and signaling capacity. Consequently, structural features that alter the accessibility or organization of extracellular binding surfaces may influence the functional competition between PVR family receptors [97,98,99]. An integrated characterization of the membrane topology, domain organization, post-translational modifications, sequence variants, and functional regions of CD226, TIGIT, CD96, CD112R, CD155, and CD112 is presented in Figure 8 [37,53,57,68,69]. The described and predicted isoforms of individual axis components are summarized in Supplementary Tables S7 and S8.

**Figure 9:**
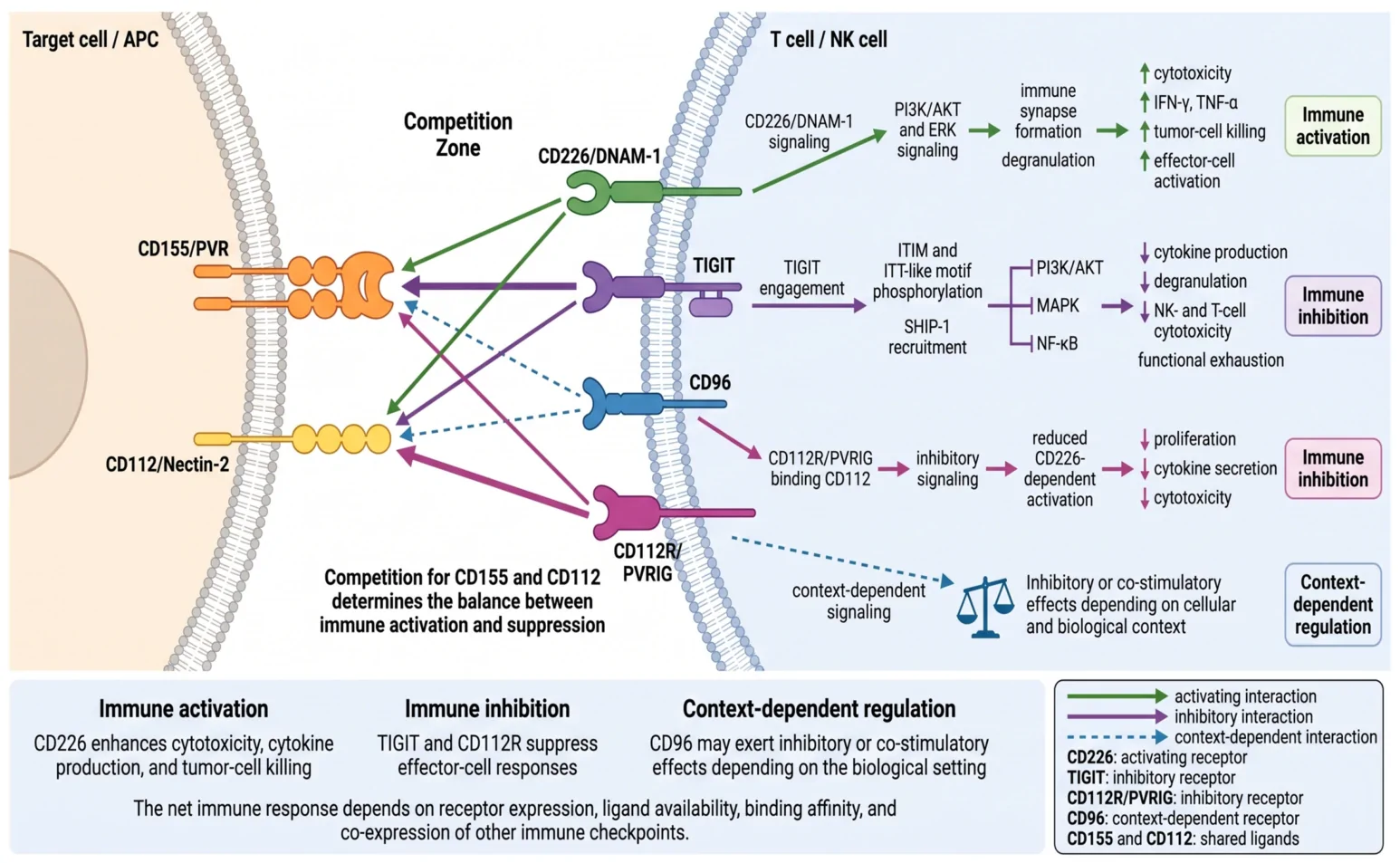
Competitive regulation of activating and inhibitory signaling within the PVR immune checkpoint network [95,96,97,98,99,100,101,102,103]. The figure illustrates competition among CD226/DNAM-1, TIGIT, CD96, and CD112R/PVRIG for the shared ligands CD155/PVR and CD112/Nectin-2 and the resulting balance between immune activation and inhibition. CD226/DNAM-1 signaling promotes PI3K/AKT- and ERK-dependent immune activation, whereas TIGIT and CD112R/PVRIG mediate inhibitory signaling. CD96 exerts context-dependent regulatory effects. The pink arrows indicate inhibitory signaling initiated by CD112R/PVRIG binding to CD112/Nectin-2, resulting in reduced CD226-dependent activation and decreased proliferation, cytokine secretion, and cytotoxicity. The balance symbol represents the context-dependent equilibrium between inhibitory and co-stimulatory effects, which depends on the cellular and biological context. Abbreviations: AKT, protein kinase B; APC, antigen-presenting cell; CD112, cluster of differentiation 112; CD112R, CD112 receptor; CD155, cluster of differentiation 155; CD226, cluster of differentiation 226; CD96, cluster of differentiation 96; DNAM-1, DNAX accessory molecule 1; ERK, extracellular signal-regulated kinase; IFN-γ, interferon gamma; ITIM, immunoreceptor tyrosine-based inhibitory motif; ITT-like, immunoglobulin tail tyrosine-like motif; MAPK, mitogen-activated protein kinase; NF-κB, nuclear factor kappa B; NK, natural killer; PI3K, phosphoinositide 3-kinase; PVR, poliovirus receptor; PVRIG, poliovirus receptor-related immunoglobulin domain-containing protein; SHIP-1, SH2 domain-containing inositol 5-phosphatase 1; TIGIT, T-cell immunoreceptor with immunoglobulin and ITIM domains; TNF-α, tumor necrosis factor alpha. Created in BioRender. Mertowski, S. (2026) https://BioRender.com/5zo4a5t.

A key mechanism regulating the functioning of this network is the competition between activating and inhibitory receptors for common ligands. CD226, TIGIT, and CD96 compete primarily for CD155, while CD226 and CD112R compete for CD112. Importantly, experimental measurements indicate that the relative strength of these interactions depends on the receptor–ligand pair and the experimental configuration. In particular, monovalent interactions within the PVR family can display relatively weak affinities, indicating that differences in surface expression, receptor organization, and avidity may contribute substantially to the functional dominance of individual pathways rather than affinity alone [98,99,100,101,102]. Under conditions of chronic antigenic stimulation, with increased expression of TIGIT and CD112R and increased availability of CD155 and CD112 in the tumor microenvironment, inhibitory signals predominate and CD226 function gradually declines. This results in reduced proliferation, cytokine production, and cytotoxic activity of NK cells and T lymphocytes [99,100,101,102]. The mechanism of competitive interactions within the PVR axis and their impact on the balance between activation and inhibition of the immune response is presented in Figure 9 [95,96,97,98,99,100,101,102,103].

### 4.5. TIM3-Galactin-9-CEACAM1

The TIM-3/Galectin-9/CEACAM1 axis is a complex regulatory system that controls the activity of T lymphocytes and other effector cells. Unlike simple receptor–ligand systems, TIM-3 function depends on interactions among several binding partners and co-receptors, the best-characterized of which are Galectin-9 and CEACAM1. Activation of this axis promotes the limitation of the effector response, the maintenance of immune tolerance, and the resolution of excessive inflammation. However, under conditions of chronic antigenic stimulation, it may contribute to the development of T cell exhaustion and immunosuppression in the tumor microenvironment [104,105,106,107].

TIM-3 is a type I membrane glycoprotein belonging to the immunoglobulin superfamily. It is expressed primarily on activated Th1 and Th17 cells, as well as on CD8 cytotoxic T cells, NK cells, monocytes, macrophages, dendritic cells, mast cells, and regulatory T cells. TIM-3 expression increases during prolonged antigenic stimulation and often coexists with other coinhibitory receptors on cells with an exhausted phenotype, especially in chronic infections and malignancies [104,105,106,107]. Galectin-9 is a tandem β-galactoside-binding lectin composed of two carbohydrate recognition domains (CRDs). This protein lacks a classical signal peptide; therefore, it can be released via unconventional secretion mechanisms, including extracellular vesicles and lysosomal pathways. Galectin-9 is widely expressed in immune cells and numerous tissues, and its elevated levels have also been observed in many cancers, including acute myeloid leukemia, breast cancer, hepatocellular carcinoma, and gliomas [105,106,107].

**Figure 10:**
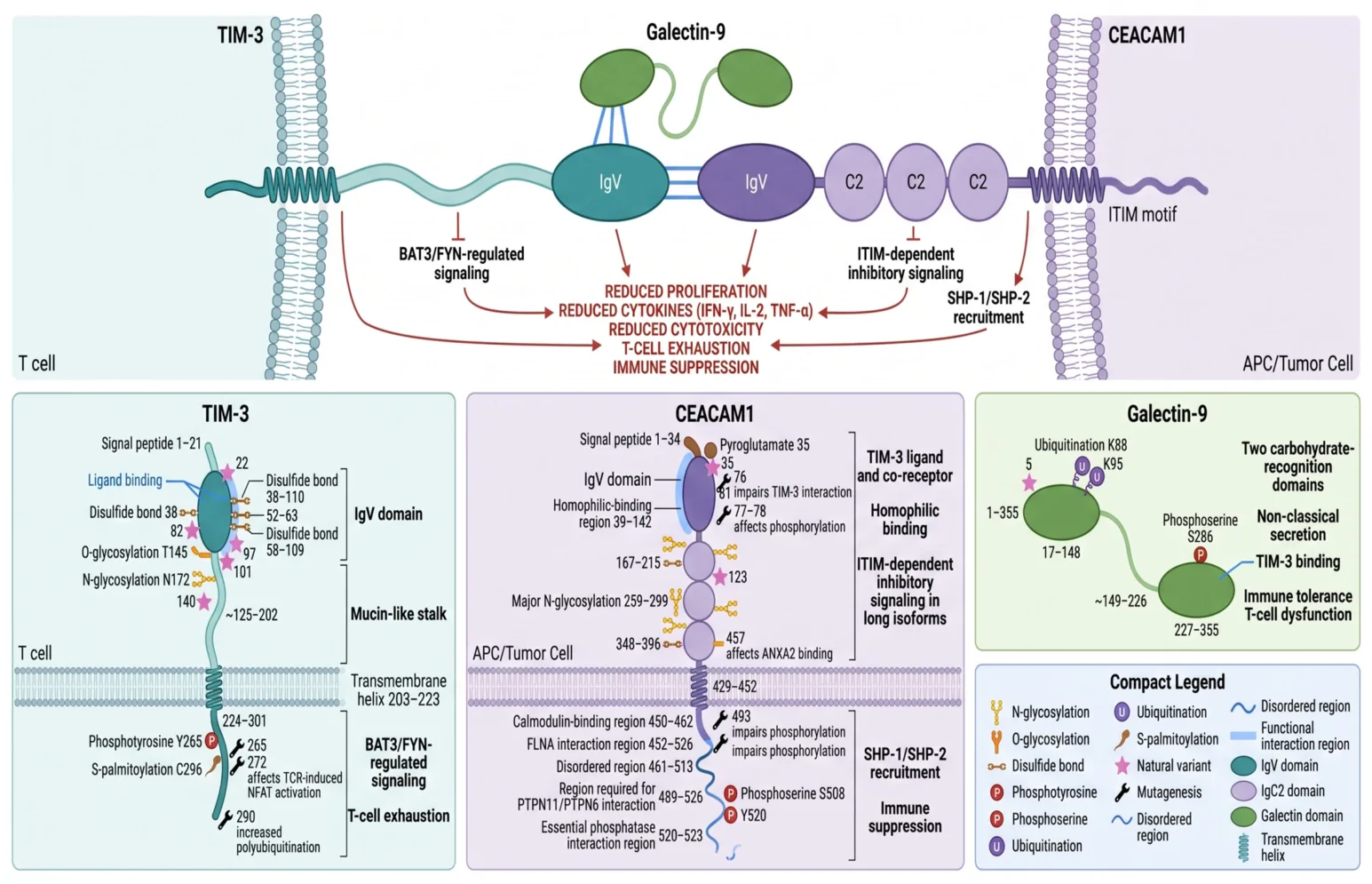
Structural and functional organization of the TIM-3/Galectin-9/CEACAM1 immune checkpoint axis [55,58,66]. The figure illustrates the interactions among TIM-3, Galectin-9, and CEACAM1 and their contribution to inhibitory immune signaling. TIM-3-associated BAT3/FYN-regulated signaling and CEACAM1-mediated ITIM-dependent signaling with SHP-1/SHP-2 recruitment converge on suppression of T-cell responses. Red lines indicate inhibitory signaling or functional relationships leading to reduced T-cell proliferation, reduced cytokine production, reduced cytotoxicity, T-cell exhaustion, and immune suppression. Red text highlights the major inhibitory functional outcomes of the TIM-3/Galectin-9/CEACAM1 axis, whereas blue text identifies ligand-binding or interaction-related structural features. The lower panels illustrate the structural organization, functional regions, selected post-translational modifications, and molecular interactions of TIM-3, CEACAM1, and Galectin-9. Abbreviations: ANXA2, annexin A2; APC, antigen-presenting cell; BAT3, HLA-B-associated transcript 3; C2, immunoglobulin-like C2-type domain; CEACAM1, carcinoembryonic antigen-related cell adhesion molecule 1; FLNA, filamin A; FYN, FYN proto-oncogene, Src family tyrosine kinase; IFN-γ, interferon gamma; IgV, immunoglobulin-like V-type domain; IL-2, interleukin 2; ITIM, immunoreceptor tyrosine-based inhibitory motif; NFAT, nuclear factor of activated T cells; PTPN11, protein tyrosine phosphatase non-receptor type 11; SHP-1, Src homology 2 domain-containing protein tyrosine phosphatase 1; SHP-2, Src homology 2 domain-containing protein tyrosine phosphatase 2; TCR, T-cell receptor; TIM-3, T-cell immunoglobulin and mucin-domain containing-3; TNF-α, tumor necrosis factor alpha. Created in BioRender. Mertowski, S. (2026) https://BioRender.com/5zo4a5t.

CEACAM1 is a transmembrane adhesion glycoprotein present on T and B lymphocytes, NK cells, monocytes, macrophages, dendritic cells, and epithelial, endothelial, and tumor cells. Alternative splicing leads to the formation of isoforms differing in the length of their cytoplasmic domains. Long CEACAM1 isoforms contain ITIM motifs that enable the recruitment of SHP-1 and SHP-2 phosphatases and the transduction of inhibitory signals, whereas short isoforms may exhibit distinct signaling properties [106,107]. The structural organization of the axis components crucially determines their function. TIM-3 contains an extracellular IgV domain, a mucin-like segment, a single transmembrane segment, and a cytoplasmic signaling tail. The IgV domain contains distinct ligand-binding surfaces: glycosylated residues contribute to recognition by Galectin-9, whereas the FG-CC′ region forms a binding site for phosphatidylserine and has also been implicated in CEACAM1 recognition. Thus, the structural organization and glycosylation state of the IgV domain can directly influence ligand accessibility and receptor binding [107,108,109]. CEACAM1 has one IgV domain and three IgC2 domains in its extracellular portion, whereas Galectin-9 consists of two galectin domains connected by a linker region. The tandem organization of Galectin-9 carbohydrate-recognition domains enables interaction with glycosylated molecular surfaces and is therefore directly relevant to its recognition of TIM-3 [107,108,109]. The stability and activity of these proteins are modulated by glycosylation, phosphorylation, ubiquitination, S-palmitoylation, and disulfide bond formation. In particular, CEACAM1 expression has been shown to influence TIM-3 maturation, protein stability, and cell-surface localization, indicating that the interaction between these molecules may affect not only signaling but also the amount of functionally available TIM-3 at the cell surface [107,108,109]. An integrated characterization of the membrane topology, domain organization, posttranslational modifications, sequence variants, and functional regions of TIM-3, Galectin-9, and CEACAM1 is presented in Figure 10 [55,58,66]. The described and predicted isoforms of these molecules are summarized in Supplementary Tables S9 and S10.

The mechanism of action of the TIM-3/Galectin-9/CEACAM1 axis depends on the type of ligand and the cellular context. In the resting state, BAT3 interacts with the cytoplasmic portion of TIM-3 and supports proper T cell signaling. Ligand binding can lead to tyrosine phosphorylation in the cytoplasmic tail of TIM-3, BAT3 dissociation, and recruitment of Src family kinases, including FYN. This results in attenuation of TCR-dependent signaling and a gradual reduction in effector functions. However, this mechanism should not be interpreted as uniform and linear for all cell types, as TIM-3 can exert different effects depending on the ligand, expression level, and activation status of the cell [104,105,106,110,111,112,113]. Galectin-9 is the best-studied TIM-3 ligand. Its binding to the IgV domain of the receptor can disrupt calcium homeostasis and lead to reduced activity or induction of Th1 cell death. This is accompanied by reduced secretion of IFN-γ, IL-2, and TNF-α, as well as impaired proliferation and cytotoxic function. With chronic activation of the axis, these changes promote the development of a T cell exhaustion phenotype [110,111,112,113].

CEACAM1 plays a more complex role, acting both as a TIM-3 ligand and as a co-receptor influencing its maturation, stability, and surface expression. TIM-3–CEACAM1 interactions can occur in both cis and trans configurations, resulting in enhanced inhibitory signaling and reduced proliferation and cytokine production by T lymphocytes. However, experimental evidence concerning direct TIM-3–CEACAM1 binding and the functional relevance of cis and trans interactions is not fully consistent, indicating that these interactions may depend on the cellular and experimental context. Long CEACAM1 isoforms additionally transmit their own co-inhibitory signals via ITIM motifs and SHP-1/SHP-2 phosphatases [109,110,111,112].

**Figure 11:**
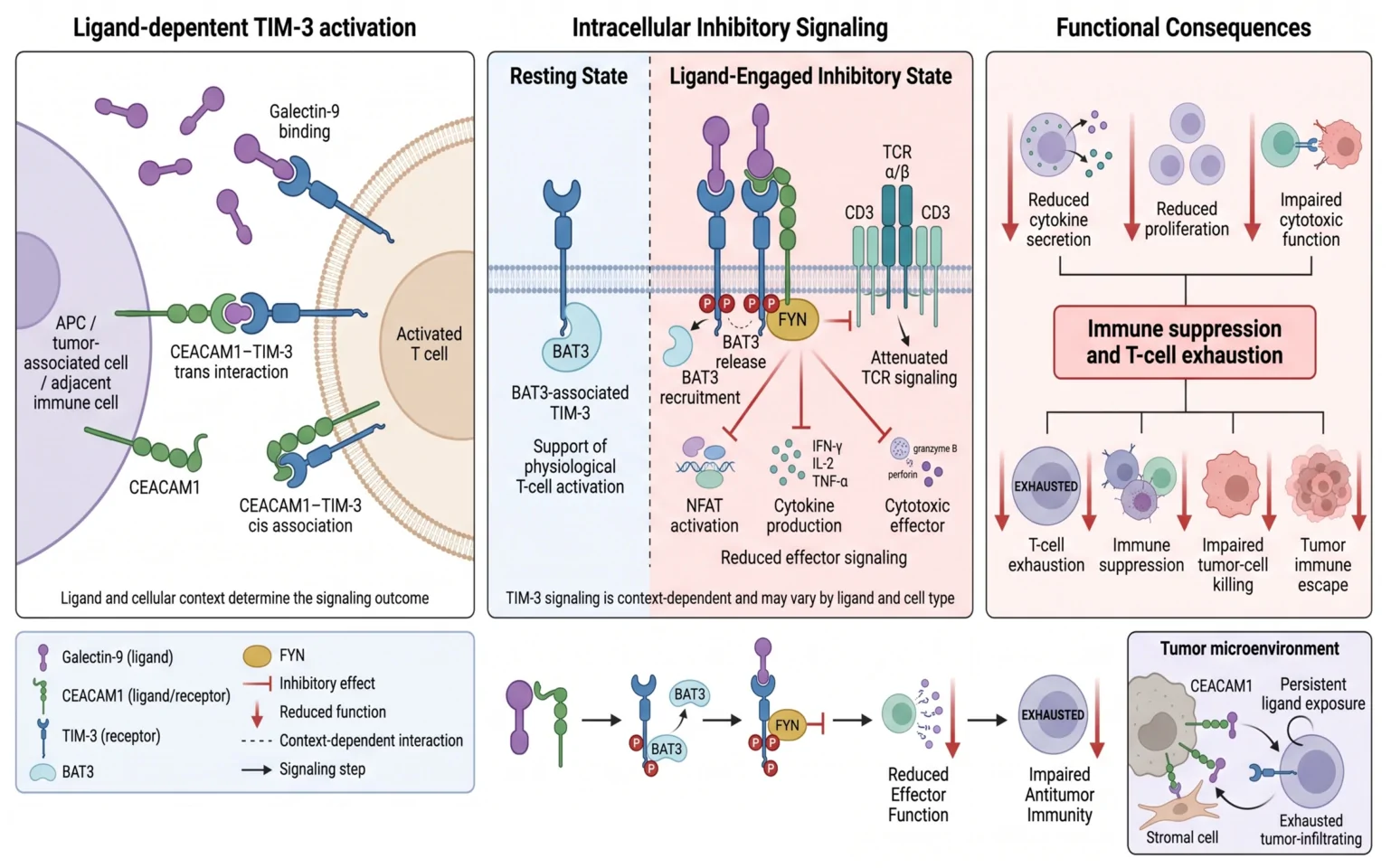
Molecular mechanisms of TIM-3-dependent signaling leading to T-cell exhaustion [104,105,106,107,108,109,110,111,112,113,114,115]. Abbreviations: APC, antigen-presenting cell; BAT3, HLA-B-associated transcript 3; CEACAM1, carcinoembryonic antigen-related cell adhesion molecule 1; FYN, FYN proto-oncogene, Src family tyrosine kinase; IFN-γ, interferon gamma; IL-2, interleukin 2; NFAT, nuclear factor of activated T cells; TCR, T-cell receptor; TIM-3, T-cell immunoglobulin and mucin-domain containing-3; TNF-α, tumor necrosis factor alpha, Red circles containing the letter “P” indicate phosphorylation of intracellular receptor residues. Created in BioRender. Mertowski, S. (2026) https://BioRender.com/5zo4a5t.

TIM-3 also interacts with phosphatidylserine and HMGB1. Phosphatidylserine binding is involved in the recognition of apoptotic cells and the regulation of phagocytosis, while interaction with HMGB1 may limit dendritic cell activation by disrupting the transport of nucleic acids into endosomes and attenuating signaling through Toll-like receptors [105,110,114]. The structural specificity of the TIM-3 IgV domain is also relevant to therapeutic antibody recognition. Structural analysis of the TIM-3–MBG220 complex showed that the antibody binds the GFCC′ surface of the IgV domain, close to regions involved in ligand recognition, illustrating how the three-dimensional organization of the receptor can determine antibody epitope accessibility and potentially interfere with ligand binding [106,107,108,109,110]. The signaling mechanisms of the TIM-3 axis and their relationship to T cell dysfunction are presented in Figure 11 [104,105,106,107,108,109,110,111,112,113,114,115]. Under physiological conditions, the TIM-3/Galectin-9/CEACAM1 axis supports immune tolerance and limits excessive inflammatory responses, including during pregnancy and the cessation phase following antigen elimination. However, in the tumor microenvironment, these same mechanisms can be utilized to suppress the activity of cytotoxic lymphocytes and natural killer cells. Increased expression of Galectin-9 and CEACAM1 on tumor cells and other microenvironment components promotes TIM-3 activation and perpetuates local immunosuppression [104,105,106,107,108,109,110,115].

### 4.6. LAG3-FGL1/MHC II

The LAG-3/FGL1–MHC class II axis constitutes an important co-inhibitory mechanism regulating the activation, proliferation, and effector functions of T lymphocytes. LAG-3 is a receptor in the immunoglobulin superfamily whose activation limits cellular responses and supports the maintenance of immune tolerance. Unlike many other checkpoints, LAG-3 is a multiligand receptor. Its best-characterized partners include MHC class II molecules and FGL1, while interactions have also been described for LSECtin and Galectin-3. Depending on the cellular context and ligand availability, LAG-3 participates in both the physiological suppression of the immune response and the development of immunosuppression associated with cancer and chronic infections [116,117,118,119]. LAG-3 is a type I transmembrane glycoprotein encoded by the LAG3 gene located on chromosome 12. The extracellular portion of the receptor comprises four immunoglobulin-like domains, termed D1–D4, connected to a single transmembrane segment and a cytoplasmic signaling tail. Domain D1 contains the region responsible for MHC class II binding, while domains D1 and D2 are primarily involved in the interaction with FGL1. Structural studies have further shown that LAG-3 forms cis-homodimers mediated primarily by the D2 domain and that this oligomeric organization influences the spatial orientation of the ligand-binding D1 domains. Disruption of the D2 dimerization interface markedly reduces LAG-3 binding to both MHC class II and FGL1, demonstrating that a domain not directly forming the principal ligand-binding surface can nevertheless regulate ligand accessibility and receptor function [116,117,118,119]. The cytoplasmic portion of LAG-3 contains conserved regulatory elements, including RRFSALE and KIEELE motifs, as well as a C-terminal region rich in glutamate repeats. These elements participate in the inhibition of TCR-dependent signaling, although the full intracellular mechanism of LAG-3 remains less clearly defined than that of PD-1 or BTLA [111,112,113,114]. LAG-3 expression is primarily observed on activated CD4^+^ and CD8^+^ T cells, regulatory T cells, NK and NKT cells, activated B lymphocytes, and plasmacytoid dendritic cells. During an acute immune response, increased receptor expression serves as a negative feedback mechanism. However, during chronic antigen stimulation, LAG-3 remains elevated, particularly on tumor-infiltrating lymphocytes, where it often coexists with PD-1, TIGIT, and TIM-3. This coexpression profile is characteristic of cells with reduced proliferation, limited cytokine production, and attenuated cytotoxicity [116,117,118,119,120].

**Figure 12:**
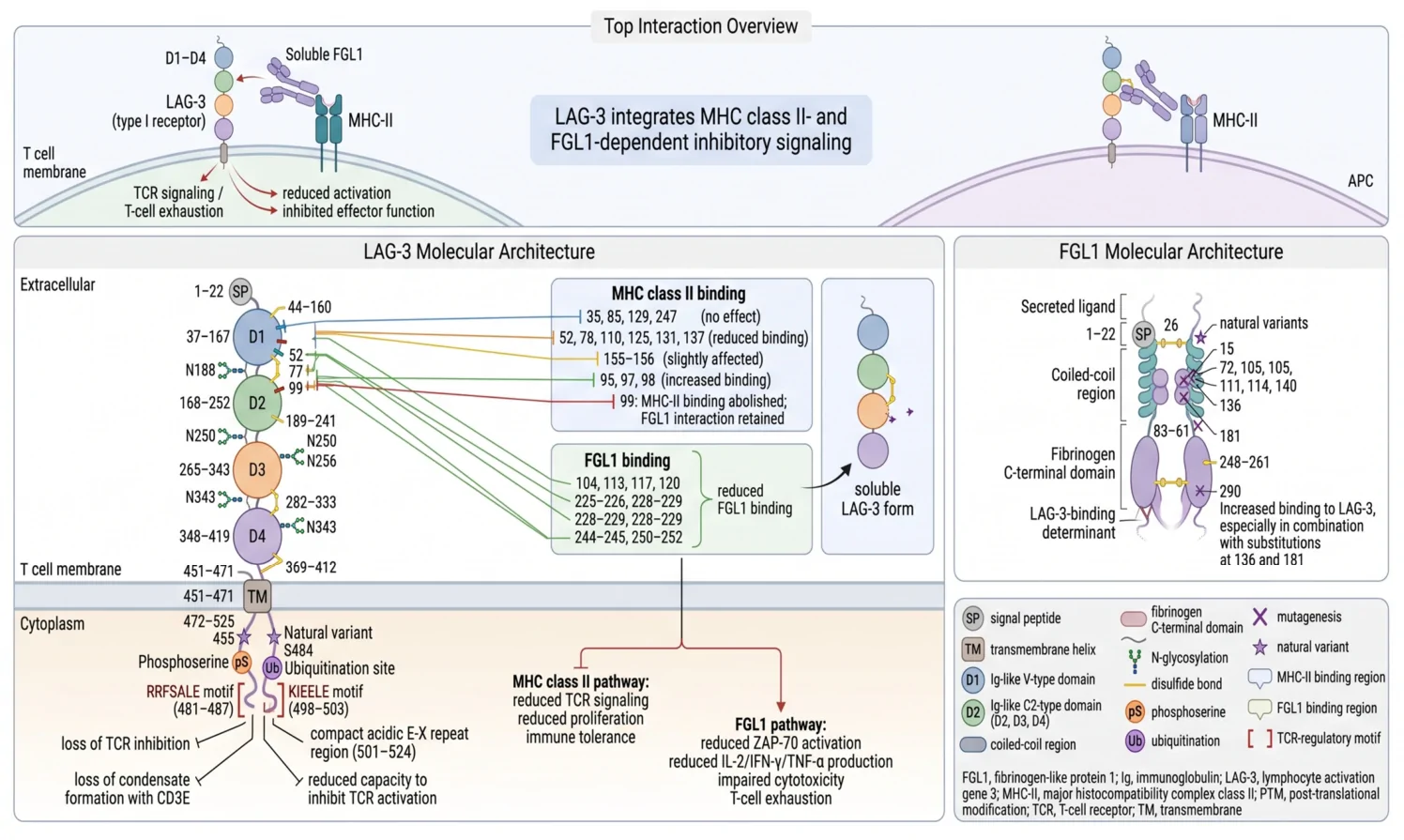
Structural and functional organization of the LAG-3/FGL1–MHC class II immune checkpoint axis [54,67]. The figure illustrates the molecular architecture of LAG-3 and FGL1, their interaction with MHC class II and FGL1, and the associated inhibitory effects on T-cell signaling and function. Arrows indicate the direction of molecular interactions, signaling, or downstream functional consequences. Red text highlights the intracellular RRFSALE and KIEELE regulatory motifs of LAG-3 associated with modulation of TCR signaling. The yellow connected-circle symbol indicates a disulfide bond. The extracellular LAG-3 region comprises four immunoglobulin-like domains: D1 is an Ig-like V-type domain, whereas D2, D3, and D4 are Ig-like C2-type domains. Abbreviations: APC, antigen-presenting cell; CD3E, CD3 epsilon subunit; D1–D4, extracellular immunoglobulin-like domains 1–4; FGL1, fibrinogen-like protein 1; IgC2, immunoglobulin-like C2-type domain; IgV, immunoglobulin-like V-type domain; IL-2, interleukin 2; IFN-γ, interferon gamma; LAG-3, lymphocyte activation gene 3; MHC-II, major histocompatibility complex class II; PTM, post-translational modification; SP, signal peptide; TCR, T-cell receptor; TM, transmembrane; TNF-α, tumor necrosis factor alpha; Ub, ubiquitination. Created in BioRender. Mertowski, S. (2026) https://BioRender.com/5zo4a5t.

The structure and molecular properties of LAG-3 and FGL1 determine their biological functions. LAG-3 contains four immunoglobulin-like domains, a junctional region, a single transmembrane helix, and a cytoplasmic tail containing conserved signaling motifs and disordered regions. The receptor is N-glycosylated, phosphorylated, and ubiquitinated and can exist in membrane-bound and soluble forms. Importantly, structural analyses of glycosylated LAG-3 have demonstrated that a protein–glycan interaction contributes to the D2 dimerization interface and affects the orientation of the neighboring D1 domain, providing a direct link between posttranslational modification, receptor architecture, ligand binding, and inhibitory function [116,117,118,119,120]. FGL1 is a secreted protein containing an N-terminal coiled-coil region and a C-terminal fibrinogen-like domain responsible for interaction with LAG-3. The C-terminal fibrinogen-like domain contains the principal LAG-3-binding surface, with several surface-exposed residues in its P subdomain contributing to receptor interaction. An integrated characterization of the topology, domain organization, posttranslational modifications, sequence variants, and functional regions of LAG-3 and FGL1 is presented in Figure 12 [54,67]. The described and predicted isoforms of the individual components of this axis are summarized in Supplementary Tables S11 and S12.

**Figure 13:**
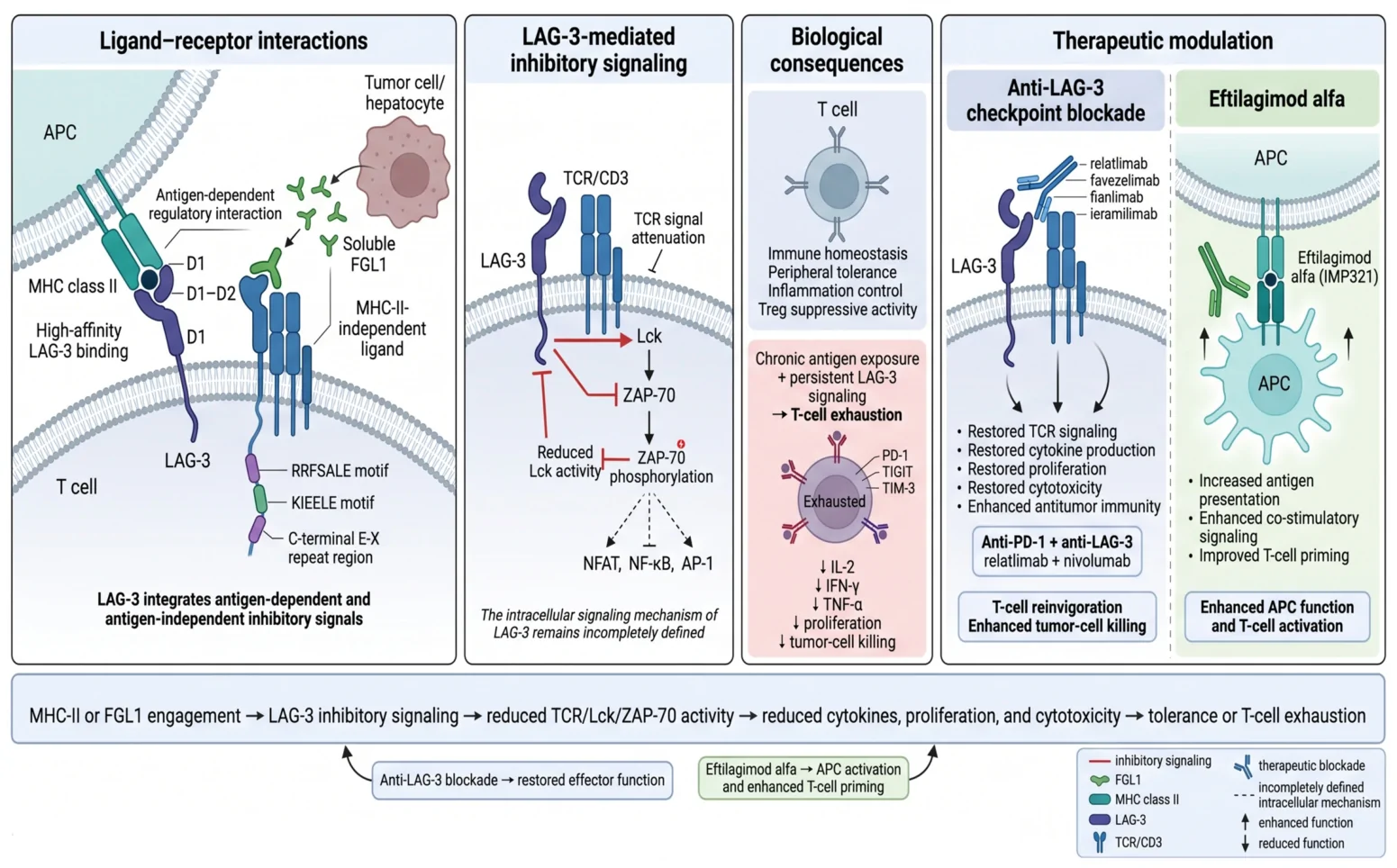
Mechanism of action and therapeutic modulation of the LAG-3/FGL1–MHC class II immune checkpoint axis [116,117,118,119,120,121,122,123]. The figure illustrates ligand–receptor interactions involving LAG-3, MHC class II, and FGL1, LAG-3-mediated inhibitory signaling, its biological consequences, and therapeutic modulation of this pathway. LAG-3 engagement attenuates TCR/CD3 signaling and reduces Lck/ZAP-70 activity, thereby suppressing downstream T-cell activation and effector functions. Solid arrows and lines indicate defined molecular interactions, signaling events, or downstream functional consequences; solid red lines specifically indicate inhibitory signaling. Dashed lines indicate incompletely defined interactions or intracellular signaling mechanisms. The red circle associated with ZAP-70 indicates phosphorylation. Therapeutic blockade of LAG-3 restores TCR signaling and T-cell effector functions, whereas eftilagimod alfa enhances APC activation, antigen presentation, and T-cell priming. Abbreviations: APC, antigen-presenting cell; AP-1, activator protein 1; CD3, cluster of differentiation 3; FGL1, fibrinogen-like protein 1; IFN-γ, interferon gamma; IL-2, interleukin 2; LAG-3, lymphocyte activation gene 3; Lck, lymphocyte-specific protein tyrosine kinase; MHC-II, major histocompatibility complex class II; NFAT, nuclear factor of activated T cells; NF-κB, nuclear factor kappa B; PD-1, programmed cell death protein 1; TCR, T-cell receptor; TIGIT, T-cell immunoreceptor with immunoglobulin and ITIM domains; TIM-3, T-cell immunoglobulin and mucin-domain containing-3; TNF-α, tumor necrosis factor alpha; Treg, regulatory T cell; ZAP-70, zeta-chain-associated protein kinase 70. Created in BioRender. Mertowski, S. (2026) https://BioRender.com/5zo4a5t.

MHC class II molecules are physiological ligands for LAG-3, to which the receptor binds with greater affinity than the CD4 co-receptor. This interaction limits T cell activation and supports the maintenance of peripheral tolerance, especially under conditions of chronic antigen exposure. Unlike MHC class II, FGL1 is a ligand independent of antigen presentation and is of particular importance in cancer immunology. The protein is synthesized primarily by hepatocytes, but its expression can be significantly increased in cancer cells and in the tumor microenvironment, including lung, prostate, colon, melanoma, and hepatocellular carcinoma [121]. FGL1 binding by LAG-3 leads to inhibition of TCR-dependent signaling, reduced CD4/CD8–Lck axis activity, and reduced ZAP-70 phosphorylation. This results in reduced clonal expansion of T lymphocytes, reduced secretion of IL-2, IFN-γ, and TNF-α, and attenuated cytotoxic activity of CD8^+^ lymphocytes. In the case of MHC class II binding, LAG-3 can also modulate antigen-presenting cell functions, and the biological effect depends on the cell type, receptor and ligand expression, and co-activation of other checkpoints [119,120,121,122]. The structural basis of these interactions also indicates that the FGL1- and MHC class II-binding regions on LAG-3 are closely positioned within D1, providing a structural basis for ligand competition and for the development of antibodies capable of interfering with both interactions. Under physiological conditions, LAG-3-dependent signaling helps limit excessive inflammation, quench the response after antigen elimination, and support the suppressive activity of Treg lymphocytes. In the tumor microenvironment, chronic activation of the LAG-3/FGL1 axis can stabilize the exhausted T cell phenotype and maintain local immunosuppression. Elevated FGL1 levels and increased LAG-3 expression may be associated with poor prognosis, limited antitumor activity, and resistance to PD-1/PD-L1 axis inhibitors [114,115,116]. The mechanism of multiligand regulation of LAG-3 and its impact on TCR signaling and lymphocyte effector functions is presented in Figure 13 [116,117,118,119,120,121,122,123].

### 4.7. VISTA-PSGL-1

The VISTA/PSGL-1 axis belongs to a newer generation of immune checkpoints and plays a crucial role in regulating the activity of T lymphocytes and myeloid cells. VISTA is a glycoprotein belonging to the B7 family that can function as both a receptor and a ligand. Under physiological conditions, it helps maintain immune homeostasis and limit excessive immune activation, whereas in the tumor microenvironment it can promote local immunosuppression and attenuate the antitumor response [124,125,126,127]. VISTA expression is particularly high on cells of myeloid origin, including monocytes, macrophages, dendritic cells, and myeloid-derived suppressor cells (MDSCs). To a lesser extent, this molecule is also found on CD4^+^ and CD8^+^ T cells, as well as regulatory T cells. In numerous malignancies, VISTA is detected on both tumor-infiltrating immune cells and on tumor cells and stromal components. One of the best-characterized VISTA partners is PSGL-1, a widely expressed surface glycoprotein present on most hematopoietic cells. Interactions between VISTA and VSIG-3 have also been described, as well as the possibility of homophilic VISTA–VISTA interactions, but their full biological significance remains under investigation [124,125,126,127].

The functional properties of both components of the axis are closely related to their molecular structure. VISTA is a type I transmembrane protein that contains an extracellular IgV domain, a single transmembrane segment, and a relatively long cytoplasmic tail with numerous phosphorylation and ubiquitination sites. The extracellular IgV domain of VISTA has an unusual histidine-rich surface that forms a pH-sensitive ligand-binding region. Several surface-exposed histidine residues, particularly H153–H155, contribute to PSGL-1 recognition by becoming protonated under acidic conditions, thereby promoting electrostatic interactions with negatively charged residues of PSGL-1 [125,126,127]. PSGL-1 is a heavily glycosylated type I glycoprotein with an extensive extracellular moiety that contains tandem-repeat-rich regions, numerous N- and O-linked glycosylation sites, and sulfated tyrosine residues important for selectin binding. The extensive glycosylation and tyrosine sulfation of PSGL-1 therefore have structural consequences for ligand recognition, as negatively charged sulfated residues contribute to the interaction surface with the histidine-rich region of VISTA. Its C-terminal cytoplasmic domain contains phosphorylation sites and disordered regions that may be involved in regulating interactions with adaptor proteins and the cytoskeleton [125,126,127].

**Figure 14:**
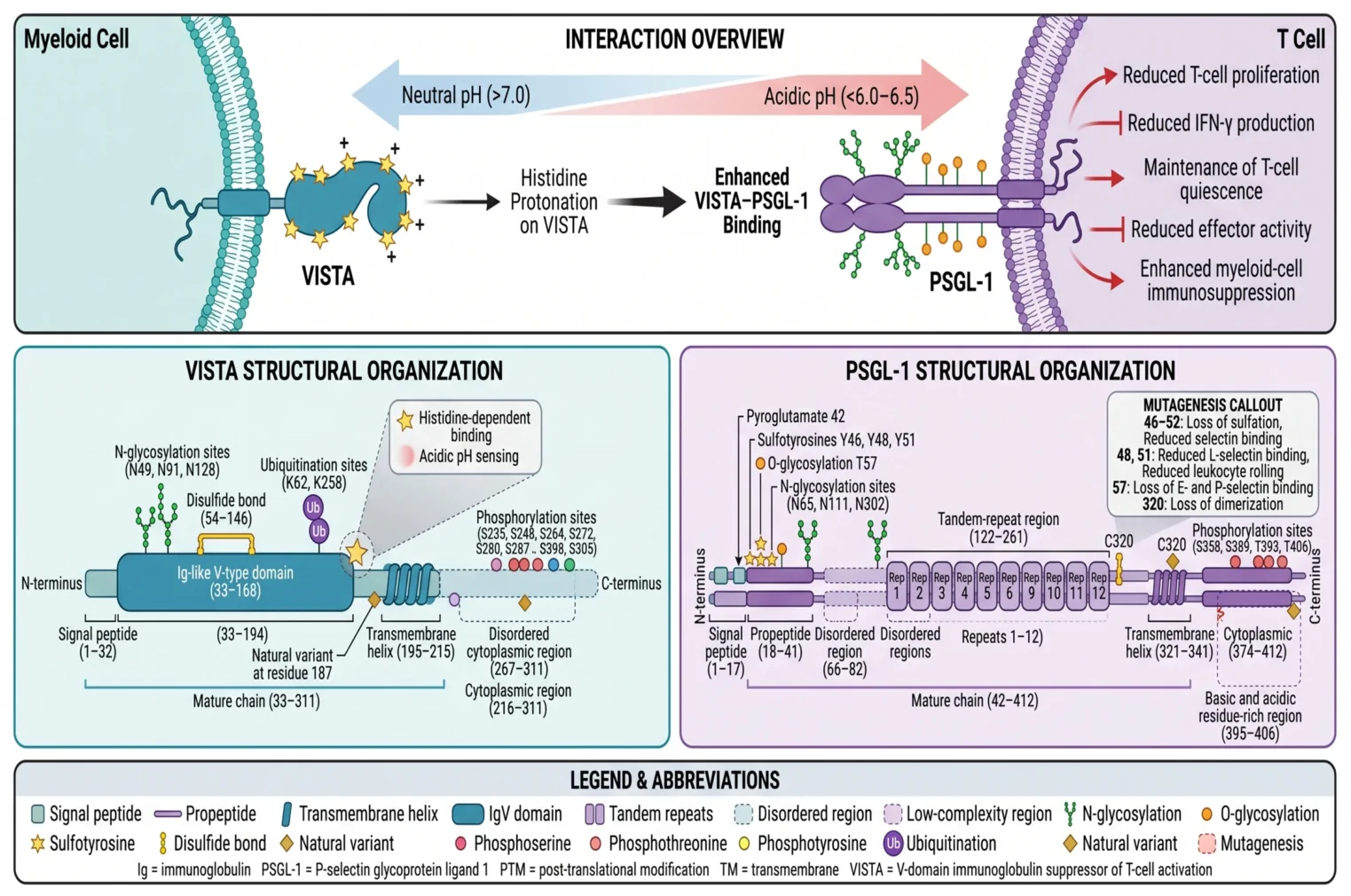
Structural and functional organization of the VISTA/PSGL-1 immune checkpoint axis [56,62]. Abbreviations: IFN-γ, interferon gamma; Ig, immunoglobulin; IgV, immunoglobulin-like V-type domain; PSGL-1, P-selectin glycoprotein ligand 1; PTM, post-translational modification; TM, transmembrane; VISTA, V-domain immunoglobulin suppressor of T-cell activation. Created in BioRender. Mertowski, S. (2026) https://BioRender.com/5zo4a5t.

An integrated characterization of the membrane topology, domain organization, posttranslational modifications, sulfation sites, sequence variants, and functional regions of VISTA and PSGL-1 is presented in Figure 14 [56,62]. The described and predicted isoforms of both molecules are summarized in Supplementary Tables S13 and S14.

**Figure 15:**
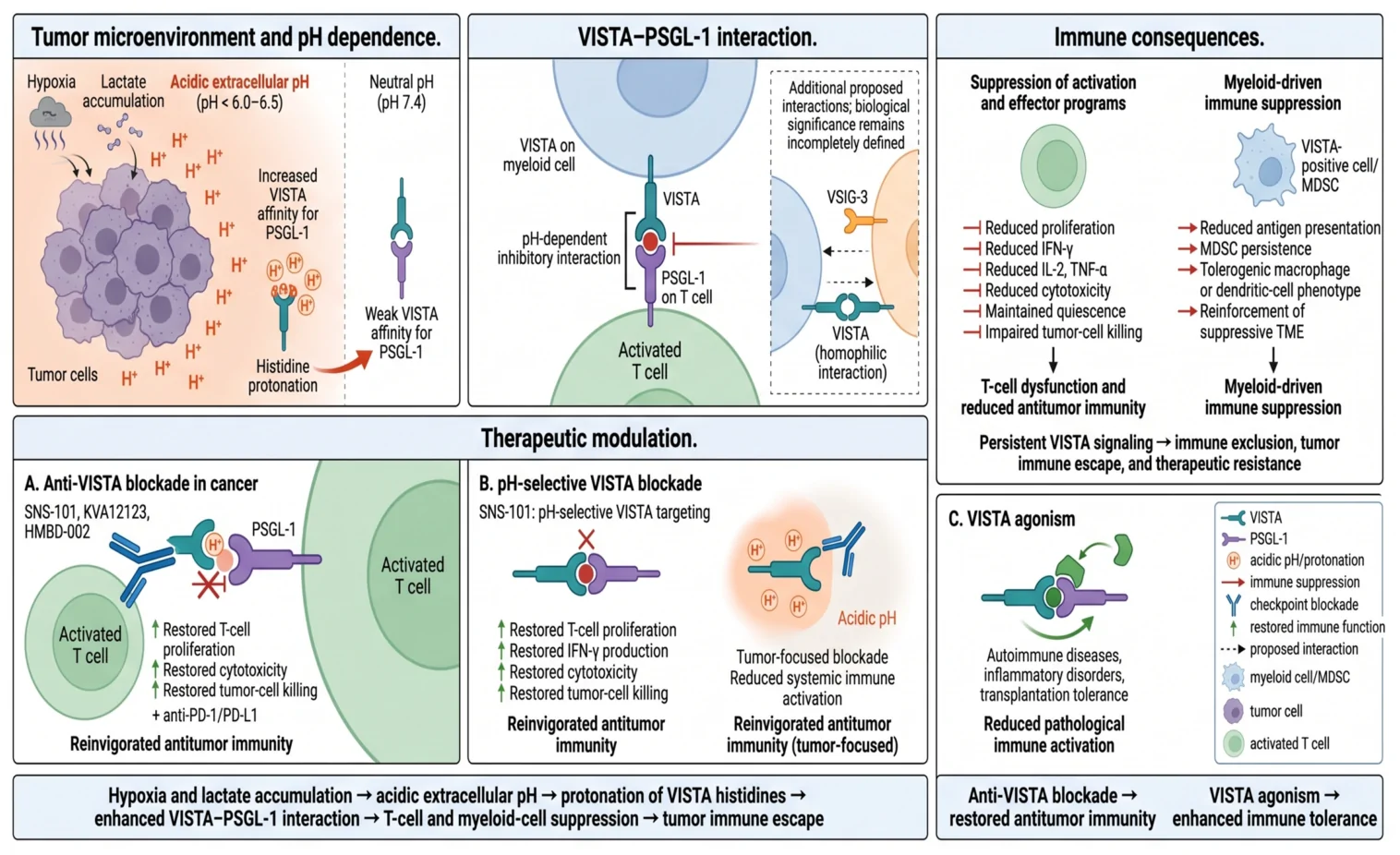
Mechanism of action and therapeutic modulation of the VISTA/PSGL-1 immune checkpoint axis [124,125,126,127,128,129]. The figure illustrates the pH-dependent regulation of VISTA–PSGL-1 interactions within the tumor microenvironment, their immunosuppressive consequences, and therapeutic approaches targeting this pathway. Acidification of the tumor microenvironment promotes histidine protonation and increases the affinity of VISTA for PSGL-1, thereby enhancing inhibitory signaling and contributing to suppression of T-cell activation and effector functions. Additional proposed VISTA interactions, including VSIG-3 and homophilic VISTA interactions, are also shown, although their biological significance remains incompletely defined. Red T-shaped symbols indicate inhibitory or suppressive effects on the indicated cellular processes or immune functions. Therapeutic strategies include anti-VISTA blockade, pH-selective VISTA blockade aimed at tumor-focused immune reactivation, and VISTA agonism to promote immune tolerance in autoimmune diseases, inflammatory disorders, and transplantation. Abbreviations: IFN-γ, interferon gamma; IL-2, interleukin 2; MDSC, myeloid-derived suppressor cell; PD-1, programmed cell death protein 1; PD-L1, programmed death-ligand 1; PSGL-1, P-selectin glycoprotein ligand 1; TME, tumor microenvironment; TNF-α, tumor necrosis factor alpha; VISTA, V-domain immunoglobulin suppressor of T-cell activation; VSIG-3, V-set and immunoglobulin domain-containing protein 3. Created in BioRender. Mertowski, S. (2026) https://BioRender.com/5zo4a5t.

A characteristic feature of the VISTA–PSGL-1 interaction is its dependence on the microenvironmental acidity. Binding of both molecules occurs preferentially under reduced pH conditions, typical of many solid tumors. This mechanism is associated with the presence of histidine residues in the extracellular portion of VISTA, whose protonation increases its affinity for PSGL-1. Consequently, the acidic tumor microenvironment may promote enhanced inhibitory signaling and selectively enhance the immunosuppressive nature of this axis [127,128,129]. Activation of VISTA/PSGL-1 leads to reduced T cell proliferation and effector function, reduced IFN-γ production, and maintenance of cells in a state of reduced activity or functional quiescence. Simultaneously, VISTA may promote a tolerogenic and immunosuppressive phenotype of myeloid cells, further limiting antigen presentation and lymphocyte activation in the tumor microenvironment. The ultimate biological effect depends on the cell type, VISTA and PSGL-1 expression level, local pH, and the presence of other checkpoints. The structural features of VISTA also have direct implications for therapeutic antibody recognition. Mutational and structural studies have identified antibody-binding epitopes within the extracellular IgV domain, some of which overlap or lie close to the PSGL-1-binding surface. In particular, pH-selective antibodies have been engineered to recognize VISTA preferentially under acidic conditions and to block VISTA–PSGL-1 interactions, demonstrating that the histidine-rich architecture of VISTA can be exploited to modify both antibody binding and checkpoint inhibition [126,127,128,129]. The mechanism of the pH-dependent VISTA–PSGL-1 interaction and its effect on T lymphocytes and myeloid cells is presented in Figure 15 [124,125,126,127,128,129].

### 4.8. OX40/OX40L

The OX40/OX40L axis is one of the most important costimulatory pathways that maintain T cell activation, survival, and differentiation. OX40 (CD134, TNFRSF4) is a receptor in the tumor necrosis factor receptor (TNFRSF) superfamily, while OX40L (CD252, TNFSF4) is a ligand in the TNF ligand superfamily. Unlike coinhibitory checkpoints such as PD-1 and CTLA-4, activation of the OX40/OX40L axis enhances the immune response, increasing T cell proliferation, survival, and effector functions. This signaling does not initiate primary lymphocyte activation but perpetuates the response initiated after TCR recognition of the antigen and transmission of a classic costimulatory signal. For this reason, OX40 is sometimes referred to as a receptor providing an additional, “fourth signal” for T cell activation [130,131,132,133,134]. OX40 is an inducible receptor. Its expression on naive T cells is low or undetectable, but increases following antigenic stimulation and typically peaks within 24–72 h. The receptor is expressed primarily on activated CD4^+^ and CD8^+^ T cells and regulatory T cells, and to a lesser extent on NK, NKT, and neutrophil cells. OX40L is expressed primarily on professional antigen-presenting cells, including dendritic cells, macrophages, and B lymphocytes. Its presence has also been demonstrated on endothelial cells, smooth muscle cells, and selected tumor cells [130,131,132,133,134].

The functional properties of both components of the axis are closely related to their structural organization. OX40 is a type I transmembrane protein whose extracellular portion contains four cysteine-rich regions characteristic of TNFRs, the third of which is truncated. Structural analysis of the human OX40-OX40L complex showed that OX40 engages the trimeric OX40L ligand through multiple cysteine-rich domains, with the interaction surface distributed across more than one interface rather than being dominated by a single binding hotspot [131,132,133]. The receptor also contains a single transmembrane segment and a cytoplasmic signaling tail, devoid of its own enzymatic activity but capable of recruiting adaptor proteins. OX40L, on the other hand, is a type II membrane protein with an extracellular domain homologous to TNF, enabling the formation of functional ligand–receptor complexes. OX40L forms a homotrimer, and three OX40 molecules can associate with one OX40L trimer to generate a higher-order OX40/OX40L complex. This oligomeric organization brings the cytoplasmic tails of OX40 into an appropriate spatial arrangement for recruitment of TRAF adaptor proteins and efficient signal transduction. The stability and activity of both proteins are modulated by N-glycosylation, disulfide bonds, phosphorylation, and the presence of disordered regions [130,133]. Thus, the cysteine-rich architecture of OX40 and the trimeric organization of OX40L are not merely structural features but directly determine receptor–ligand assembly and the spatial organization required for downstream signaling.

**Figure 16:**
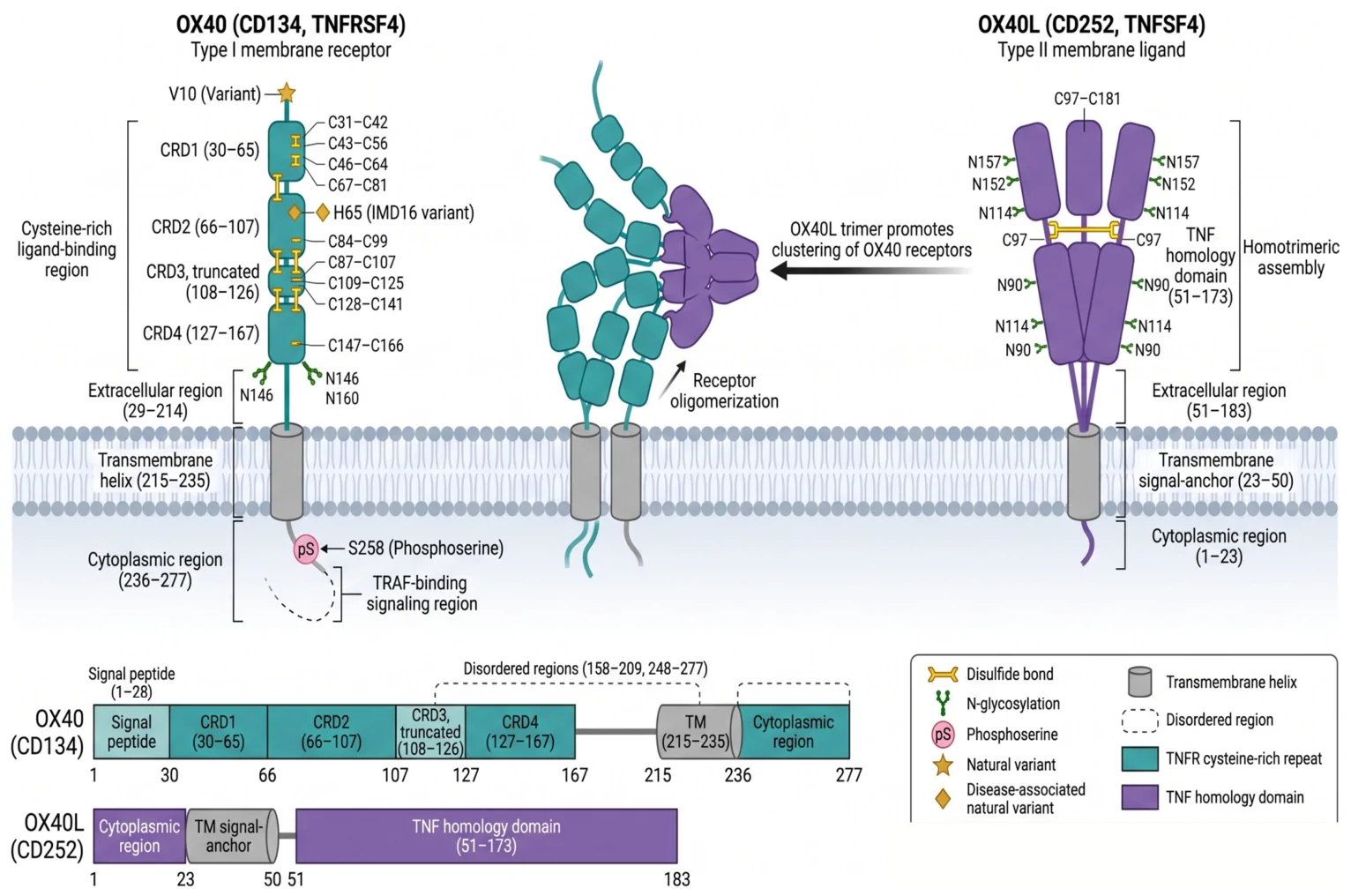
Structural and functional organization of the OX40/OX40L immune checkpoint axis [33,42]. Abbreviations: CD134, cluster of differentiation 134; CD252, cluster of differentiation 252; CRD, cysteine-rich domain; OX40, tumor necrosis factor receptor superfamily member 4; OX40L, tumor necrosis factor ligand superfamily member 4; pS, phosphoserine; PTM, post-translational modification; THD, TNF homology domain; TM, transmembrane; TNFR, tumor necrosis factor receptor; TNFRSF4, tumor necrosis factor receptor superfamily member 4; TNFSF4, tumor necrosis factor ligand superfamily member 4; TRAF, TNF receptor-associated factor. Created in BioRender. Mertowski, S. (2026) https://BioRender.com/5zo4a5t.

An integrated characterization of the membrane topology, domain organization, posttranslational modifications, sequence variants, and functional regions of OX40 and OX40L is presented in Figure 16 [33,42]. The described and predicted isoforms of both molecules are summarized in Supplementary Tables S15 and S16.

**Figure 17:**
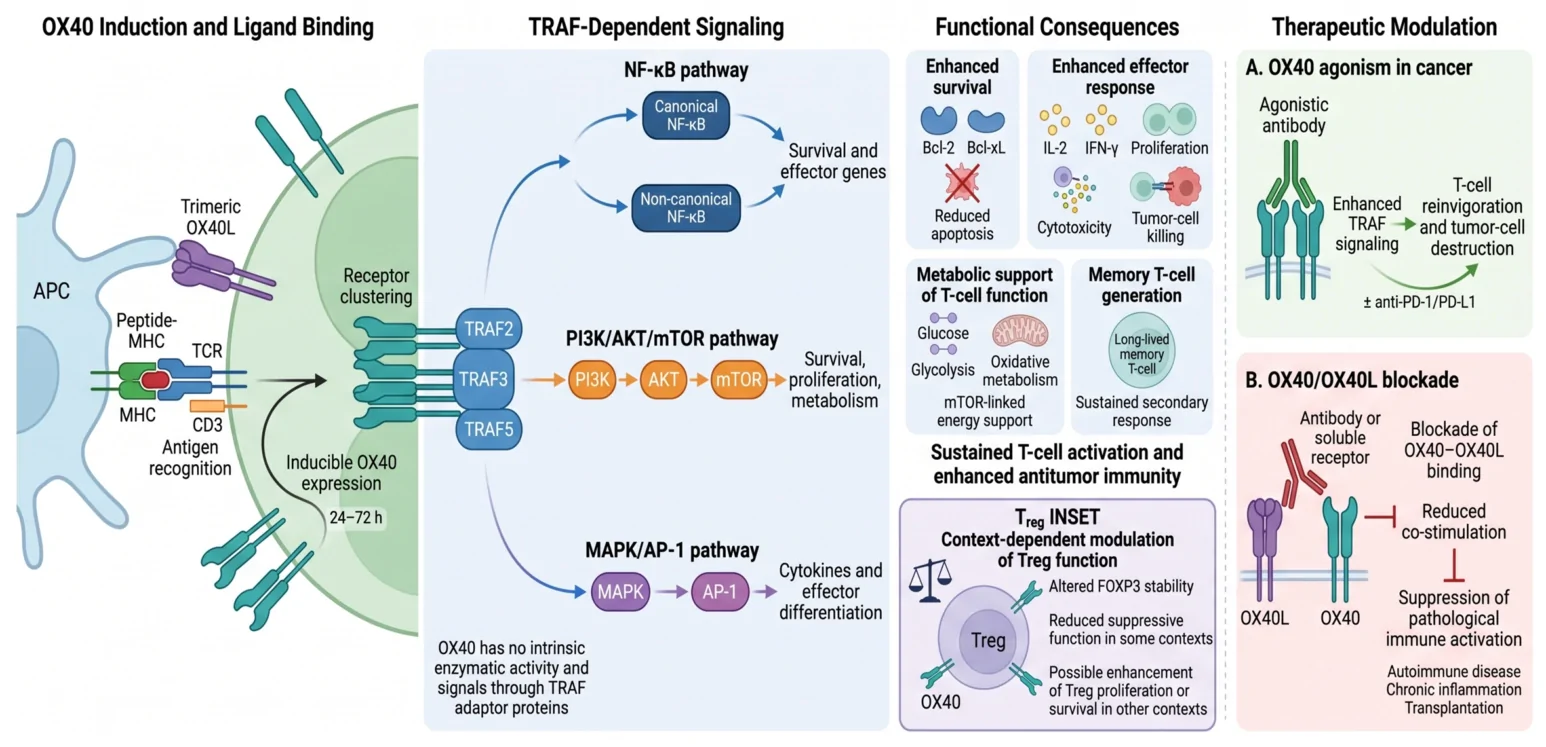
Mechanism of action and therapeutic modulation of the OX40/OX40L co-stimulatory immune checkpoint axis [130,131,132,133,134,135]. The figure illustrates OX40 induction following TCR-mediated antigen recognition, OX40L-dependent receptor clustering, TRAF-mediated intracellular signaling, functional consequences of OX40 activation, and therapeutic modulation of the OX40/OX40L pathway. OX40 signaling recruits TRAF adaptor proteins and activates the NF-κB, PI3K/AKT/mTOR, and MAPK/AP-1 pathways, promoting T-cell survival, proliferation, metabolic activity, effector function, and memory formation. Colored arrows indicate the direction of signaling and functional effects: blue arrows represent NF-κB-dependent signaling, orange arrows represent PI3K/AKT/mTOR signaling, purple arrows represent MAPK/AP-1 signaling, green arrows indicate activating or restorative immune effects, and red inhibitory lines indicate blockade or suppression of signaling. The balance symbol indicates the context-dependent modulation of regulatory T-cell function by OX40 signaling, which may either reduce suppressive activity or, under certain conditions, support Treg proliferation or survival. Therapeutic OX40 agonism enhances antitumor T-cell responses, whereas OX40/OX40L blockade reduces co-stimulation and may suppress pathological immune activation. Abbreviations: AKT, protein kinase B; APC, antigen-presenting cell; AP-1, activator protein 1; Bcl-2, B-cell lymphoma 2; Bcl-xL, B-cell lymphoma-extra large; CD3, cluster of differentiation 3; FOXP3, forkhead box P3; IFN-γ, interferon gamma; IL-2, interleukin 2; MAPK, mitogen-activated protein kinase; MHC, major histocompatibility complex; mTOR, mechanistic target of rapamycin; NF-κB, nuclear factor kappa B; OX40, tumor necrosis factor receptor superfamily member 4; OX40L, tumor necrosis factor ligand superfamily member 4; PI3K, phosphoinositide 3-kinase; TCR, T-cell receptor; TRAF, TNF receptor-associated factor; Treg, regulatory T cell. Created in BioRender. Mertowski, S. (2026) https://BioRender.com/5zo4a5t.

Binding of OX40L leads to OX40 oligomerization and recruitment of TRAF family adaptor proteins, primarily TRAF2, TRAF3, and TRAF5. Subsequently, activation of the canonical and non-canonical NF-κB pathways, as well as the PI3K/AKT and MAPK pathways, occurs. This signaling increases the expression of antiapoptotic proteins such as Bcl-2 and Bcl-xL, supports cell proliferation, and enhances the production of effector cytokines, including IL-2 and IFN-γ [123,124,125]. The requirement for receptor oligomerization also provides a structural explanation for the dependence of OX40 signaling on the geometry and membrane organization of receptor–ligand complexes. OX40 activation also influences the metabolic program of T lymphocytes. Through PI3K/AKT and mTOR signaling, it supports increased energy demand, enhances glycolysis and oxidative metabolism, and maintains effector functions during a prolonged immune response. Simultaneously, it promotes the formation of long-lived memory cells, increasing the durability of the response after the initial antigenic stimulation subsides [133,134]. The importance of the OX40/OX40L axis also extends to the regulation of Treg cells. OX40 is constitutively present on some of these cells, but the consequences of its activation depend on the biological context. Signaling can limit their suppressive functions, for example, by destabilizing FOXP3 expression, while simultaneously supporting their proliferation and survival under certain conditions. The ultimate effect depends on the phase of the response, the cell type present in the microenvironment, and the simultaneous activity of other costimulatory and coinhibitory pathways. The OX40/OX40L axis therefore serves a dual function: it enhances the expansion and survival of effector lymphocytes and can limit the local suppressive activity of Tregs. The mechanism of OX40-dependent signaling and its impact on survival, metabolism, memory cell differentiation, and Treg activity are presented in Figure 17 [130,131,132,133,134,135].

### 4.9. ICOS/ICOSL

The ICOS/ICOSL axis is a key costimulatory system regulating the adaptive immune response. ICOS is a receptor in the CD28 family, and its ligand, ICOSL (CD275, B7-H2), belongs to the B7 family. Unlike classical CD28-dependent costimulation, which plays a particularly important role in the early stages of T cell activation, ICOS signaling is primarily involved in maintaining and directing the response after antigen recognition. It influences the proliferation, survival, differentiation, and effector functions of T lymphocytes. Depending on the cell type and microenvironment, the ICOS/ICOSL axis can enhance antimicrobial and antitumor responses or support immune tolerance and immunosuppressive mechanisms [10,136,137,138,139]. ICOS is an inducible receptor whose expression increases following T cell activation. It is found primarily on activated CD4^+^ and CD8^+^ T cells, regulatory T cells, and follicular helper T cells. Its role in the differentiation and maintenance of Tfh cell function, which helps organize the humoral immune response, is particularly important. ICOS expression has also been reported on NK cells, macrophages, and selected dendritic cell subpopulations. ICOSL is broadly expressed on professional antigen-presenting cells, including B lymphocytes, dendritic cells, and macrophages. This ligand can also be present on endothelial and epithelial cells, selected tumor cells, and immunofibroblasts, which contribute to tertiary lymphatic structures [10,136,137,138,139].

The biological properties of the ICOS/ICOSL axis result from the characteristic structural organization of both proteins. ICOS is a type I transmembrane protein containing an extracellular IgV domain, a single transmembrane segment, and a short cytoplasmic tail that recruits intracellular signaling molecules. The extracellular IgV domain contains a defined ligand-binding surface, including the FG loop, where the FDPPPF motif contributes critically to ICOSL recognition; substitutions of key residues within this interface markedly reduce ICOS-ICOSL binding [135,136,137,138]. Its extracellular domain is stabilized by disulfide bonds and contains N-linked glycosylation sites essential for proper folding and ligand binding. Importantly, N-linked glycosylation at ICOS residue N110 directly participates in the receptor–ligand interface and can modulate binding affinity, demonstrating that a post-translational modification identified from sequence analysis may have a measurable effect on receptor–ligand interaction [136,137,138,139]. ICOSL is also a type I membrane protein, but its extracellular portion contains both an IgV domain and an IgC2 domain. This organization enables interaction with ICOS and supports the stability of the receptor–ligand complex.

**Figure 18:**
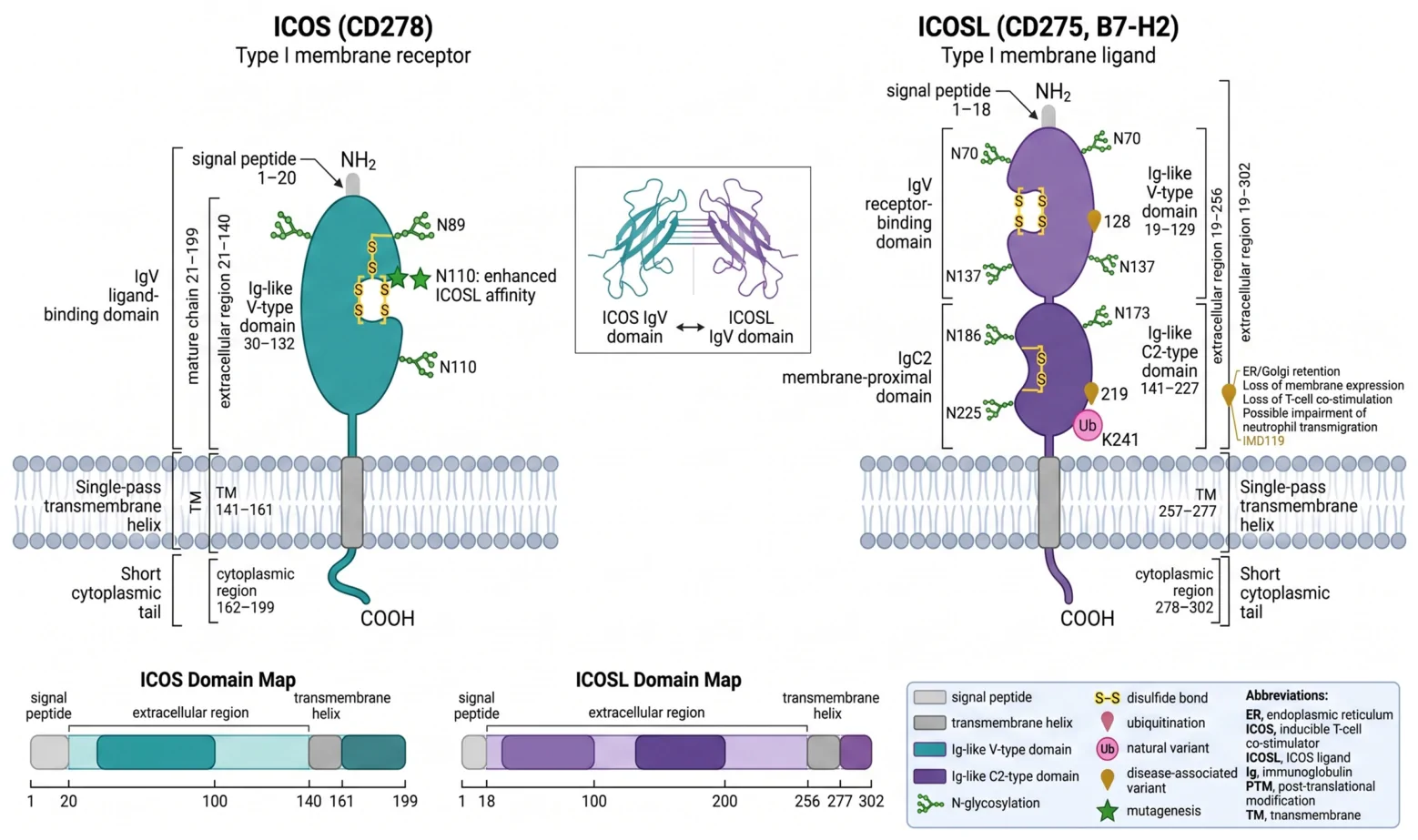
Structural and functional organization of the ICOS/ICOSL immune checkpoint axis [32,41]. The figure illustrates the domain architecture, membrane topology, post-translational modifications, and selected natural and disease-associated variants of ICOS and ICOSL. ICOS contains an extracellular Ig-like V-type ligand-binding domain, whereas ICOSL contains an Ig-like V-type receptor-binding domain and an Ig-like C2-type membrane-proximal domain. Yellow text highlights disease-associated functional consequences; IMD119 denotes immunodeficiency 119 associated with alterations in ICOSL, including impaired membrane expression and T-cell co-stimulation. Abbreviations: B7-H2, B7 homolog 2; CD275, cluster of differentiation 275; CD278, cluster of differentiation 278; COOH, carboxyl terminus (C-terminus); ER, endoplasmic reticulum; ICOS, inducible T-cell co-stimulator; ICOSL, ICOS ligand; Ig, immunoglobulin; IgC2, immunoglobulin-like C2-type domain; IgV, immunoglobulin-like V-type domain; IMD119, immunodeficiency 119; NH_2_, amino terminus (N-terminus); PTM, post-translational modification; TM, transmembrane; Ub, ubiquitination. Created in BioRender. Mertowski, S. (2026) https://BioRender.com/5zo4a5t.

Figure 18 presents an integrated characterization of the membrane topology, domain organization, posttranslational modifications, sequence variants, and functional regions of ICOS and ICOSL [32,41]. The described and predicted isoforms of both molecules are summarized in Supplementary Tables S17 and S18.

At the molecular level, ICOS comprises a signal peptide at positions 1–20, an extracellular domain at positions 21–140, a transmembrane segment at positions 141–161, and a cytoplasmic domain at positions 162–199. The IgV domain spans residues 30–132. The extracellular portion contains two disulfide bonds (42–109 and 63–83) and two N-glycosylation sites at positions 89 and 110. Among these modifications, N110 is of particular functional relevance because its glycan is positioned directly at the ICOS-ICOSL interface and can sterically restrict ligand binding; removal of this glycan has been experimentally shown to increase the apparent affinity of ICOS for ICOSL [138]. A mutation at position 110 has also been described, which increases ICOS affinity for ICOSL. ICOSL contains a signal peptide at positions 1–18, an extracellular domain spanning residues 19–256, a transmembrane segment at positions 257–277, and a short cytoplasmic tail at positions 278–302. The IgV domain spans positions 19–129, while the IgC2 domain spans positions 141–227. The extracellular portion contains disulfide bridges 37–113 and 158–216, as well as N-glycosylation sites at positions 70, 137, 173, 186, and 225. A ubiquitinated lysine residue has been described at position 241. The variant at position 219 is of particular functional significance, as it is associated with protein retention in the endoplasmic reticulum and Golgi apparatus, loss of membrane expression, and impaired T cell costimulation. This variant may also impair neutrophil transmigration across the endothelium. Upon binding ICOS to ICOSL, the PI3K/AKT pathway is activated, which supports the survival, proliferation, and differentiation of T lymphocytes. Compared to the CD28/CD80–CD86 axis, ICOS signaling induces IL-2 production to a lesser extent, but plays a more significant role in maintaining the effector response and shaping the functional profile of activated lymphocytes. This pathway participates in the regulation of Th1, Th2, and Th17 responses, but is particularly important for Tfh cells [32,41].

**Figure 19:**
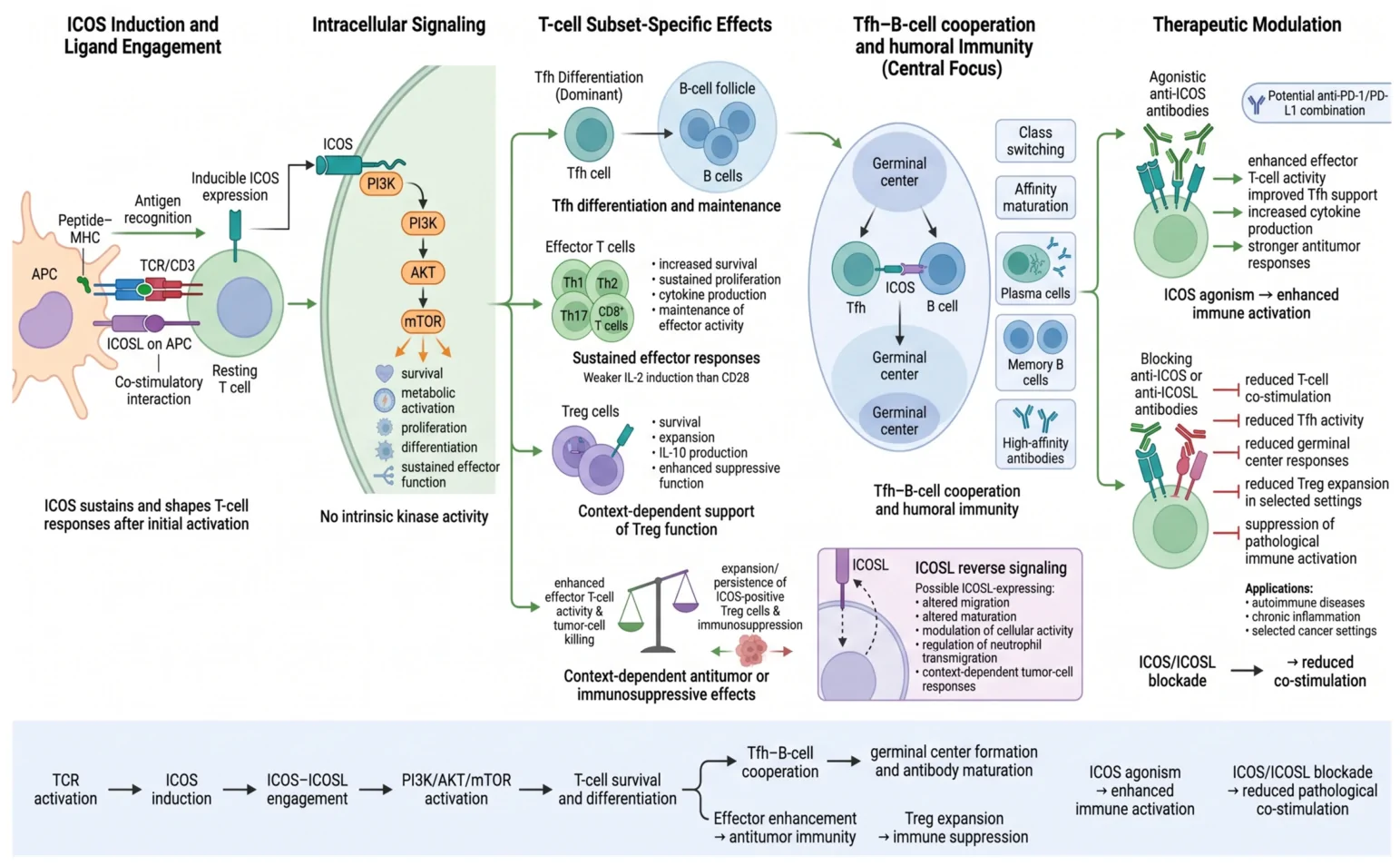
Mechanism of action and therapeutic modulation of the ICOS/ICOSL co-stimulatory immune checkpoint axis [136,137,138,139,140]. he figure illustrates ICOS induction and ligand engagement, PI3K/AKT/mTOR-dependent intracellular signaling, T-cell subset-specific effects, Tfh–B-cell cooperation and humoral immunity, and therapeutic modulation of the ICOS/ICOSL pathway. ICOS signaling supports Tfh differentiation, effector T-cell responses, germinal center formation, antibody maturation, and, in a context-dependent manner, Treg survival and expansion. The balance symbol represents the context-dependent balance between ICOS-mediated effector/antitumor activity and Treg-associated immunosuppressive effects; green represents enhanced effector T-cell activity and tumor-cell killing, whereas purple represents Treg-associated expansion, persistence, and immunosuppression. Green arrows indicate stimulatory, activating, or antitumor effects, whereas red arrows and inhibitory lines indicate immunosuppressive effects, blockade, or reduced co-stimulation. Therapeutically, ICOS agonism may enhance immune activation, whereas ICOS/ICOSL blockade may reduce pathological co-stimulation. Abbreviations: AKT, protein kinase B; APC, antigen-presenting cell; CD3, cluster of differentiation 3; CD8, cluster of differentiation 8; CD28, cluster of differentiation 28; ICOS, inducible T-cell co-stimulator; ICOSL, ICOS ligand; IL-2, interleukin 2; IL-10, interleukin 10; MHC, major histocompatibility complex; mTOR, mechanistic target of rapamycin; PD-1, programmed cell death protein 1; PD-L1, programmed death-ligand 1; PI3K, phosphoinositide 3-kinase; TCR, T-cell receptor; Tfh, follicular helper T cell; Th1, T helper 1 cell; Th2, T helper 2 cell; Th17, T helper 17 cell; Treg, regulatory T cell. Created in BioRender. Mertowski, S. (2026) https://BioRender.com/5zo4a5t.

Activity of the ICOS/ICOSL axis is essential for proper germinal center formation, supporting interactions between Tfh cells and B lymphocytes, immunoglobulin class switching, and antibody affinity maturation. As a result, ICOS signaling influences both the development of the cellular response and the quality and persistence of the humoral response. A significant aspect of ICOSL biology is its ability to transmit feedback signals to the cell that expresses the ligand. This reverse signaling can influence dendritic cell migration, maturation, and activity; osteoclast function; and the behavior of some cancer cells. This indicates that ICOSL does not merely function as a passive ligand but can actively participate in bidirectional communication between immune cells and the tissue microenvironment [136,137]. The ICOS/ICOSL axis therefore plays a complex regulatory role. On the one hand, it supports effector cell differentiation, the Tfh response, and humoral maturation; on the other, it can promote the expansion and maintenance of Treg cells. The ultimate effect of activation of this axis depends on the dominant cell population, the phase of the immune response, and the local microenvironmental conditions. The mechanism of ICOS signaling, its impact on effector cells, Tfh, Treg cells, and the humoral response, as well as the potential importance of ICOSL feedback signaling, is presented in Figure 19 [136,137,138,139,140].

### 4.10. 4-1BB/4-1BBL

**Figure 20:**
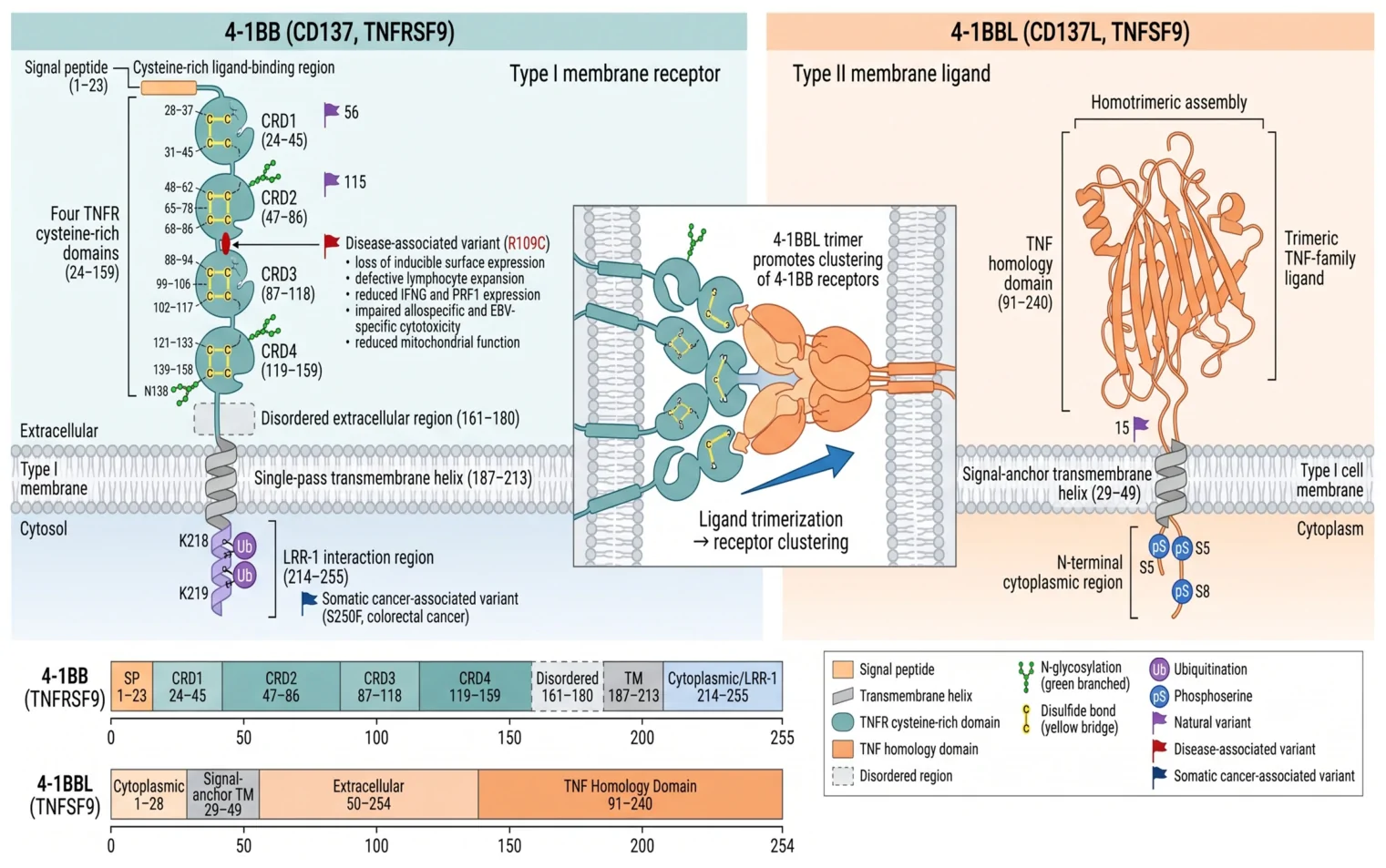
Structural and functional organization of the 4-1BB/4-1BBL immune checkpoint axis [34,43]. The figure illustrates the domain architecture, membrane topology, post-translational modifications, selected sequence variants, and ligand-induced clustering of 4-1BB and 4-1BBL. Trimeric 4-1BBL promotes clustering of 4-1BB receptors, facilitating receptor activation. Red text indicates a disease-associated variant; R190C denotes an arginine-to-cysteine substitution at residue 190 of 4-1BB/TNFRSF9. Abbreviations: 4-1BB, tumor necrosis factor receptor superfamily member 9; 4-1BBL, tumor necrosis factor ligand superfamily member 9; CD137, cluster of differentiation 137; CD137L, cluster of differentiation 137 ligand; CRD, cysteine-rich domain; IMD109, immunodeficiency 109; LRR-1, leucine-rich repeat protein 1; PRF1, perforin 1; PTM, post-translational modification; pS, phosphoserine; SP, signal peptide; THD, TNF homology domain; TM, transmembrane; TNFR, tumor necrosis factor receptor; TNFRSF9, tumor necrosis factor receptor superfamily member 9; TNFSF9, tumor necrosis factor ligand superfamily member 9; Ub, ubiquitination. Created in BioRender. Mertowski, S. (2026) https://BioRender.com/5zo4a5t comprises a signal peptide at positions 1–23, an extracellular domain at positions 24–186, a transmembrane segment at positions 187–213, and a cytoplasmic domain at positions 214–255. Four cysteine-rich regions are located at positions 24–45, 47–86, 87–118, and 119–159. Additionally, the extracellular portion contains a disordered region spanning residues 161–180, while the cytoplasmic domain interacts with LRR-1. Receptor stability is ensured by an extensive network of disulfide bonds and N-glycosylation at positions 138 and 149. Ubiquitination of residues K218 and K219 has been described in the cytoplasmic portion. Several natural variants have been identified in 4-1BB, including changes at positions 56, 115, and 176, as well as a somatic variant at position 250, described in a colon cancer specimen. Of particular functional importance is the variant at position 109 associated with IMD109. Its presence is associated with loss of detectable 4-1BB expression following stimulation of peripheral blood mononuclear cells, impaired lymphocyte expansion, reduced expression of IFNG and perforin, impaired allospecific and EBV-specific cytotoxicity, and impaired mitochondrial function. Thus, the TNFRSF9 variant affecting residue 109 provides a direct example of how a single sequence alteration can translate into loss of receptor expression and impaired downstream immune function, linking sequence variation with protein availability and cellular phenotype [142,143,144,145].

The 4-1BB/4-1BBL axis is one of the most important costimulatory systems in the tumor necrosis factor and its receptor (TNF/TNFR) superfamily. It regulates immune cell activation, survival, metabolism, and effector functions. The 4-1BB receptor (CD137, TNFRSF9) is an inducible costimulatory receptor, while its natural ligand, 4-1BBL (CD137L, TNFSF9), belongs to the TNF ligand family. 4-1BB signaling does not initiate primary T cell activation but rather amplifies and perpetuates the response following antigen recognition, increasing the proliferation, survival, and cytotoxic activity of T lymphocytes and NK cells [141,142,143]. 4-1BB expression is low on resting cells, but increases following activation of CD4^+^ and CD8^+^ T cells, reaching its highest level during the effector response phase. The receptor is also expressed on NK cells, monocytes, macrophages, dendritic cells, neutrophils, eosinophils, mast cells, and regulatory T cells. In the tumor microenvironment, 4-1BB expression increases on tumor-infiltrating lymphocytes, and hypoxia may modulate its levels. 4-1BBL ligand is expressed primarily on professional antigen-presenting cells, including dendritic cells, macrophages, and B lymphocytes, where it participates in the transmission of costimulatory signals that sustain T cell activity [141,142,143]. The biological properties of the 4-1BB/4-1BBL axis are closely related to the structural organization of both proteins. 4-1BB is a type I transmembrane protein containing a cysteine-rich extracellular region, a single transmembrane helix, and a short cytoplasmic tail responsible for the recruitment of adaptor proteins. The extracellular portion of the receptor contains four TNFR-Cys repeats, stabilized by multiple disulfide bonds, and N-linked glycosylation sites. The organization of these cysteine-rich domains contributes to the formation of the extracellular ligand-binding surface, while the spatial arrangement of the receptor is important for the clustering required for productive signaling [142,143,144,145]. 4-1BBL is a type II membrane protein whose C-terminal extracellular portion contains a TNF homology domain and forms a functional oligomeric complex capable of clustering 4-1BB receptors. The oligomeric organization of 4-1BBL is therefore functionally relevant because multivalent ligand engagement promotes receptor clustering and facilitates the assembly of the intracellular signaling complex [142,143,144,145]. Figure 20 presents an integrated characterization of the membrane topology, domain organization, posttranslational modifications, sequence variants, and functional regions of 4-1BB and 4-1BBL [34,43]. The described and predicted isoforms of both molecules are summarized in Supplementary Table S19.

4-1BBL contains a short cytoplasmic region at positions 1–28, a transmembrane segment acting as a signaling anchor at positions 29–49, and an extracellular domain spanning residues 50–254. The TNF homology domain is located at positions 91–240 and is responsible for ligand oligomerization and receptor binding. Serine phosphorylation at positions 5 and 8 has been described in the cytoplasmic fragment of 4-1BBL, while a natural variant recorded in dbSNP occurs at position 15. Binding of oligomeric 4-1BBL leads to 4-1BB clustering and recruitment of the adaptor proteins TNF receptor-associated factor 1 (TRAF1), TNF receptor-associated factor 2 (TRAF2), and TNF receptor-associated factor 3 (TRAF3). The proteins cIAP1 and cIAP2 also participate in the formation of a functional signaling complex. Receptor activation triggers the activation of the canonical and non-canonical NF-κB pathways, as well as the ERK and p38 MAPK pathways, which jointly regulate the transcription of genes involved in the survival, proliferation, and effector functions of immune cells [145,146,147,148]. The structural organization of 4-1BB is also directly relevant to therapeutic antibody recognition. Recent structural studies of agonistic anti-4-1BB antibodies have demonstrated that antibodies recognizing different extracellular regions of the receptor can produce distinct functional effects depending on epitope location and receptor cross-linking. For example, the agonistic antibody 1618 forms a defined complex with 4-1BB, whereas the tumor-targeted agonist ATOR-1017 recognizes an epitope spanning CRD2 and CRD3 that overlaps the 4-1BBL-binding region and blocks ligand binding [148]. These findings demonstrate that the extracellular domain organization of 4-1BB is not only relevant to physiological ligand recognition but can also determine antibody binding, receptor clustering, and the resulting agonistic activity [145]. In T lymphocytes, 4-1BB activation increases the expression of antiapoptotic proteins, including Bcl-xL and Bfl-1, limits apoptosis, and promotes long-term maintenance of the response. Simultaneously, it enhances the production of IFN-γ and IL-2, increases the cytotoxic activity of CD8^+^ lymphocytes, and supports their ability to eliminate target cells. The influence of this axis on cellular metabolism is particularly important. 4-1BB signaling increases mitochondrial biogenesis, oxidative phosphorylation capacity, and energy reserves, thereby promoting the development of long-lived memory lymphocytes and maintaining effector functions under conditions of chronic stimulation [148,149,150].

### 4.11. GITR/GITRL

The GITR/GITRL axis belongs to the costimulatory systems of the tumor necrosis factor and its receptor (TNF/TNFR) superfamily. It regulates the activation, survival, and effector functions of immune cells. GITR (CD357, TNFRSF18) is a costimulatory receptor constitutively highly expressed on regulatory T cells, while its expression increases on conventional CD4^+^ and CD8^+^ T cells upon activation. Depending on cell type, response phase, and microenvironmental conditions, GITR/GITRL signaling may enhance the antitumor response or participate in the regulation of immune tolerance [151,152,153,154]. GITR is also found on NK cells, B cells, NKT cells, macrophages, and granulocytes. Its transcripts have also been detected in selected non-hematopoietic tissues, including skin and lung. GITRL (TNFSF18) is expressed primarily on antigen-presenting cells, such as dendritic cells, macrophages, and activated B lymphocytes. The ligand has also been described on endothelial cells and tumor cells. GITRL can also occur in a soluble form, sGITRL, detected in the serum of patients with certain types of cancer [151,152,153,154].

**Figure 21:**
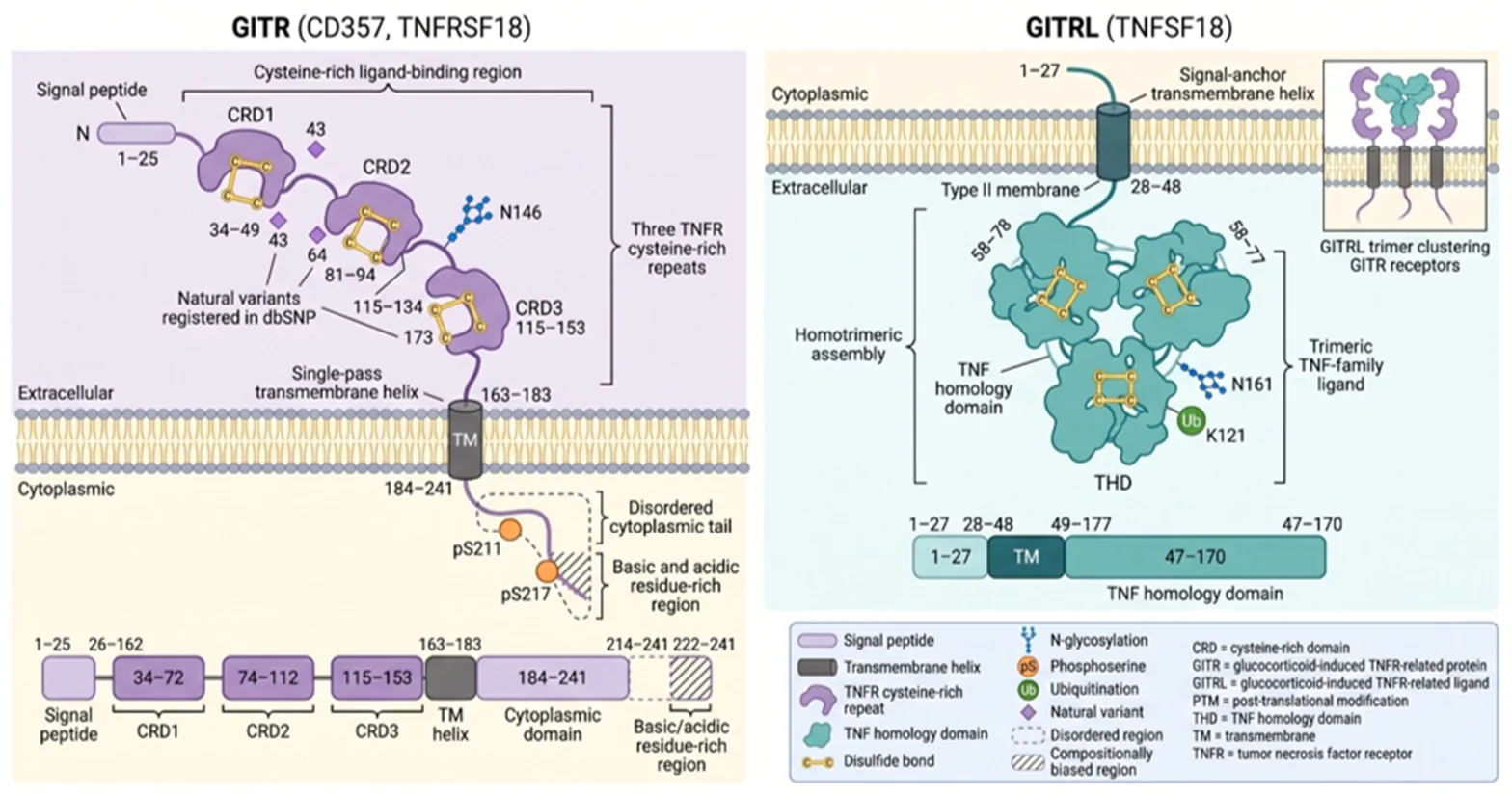
Structural and functional organization of the GITR/GITRL immune checkpoint axis [29,44]. The figure illustrates the domain architecture, membrane topology, post-translational modifications, natural variants, and oligomeric organization of GITR and GITRL. GITR contains three extracellular TNFR cysteine-rich domains and a cytoplasmic region, whereas GITRL forms a homotrimeric TNF-family ligand that promotes clustering of GITR receptors. Abbreviations: CD357, cluster of differentiation 357; CRD, cysteine-rich domain; dbSNP, Single Nucleotide Polymorphism Database; GITR, glucocorticoid-induced TNFR-related protein; GITRL, glucocorticoid-induced TNFR-related ligand; N-glycosylation, N-linked glycosylation; pS, phosphoserine; PTM, post-translational modification; THD, TNF homology domain; TM, transmembrane; TNFR, tumor necrosis factor receptor; TNFRSF18, tumor necrosis factor receptor superfamily member 18; TNFSF18, tumor necrosis factor ligand superfamily member 18; Ub, ubiquitination. Created in BioRender. Mertowski, S. (2026) https://BioRender.com/5zo4a5t.

The biological properties of the GITR/GITRL axis are closely related to the structural organization of both proteins. GITR is a type I transmembrane protein containing an extracellular cysteine-rich region, a single transmembrane segment, and a cytoplasmic signaling tail. The extracellular portion contains three TNFR-Cys repeats stabilized by numerous disulfide bonds. Structural studies have shown that the GITR–GITRL interface is unusually small and is formed predominantly by the B1 module of the second cysteine-rich domain (CRD2) of GITR. Within this interface, residues F106 and G109 are critical for ligand binding, and their substitution markedly reduces GITRL-induced signaling [152,153,154]. GITRL is a type II membrane protein whose C-terminal extracellular domain contains a TNF homology domain responsible for oligomerization and receptor binding. Importantly, GITR–GITRL recognition differs from the canonical interaction mode observed for many TNFR/TNFSF members, indicating that the precise domain architecture and amino acid composition of the receptor–ligand interface determine binding specificity and affinity [152,153,154]. An integrated characterization of the membrane topology, domain organization, post-translational modifications, sequence variants, and functional regions of GITR and GITRL is presented in Figure 21 [29,44]. The described and predicted isoforms of both molecules are summarized in Supplementary Table S20.

GITR comprises a signal peptide at positions 1–25, an extracellular domain at positions 26–162, a transmembrane segment at positions 163–183, and a cytoplasmic domain at positions 184–241. Three cysteine-rich regions span residues 34–72, 74–112, and 115–153. Stability of the extracellular portion is provided by disulfide bridges 34–49, 74–86, 81–94, 115–134, and 128–153, and additional structural regulation may be provided by N-glycosylation of residue N146. Phosphorylation of serines S211 and S217 has been described in the cytoplasmic tail. The terminal portion of the cytoplasmic domain, spanning residues 214–241, is disordered and contains a region enriched in both basic and acidic residues. Natural variants have been identified in GITR at positions 43, 64, 83, and 173 and are recorded in the dbSNP database. Their functional significance has not been fully elucidated and should therefore be presented as elements of sequence variation, without assigning unambiguous biological consequences. The structural data also demonstrate that sequence variation within the extracellular region can affect recombinant protein behavior: removal of an unpaired cysteine (C57S in human GITR) markedly improves protein expression, illustrating how individual residues and disulfide architecture can influence receptor stability and experimental production. GITRL contains a short cytoplasmic region spanning residues 1–27, a transmembrane segment that serves as a signaling anchor at positions 28–48, and an extracellular domain spanning residues 49–177. The TNF homology domain spans residues 47–170 and forms the active ligand complex and interacts with GITR. The extracellular portion contains a disulfide bond (58–78) and N-glycosylation sites at N129 and N161. Ubiquitination has also been described at position K121. Structural studies indicate that GITRL-mediated receptor activation depends not only on ligand binding but also on higher-order receptor organization at the cell surface. Human GITR–GITRL complexes can assemble into higher-order membrane networks, and disruption of the receptor–receptor interface reduces signaling and ligand-induced receptor organization [152,153,154]. Binding of oligomeric GITRL leads to GITR clustering and recruitment of TRAF family adaptor proteins. This results in activation of the NF-κB and MAPK pathways, which enhance the survival, proliferation, and effector functions of T lymphocytes. In effector cells, GITR signaling enhances the production of IL-2 and IFN-γ, increases the expression of antiapoptotic proteins, including Bcl-xL, and supports the development of an antitumor response [151,152,153,154].

A key element of GITR biology is its influence on the balance between effector lymphocytes and Tregs. In experimental models, activation of the receptor can weaken the suppressive properties of Tregs or increase the resistance of effector lymphocytes to their effects. However, studies using human cells indicate that this effect is not clear-cut and depends on signal intensity, the stage of cell activation, and the local microenvironment. GITR signaling can therefore simultaneously support the expansion of effector cells and modulate the function of regulatory populations [154,155,156].

GITRL need not act solely as a passive ligand. The possibility of feedback signals to GITRL-expressing cells has been described, which can influence the activation and maturation of antigen-presenting cells, cytokine production, and the properties of some tumor cells. However, the significance of this mechanism remains dependent on the cell type and biological context [156,157,158]. The role of the GITR/GITRL axis is particularly important in the tumor microenvironment. High GITR expression on Treg cells and the presence of GITRL on antigen-presenting cells and tumor cells influence the balance between effector response and immunosuppression. Activation of this pathway can increase the activity of T cells and NK cells and improve the ratio of effector cells to Treg cells within the tumor. However, the final effect depends on the dominant cell population and the intensity of the local regulatory response [156,157,158]. The structural organization of GITR is also directly relevant to therapeutic antibody recognition and activity. Structural and biophysical studies of agonistic anti-GITR antibodies have shown that antibody-mediated receptor clustering can mimic the organization induced by GITRL. In particular, bivalent antibodies such as DTA-1 promote GITR clustering and activate signaling, whereas monovalent Fab fragments lack this activity, demonstrating that antibody valency and the resulting receptor geometry are critical determinants of agonistic function [153,154,155,156,157,158]. Similarly, the ligand-blocking antibody IBI37G5 recognizes an epitope that substantially overlaps the GITRL-binding surface of CRD2 and can simultaneously achieve high-affinity binding and receptor agonism through appropriate receptor spacing.

### 4.12. CD27/CD70

The CD27/CD70 axis is a key costimulatory system in the tumor necrosis factor and its receptor superfamily. It regulates the activation, differentiation, survival, and development of immunological memory of immune cells. CD27 (TNFRSF7) is a costimulatory receptor that enhances lymphocyte responses following antigen recognition, while its ligand, CD70 (TNFSF7), is characterized by tightly controlled and usually transient expression. Under physiological conditions, activation of the CD27/CD70 axis supports the development of effector and memory cell responses, while its chronic activation can lead to impaired immune homeostasis, increased immunosuppression, and cancer progression [159,160,161,162].

CD27 is constitutively expressed on most CD4^+^ and CD8^+^ T cells, B lymphocytes, and NK cells. Particularly high levels of the receptor are observed on selected subpopulations of regulatory T cells. The highest expression of CD27 is found in lymphoid organs, including lymph nodes, spleen, thymus, and tonsils. CD70 appears primarily transiently on activated dendritic cells, B cells, and T cells. However, its aberrant, persistent expression has been described in numerous cancers, including renal cell carcinoma, nasopharyngeal carcinoma, gliomas, and lymphomas. A soluble form of CD70, sCD70, has also been detected, and its biological significance and potential as a biomarker remain under investigation [159,160,161,162].

**Figure 22:**
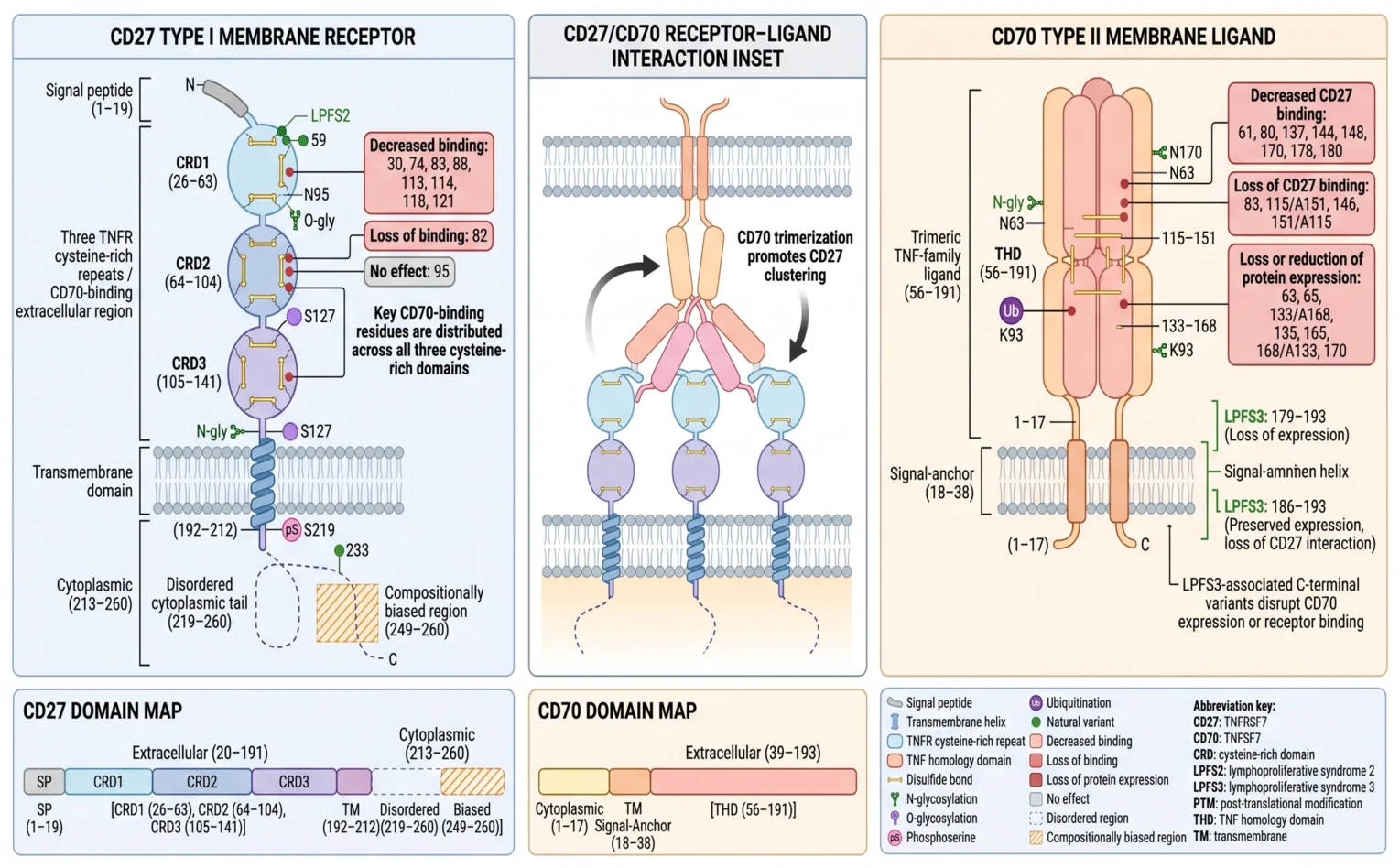
Structural and functional organization of the CD27/CD70 immune checkpoint axis [35,45]. The figure illustrates the domain architecture, membrane topology, post-translational modifications, selected sequence variants, and receptor–ligand organization of CD27 and CD70. Trimerization of CD70 promotes clustering of CD27 receptors. Green text highlights disease-associated alterations linked to lymphoproliferative syndromes, including LPFS2 and LPFS3. Dashed outlines indicate intrinsically disordered protein regions. Abbreviations: CD27, tumor necrosis factor receptor superfamily member 7; CD70, tumor necrosis factor ligand superfamily member 7; CRD, cysteine-rich domain; LPFS2, lymphoproliferative syndrome 2; LPFS3, lymphoproliferative syndrome 3; N-gly, N-linked glycosylation; O-gly, O-linked glycosylation; pS, phosphoserine; PTM, post-translational modification; SP, signal peptide; THD, TNF homology domain; TM, transmembrane; TNFR, tumor necrosis factor receptor; TNFRSF7, tumor necrosis factor receptor superfamily member 7; TNFSF7, tumor necrosis factor ligand superfamily member 7; Ub, ubiquitination. Created in BioRender. Mertowski, S. (2026) https://BioRender.com/5zo4a5t.

The functional properties of the CD27/CD70 axis are closely related to the structural organization of both proteins. CD27 is a type I transmembrane protein containing an extracellular cysteine-rich region, a single transmembrane segment, and a short cytoplasmic tail that recruits adaptor proteins. The extracellular portion comprises three TNFR-Cys repeats, stabilized by numerous disulfide bonds. CD70 is a type II membrane protein whose C-terminal extracellular portion contains a TNF homology domain, which is responsible for ligand oligomerization and CD27 binding. Structural analysis of the human CD27/CD70 complex demonstrated that CD70 forms a homotrimer that engages three CD27 molecules in a 3:3 stoichiometry, with the principal receptor–ligand contacts involving CRD2 and CRD3 of CD27 [163,164,165]. This organization is functionally relevant because multivalent CD70 engagement promotes receptor clustering, whereas the spatial presentation of CD70 on a surface substantially increases its ability to stimulate T cells compared with soluble ligand, indicating that ligand valency and membrane organization can influence the magnitude of the measured biological response [163,164,165]. An integrated characterization of the membrane topology, domain organization, post-translational modifications, sequence variants, and functional regions of CD27 and CD70 is presented in Figure 22 [35,45]. The described and predicted isoforms of both molecules are summarized in Supplementary Tables S21 and S22.

CD27 contains a signal peptide spanning residues 1–19, an extracellular domain spanning residues 20–191, a transmembrane segment spanning residues 192–212, and a cytoplasmic domain spanning residues 213–260. Three cysteine-rich regions are located at positions 26–63, 64–104, and 105–141, respectively. Their stability is ensured by disulfide bridges 27–39, 40–53, 43–62, 65–81, 84–96, 87–104, 106–120, and 112–117. In the extracellular moiety, N-glycosylation of residue N95 and O-glycosylation of S127 occur, whereas in the cytoplasmic moiety, phosphorylation of S219 has been described. The terminal portion of the cytoplasmic tail, encompassing residues 219–260, is disordered, while the region 249–260 is enriched in basic and acidic residues.

Mutagenesis studies indicate that numerous residues in the extracellular portion of CD27 participate in CD70 binding. Changes at positions 30, 74, 83, 88, 113, 114, 118, and 121 reduce ligand binding affinity, while a mutation at position 82 leads to loss of binding. A change at position 95 has no significant effect. These findings provide a direct structure–function relationship between individual residues within the extracellular domains of CD27 and receptor–ligand affinity, demonstrating that sequence-level information can be used to identify residues that are functionally relevant for CD70 recognition. Natural variants at positions 53, 59, and 233 have also been described within CD27. The variant at position 53 is associated with LPFS2, indicating the functional importance of the correct receptor structure. Thus, disease-associated sequence variation in the extracellular region may affect CD27 function by altering the structural integrity of the receptor or its ability to engage CD70, although the precise molecular consequences should be distinguished from variants for which only an association has been reported [163,164,165,166].

CD70 comprises a short cytoplasmic region at positions 1–17, a transmembrane segment acting as a signaling anchor at positions 18–38, and an extracellular domain at positions 39–193. The TNF homology domain spans residues 56–191 and is responsible for the formation of the active ligand structure and CD27 binding. CD70 is N-glycosylated at positions N63 and N170, contains a ubiquitinated K93 lysine, and disulfide bonds at positions 115–151 and 133–168. The structural analysis further showed that the CD70 disulfide-bonding pattern contributes to the integrity of the trimeric ligand, while alterations or deletions affecting the C-terminal region can disrupt the trimer core and result in loss of protein expression. Mutagenesis analyses of CD70 have shown that its interaction with CD27 depends on numerous residues distributed throughout the TNF homology domain. Changes at positions 61, 80, 137, 144, 148, 170, 178, and 180 reduce receptor binding, while mutations at positions 83, 115, 146, and 151 can lead to receptor loss. Some changes, including those at positions 63, 65, 133, 135, 165, and 168, primarily affect protein expression or stability. Variants involving residues 179–193 and 186–193, associated with LPFS3, lead to loss of protein expression or impaired interaction with CD27, respectively. These observations illustrate that sequence variants can influence CD70 at two distinct levels: directly by altering the receptor-binding interface and indirectly by destabilizing the trimeric ligand and reducing its cellular expression [164,165,166,167]. Binding of oligomeric CD70 leads to CD27 clustering and recruitment of the adaptor proteins TRAF2 and TRAF5. This results in activation of the NF-κB and MAPK pathways, including JNK kinase, which collectively enhance the proliferation, survival, and effector functions of T lymphocytes. CD27 signaling enhances the Th1 response, increases the activity of cytotoxic CD8^+^ lymphocytes, and increases IFN-γ production [165].

The CD27/CD70 axis also plays a crucial role in the development and maintenance of immunological memory. It supports the survival of activated T lymphocytes, limits their elimination after the expansion phase, and promotes the differentiation of memory cells. This effect is particularly important in antiviral, antitumor, and post-vaccination responses [165,166,167].

However, chronic activation of the CD27/CD70 axis can lead to different consequences. Prolonged expression of CD70 in the tumor microenvironment can disrupt the balance between effector and regulatory responses, promote lymphocyte exhaustion, and expand immunosuppressive cells. Accumulating evidence suggests that CD27 activation can reprogram Treg cell metabolism by enhancing oxidative phosphorylation and altering cholesterol homeostasis, thereby increasing survival and suppressive function [165,166,167]. The CD27/CD70 axis therefore plays a time- and context-dependent role. Short-term activation supports effective cellular responses and memory development, while chronic signaling can promote lymphocyte dysfunction, immunosuppression, and disease progression. The structural organization of CD27 is also directly relevant to therapeutic antibody recognition and agonistic activity. Recent structural and functional studies showed that agonistic anti-CD27 antibodies recognize distinct regions of the extracellular receptor and that their activity depends not only on epitope location but also on receptor clustering and Fc-mediated cross-linking. In particular, antibodies recognizing CRD2/CRD3 can interfere with CD70 binding, whereas antibodies targeting other regions can inhibit or modulate CD27 signaling through steric effects without directly competing with CD70 [166,167]. These findings demonstrate that the extracellular domain architecture of CD27 can determine antibody epitope accessibility and, together with antibody valency and Fcγ receptor engagement, influence the magnitude and direction of the functional response [164,165,166,167].

### 4.13. CD40/CD40L

The CD40/CD40L axis (CD154) is a key costimulatory system of the tumor necrosis factor and its receptor (TNF/TNFR) superfamily, integrating humoral and cellular responses. The interaction of CD40 with CD40L plays a fundamental role in B cell activation, the maturation of antigen-presenting cells, the regulation of T cell responses, and the development of immunological memory. Due to its broad influence on the activity of numerous cell populations, this axis is an important target for both anticancer immunotherapy and the treatment of autoimmune diseases and chronic inflammatory conditions [168,169,170,171].

CD40 is a receptor belonging to the TNFR superfamily, constitutively expressed primarily on B lymphocytes, dendritic cells, monocytes, and macrophages. Its presence has also been demonstrated on numerous non-hematopoietic cells, including endothelial cells, fibroblasts, epithelial cells, and vascular smooth muscle cells. CD40L (CD154, TNFSF5) is expressed primarily on activated CD4^+^ T cells, but is also found on activated platelets, B cells, monocytes, and NK cells. Proteolytic cleavage of the extracellular portion produces a soluble form of CD40L, sCD40L, which retains biological activity and participates in the regulation of inflammatory processes and communication between immune and endothelial cells [168,169,170,171].

The biological properties of the CD40/CD40L axis are closely linked to the structural organization of both proteins. CD40 is a type I transmembrane protein containing an extracellular cysteine-rich region, a single transmembrane segment, and a cytoplasmic signaling tail. The extracellular portion contains four TNFR-Cys repeats, stabilized by numerous disulfide bridges. The organization of the cysteine-rich domains directly contributes to CD40L recognition, with the receptor engaging a crevice formed between two CD40L subunits; therefore, the three-dimensional arrangement of the extracellular domains, rather than domain number alone, determines productive ligand binding. CD40L is a type II membrane protein whose C-terminal extracellular portion contains a TNF homology domain responsible for oligomerization, CD40 binding, and interactions with selected integrins. The trimeric organization of CD40L is functionally important because it enables multivalent engagement and clustering of CD40 receptors, which is required for efficient downstream signaling [170,171,172].

**Figure 23:**
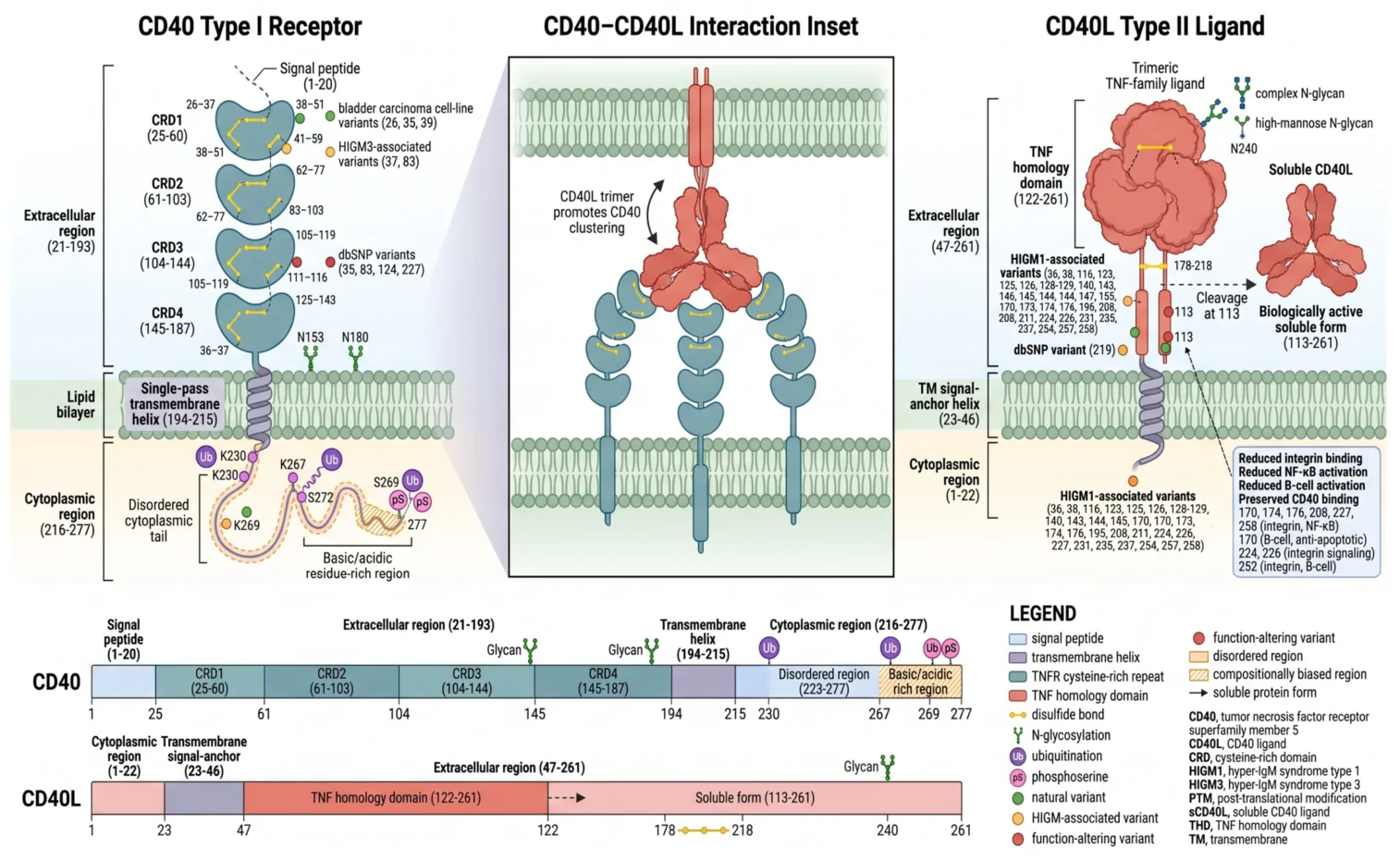
Structural and functional organization of the CD40/CD40L immune checkpoint axis [36,46]. This schematic summarizes the structural features, membrane organization, post-translational modifications, selected sequence variants, and interaction between CD40 and CD40L. Trimeric CD40L promotes CD40 receptor clustering, whereas dashed arrows denote proteolytic processing of membrane-bound CD40L leading to the generation of biologically active soluble CD40L (sCD40L). Abbreviations: CD40, tumor necrosis factor receptor superfamily member 5; CD40L, CD40 ligand; CRD, cysteine-rich domain; HIGM1, hyper-IgM syndrome type 1; HIGM3, hyper-IgM syndrome type 3; NF-κB, nuclear factor kappa B; N-glycan, N-linked glycan; PTM, post-translational modification; sCD40L, soluble CD40 ligand; SP, signal peptide; THD, TNF homology domain; TM, transmembrane; TNFR, tumor necrosis factor receptor; TNFRSF5, tumor necrosis factor receptor superfamily member 5; TNFSF5, tumor necrosis factor ligand superfamily member 5; Ub, ubiquitination. Created in BioRender. Mertowski, S. (2026) https://BioRender.com/5zo4a5t.

An integrated characterization of the membrane topology, domain organization, post-translational modifications, sequence variants, and functional regions of CD40 and CD40L is presented in Figure 23 [36,46]. The described and predicted isoforms of both molecules are summarized in Supplementary Table S23.

CD40 contains a signal peptide spanning residues 1–20, an extracellular domain spanning residues 21–193, a transmembrane segment spanning residues 194–215, and a cytoplasmic domain spanning residues 216–277. Four cysteine-rich regions are located at positions 25–60, 61–103, 104–144, and 145–187, respectively. Their correct conformation is maintained by disulfide bridges 26–37, 38–51, 41–59, 62–77, 83–103, 105–119, 111–116, and 125–143. Additional structural regulation is provided by N-glycosylation of residues N153 and N180. In the cytoplasmic tail, ubiquitination of K230 and K267 and phosphorylation of S269 and S272 have been described. The terminal fragment of the receptor, encompassing residues 223–277, is disordered, and the region 264–277 is enriched in basic and acidic residues. Natural variants of CD40 have been described at positions 26, 35, 37, 39, 83, 124, and 227. Some of these variants were identified in the Hu549 bladder cancer cell line, while variants at positions 37 and 83 are associated with hyper-IgM type 3 syndrome. These variants illustrate that preservation of the extracellular disulfide-bonded architecture is relevant not only to receptor structure but also to CD40 expression and ligand-dependent function; however, sequence variants without demonstrated functional effects should be distinguished from disease-associated mutations with experimentally established consequences. More recent mechanistic evidence further shows that the mechanical properties of the CD40–CD40L interaction contribute to signaling: CD40 forms a force-dependent catch bond with CD40L, and X-linked hyper-IgM-associated CD40L mutations can impair catch-bond formation and force-enhanced CD40 signaling, providing a direct link between sequence variation, receptor–ligand mechanics, and immune function. CD40L contains a short cytoplasmic region spanning residues 1–22, a transmembrane segment acting as a signaling anchor at positions 23–46, and an extracellular domain spanning residues 47–261. The TNF homology domain is located at positions 122–261. The membrane-bound form of CD40L encompasses the full sequence 1–261, while the soluble form corresponds to fragments 113–261. The ligand structure contains a disulfide bridge spanning residues 178–218 and an alternatively glycosylated residue, N240, which may carry complex or high-mannose N-glycan moieties. Numerous variants of CD40L associated with hyper-IgM syndrome type 1 have been described. These variants encompass both the transmembrane region and the TNF homology domain responsible for CD40 binding. Of particular functional significance are changes at positions 170, 174, 176, 208, 227, and 258, which reduce the interaction between the soluble form of CD40L and the integrins α5β1 and αVβ3 and impair NF-κB activation. The variant at position 170 may further limit B cell activation, anti-apoptotic signaling, and CD40 binding. Changes at positions 224 and 226, however, may not impair integrin binding but affect the intensity of NF-κB activation. Mutagenesis studies have also shown that simultaneous changes at residues 224 and 226 reduce α5β1 integrin binding, B cell activation, NF-κB activation, and anti-apoptotic signaling, without significantly affecting CD40 binding by the soluble form. A mutation at position 252 leads to a similar reduction in integrin-dependent functions, also without a clear disruption in interaction with CD40. These observations demonstrate that the CD40L extracellular domain contains partially separable functional surfaces, so that sequence alterations can selectively affect CD40 binding, integrin recognition, or downstream signaling rather than producing a uniform loss of ligand activity [170,171,172].

Binding of oligomeric CD40L leads to CD40 clustering and recruitment of TRAF family adaptor proteins, primarily TRAF2, TRAF3, TRAF5, and TRAF6. Consequently, the canonical and non-canonical NF-κB pathways, MAPK, and PI3K/AKT pathways are activated. Thus, ligand oligomerization and receptor clustering represent an important structural-to-functional transition: changes in the valency or spatial organization of CD40L can alter the efficiency of receptor activation and consequently the magnitude of downstream signaling. The ultimate effect depends on the cell type, its activation state, and the local microenvironment [168,169,170,171,172].

In B lymphocytes, CD40 signaling is essential for their proliferation, survival, and differentiation. It supports the formation of germinal centers, immunoglobulin class switching, antibody affinity maturation, and the development of plasma cells and memory B lymphocytes. Disturbances in the CD40/CD40L axis lead to profound impairments in the humoral response, as clinically exemplified by hyper-IgM syndromes associated with CD40 or CD40L defects. Activation of CD40 on dendritic cells leads to their maturation and functional “licensing.” This process involves increased expression of CD80, CD86, and MHC molecules, enhanced production of IL-12 and TNF-α, and improved ability to activate T lymphocytes. In this way, CD40 signaling enables effective coupling of the T helper cell response with the activation of cytotoxic lymphocytes and the humoral response [172,173,174,175]. The CD40/CD40L axis also participates in the activation of monocytes, macrophages, and endothelial cells, increasing the production of cytokines, chemokines, adhesion molecules, and prothrombotic factors. Activated platelet CD40L and its soluble form are of particular importance, as they can enhance vascular inflammation and interactions between platelets, leukocytes, and endothelium. These mechanisms highlight the importance of the axis not only in adaptive immunity but also in chronic inflammatory processes and immunothrombosis [172,173,174,175].

### 4.14. CD2/CD58

The CD2/CD58 axis is a key system that stabilizes contact between immune cells and amplifies the signals required for full T cell activation. Unlike classical inhibitory or costimulatory checkpoints, it primarily functions to organize and maintain the immunological synapse and to increase the efficiency of target cell recognition. Therefore, the CD2/CD58 axis plays a crucial role in both the natural antitumor response and the efficacy of modern cell therapies [176,177,178,179]. CD2 is a glycoprotein belonging to the immunoglobulin superfamily, expressed primarily on T cells and natural killer cells. Its expression increases following cell activation, with the highest levels typically observed on CD8^+^ T cells. Under conditions of chronic antigenic stimulation and progressive functional exhaustion of T lymphocytes, CD2 levels may decrease, which limits the stability of contact with the target cell and weakens the antitumor response. CD58, also known as LFA-3, is expressed on antigen-presenting cells, numerous non-hematopoietic cells, and many types of tumor cells. Loss or reduction of CD58 expression is one mechanism of immune escape in tumors [14,176,177,178,179].

**Figure 24:**
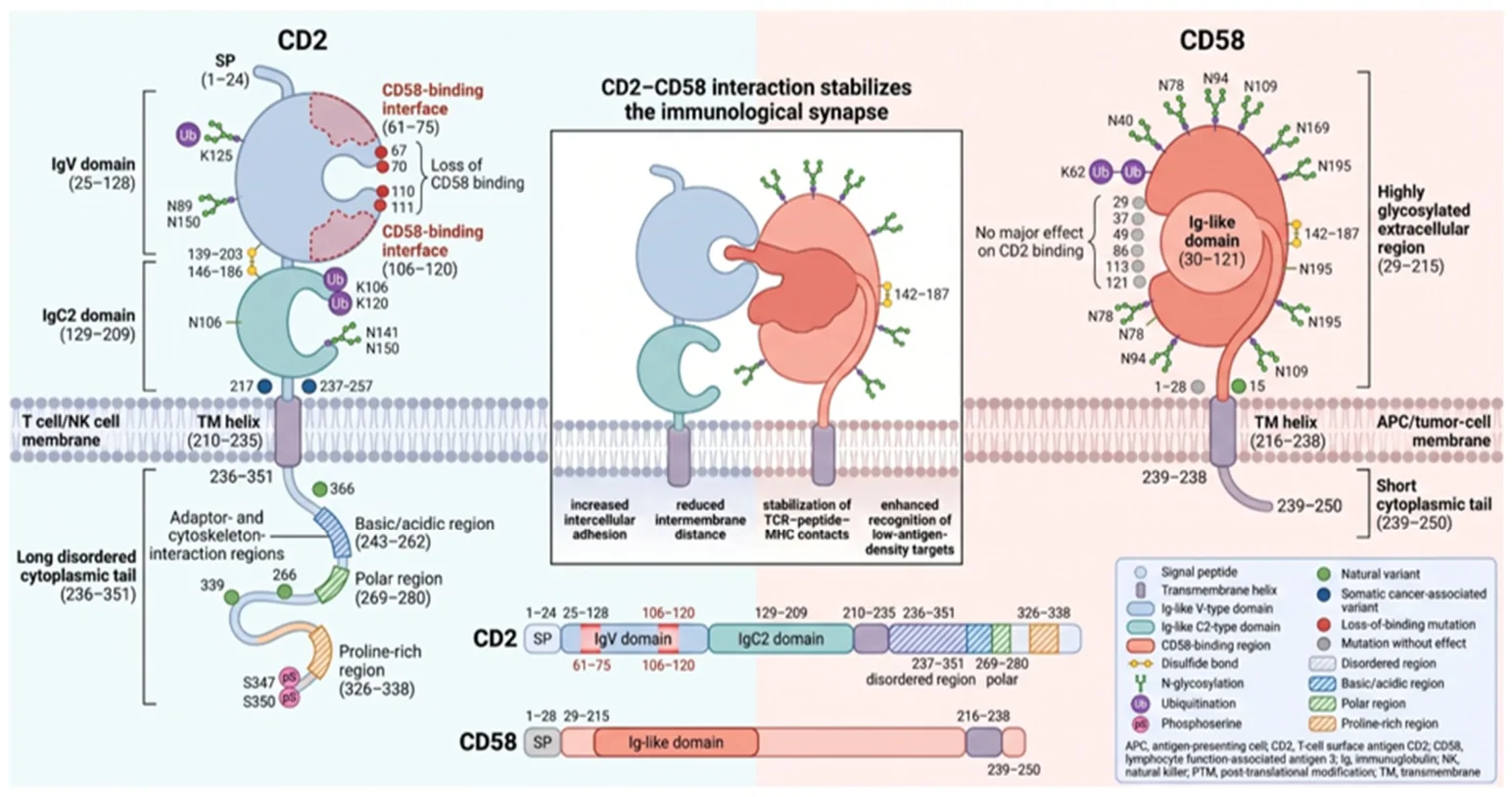
Structural and functional organization of the CD2/CD58 immune checkpoint axis [30,47]. The structural organization of CD2 and CD58 is presented together with their membrane topology, post-translational modifications, selected sequence variants, and receptor–ligand interaction. CD2–CD58 binding contributes to stabilization of the immunological synapse, increased intercellular adhesion, and stabilization of TCR–peptide–MHC contacts. Red text highlights the CD58-binding interfaces within the CD2 IgV domain, whereas red dashed borders delineate the corresponding CD58-binding regions. Abbreviations: APC, antigen-presenting cell; CD2, T-cell surface antigen CD2; CD58, lymphocyte function-associated antigen 3; IgC2, immunoglobulin-like C2-type domain; IgV, immunoglobulin-like V-type domain; MHC, major histocompatibility complex; NK, natural killer; PTM, post-translational modification; SP, signal peptide; TCR, T-cell receptor; TM, transmembrane; Ub, ubiquitination. Created in BioRender. Mertowski, S. (2026) https://BioRender.com/5zo4a5t.

The biological properties of the CD2/CD58 axis are closely related to the structural organization of both proteins. CD2 is a type I transmembrane protein containing two extracellular immunoglobulin superfamily domains, a single transmembrane segment, and a long cytoplasmic tail. The distal IgV-like domain directly participates in CD58 binding, while the proximal IgC2-like domain stabilizes the receptor’s position at the cell membrane. CD58 is also a type I membrane protein whose extracellular portion contains an immunoglobulin-like domain that binds CD2. The CD2–CD58 interaction is characterized by relatively modest affinity at the level of individual receptor–ligand pairs, but its rapid association and dissociation kinetics enable repeated binding and make the interaction particularly suitable for the dynamic reorganization of the immunological synapse [179,180]. Structural and mutagenesis studies identified a discrete binding hotspot within the CD2–CD58 interface; in particular, substitution of CD2 Tyr86 markedly reduces CD58 binding, demonstrating that individual residues within the IgV domain can make disproportionate contributions to overall receptor–ligand affinity [179,180,181]. An integrated characterization of the membrane topology, domain organization, post-translational modifications, sequence variants, and functional regions of CD2 and CD58 is presented in Figure 24 [30,47]. The described and predicted isoforms of both molecules are summarized in Supplementary Tables S24 and S25.

CD2 contains a signal peptide spanning residues 1–24, an extracellular domain spanning residues 25–209, a transmembrane segment spanning residues 210–235, and a cytoplasmic domain spanning residues 236–351. The IgV domain spans positions 25–128, and the IgC2 domain spans positions 129–209. Within the IgV domain, two regions essential for CD58 binding have been identified: one at positions 61–75 and the other at positions 106–120. Mutagenesis studies have shown that residues 67 and 70 are essential for CD58 binding, whereas changes at positions 110 and 111 result in the loss of binding to both CD58 and CD59. These observations indicate that the ligand-binding surface is conformationally organized across several regions of the IgV domain, meaning that domain integrity may be more informative for predicting CD58 recognition than the presence of any single linear sequence motif. Within CD2, a somatic variant at position 217, detected in breast cancer material, and natural variants at positions 266 and 339 have also been described. The extracellular portion of CD2 contains N-glycosylation sites at positions N89, N141, and N150, as well as disulfide bonds at positions 139–203 and 146–186. Ubiquitination has been reported at K106, K120, and K125, while phosphorylation at S347 and S350 has been reported in the terminal part of the cytoplasmic tail. The cytoplasmic domain, encompassing residues 237–351, is largely disordered and contains regions enriched in basic, acidic, polar, and proline residues, which favor interactions with adaptor proteins and cytoskeletal elements. Importantly, glycosylation of CD2 can modify the conformation of the extracellular binding interface and stabilize the CD2–CD58 complex, providing a direct example of how a posttranslational modification can influence receptor–ligand adhesion rather than merely protein maturation. CD58 contains a signal peptide at positions 1–28, an extracellular domain spanning residues 29–215, a transmembrane segment spanning residues 216–238, and a short cytoplasmic tail spanning residues 239–250. The immunoglobulin-like domain is located at positions 30–121 and mediates interaction with CD2. The extracellular portion contains numerous N-linked glycosylation sites at positions N40, N78, N94, N109, N169, and N195, as well as a disulfide bond at positions 142–187. Ubiquitination has been described at positions K62 and K78. Mutagenesis analyses of CD58 revealed that changes at positions 29, 37, 49, 86, 113, and 121 did not significantly affect CD2 binding. This suggests that the key interaction interface is diffuse and may depend more on maintaining the correct domain conformation than on individual residues. A natural variant at position 15 has also been described within CD58 [30,47]. Thus, the available evidence suggests that structural integrity and posttranslational modification of the extracellular domains may be more relevant to CD2–CD58 function than isolated sequence changes in CD58 for which no direct functional effect has been demonstrated.

The interaction of CD2 with CD58 occurs during the formation of an immunological synapse between a T lymphocyte or NK cell and an antigen-presenting cell or tumor cell. Binding of both molecules increases intercellular adhesion strength, promotes reorganization of the actin cytoskeleton, and stabilizes the central and peripheral regions of the immunological synapse. This makes TCR signals more stable and effective [14,176,177,178,179]. The spatial organization of CD2–CD58 complexes is therefore functionally important: during immunological synapse formation, these complexes can reorganize into membrane microdomains that help position and retain other costimulatory complexes, linking molecular adhesion to the architecture and efficiency of T cell signaling.

Activation of the CD2/CD58 axis leads to increased T lymphocyte proliferation, cytokine production, and cytotoxic activity. Of particular importance is the increased sensitivity of T lymphocytes to target cells with low antigen expression. This axis lowers the activation threshold and enables more effective recognition of tumor cells that present a limited number of peptide-MHC complexes [180,181]. The importance of CD2/CD58 is particularly evident in immuno-oncology. Loss of CD58 by cancer cells limits stable immunological synapse formation, reduces the activity of T lymphocytes and NK cells, and can lead to resistance to immunotherapy. Experimental studies have shown that CD58 loss impairs immunological synapse formation with CAR-T cells and reduces their expansion, degranulation, cytokine secretion, and cytotoxicity, providing a direct mechanistic link between loss of a surface adhesion molecule and resistance to cell-based immunotherapy [166]. Similarly, CD58 deficiency has been associated with reduced TIL-mediated tumor killing and, in some tumor models, increased PD-L1 expression, suggesting that loss of CD58 can simultaneously weaken costimulatory adhesion and reinforce inhibitory signaling [167]. Importantly, immunohistochemical assessment of CD58 expression has also been investigated in lymphoma cohorts, indicating that the structural loss or downregulation of the ligand can be evaluated at the protein level in clinical material; however, its predictive value for treatment outcome remains context-dependent. This mechanism is also relevant in CAR-T and other cell-based therapies, whose effectiveness depends not only on antigen recognition but also on the quality of contact with the target cell. The CD2/CD58 axis therefore serves as a structural and functional amplifier of the immune response. It does not replace the TCR signal, but stabilizes cell contact, supports the organization of the immunological synapse, and increases the intensity of activation signaling [180,181].

### 4.15. B7-H3 and B7-H4

B7-H3 (CD276) and B7-H4 (VTCN1, B7x, B7S1) belong to the B7 family of ligands and are considered to be new-generation immune checkpoints. Although their functional receptors have not yet been clearly identified, available data indicate that both molecules primarily exert immunosuppressive functions, limiting T cell activation and promoting the development of a tolerogenic tumor microenvironment. Unlike classical B7 family ligands such as PD-L1, B7-H3 and B7-H4 are expressed at low levels in most normal tissues, but are significantly increased in many malignancies. Selective overexpression in tumor tissue makes both molecules attractive diagnostic and therapeutic targets [12,13,182,183,184,185,186].

B7-H3 can be present both on the surface and in the cytoplasm of tumor cells. Its expression is also observed on tumor vascular endothelial cells, tumor-associated fibroblasts, and selected immune cells. This broad localization suggests that B7-H3 participates not only in direct inhibition of the immune response but also in remodeling the tumor stroma and vasculature. B7-H4, by contrast, is found primarily on tumor epithelial cells and tumor-associated macrophages, particularly those of the M2 phenotype. Its expression on endothelial cells is usually low, suggesting a partially distinct role for the two ligands in regulating the tumor microenvironment [12,13,182,183,184,185,186].

**Figure 25:**
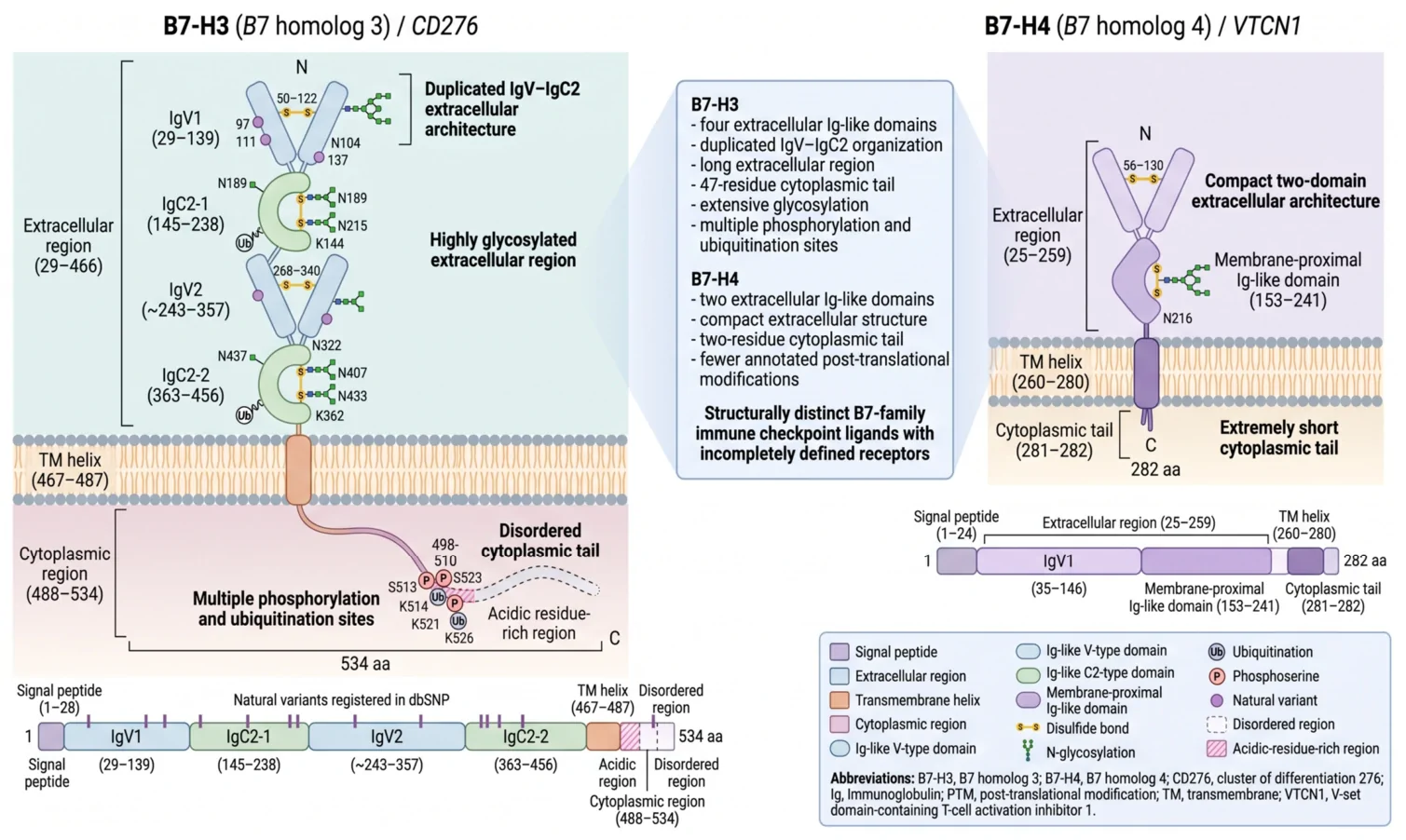
Structural and functional organization of the B7-H3 and B7-H4 immune checkpoint ligands [60,61]. This schematic compares the domain organization, membrane topology, post-translational modifications, and selected sequence features of B7-H3 and B7-H4. B7-H3 displays a duplicated IgV–IgC2 extracellular architecture and a relatively long cytoplasmic region, whereas B7-H4 has a compact two-domain extracellular organization and an extremely short cytoplasmic tail. Purple vertical markers indicate the positions of natural sequence variants registered in dbSNP along the B7-H3 domain map. Abbreviations: aa, amino acids; B7-H3, B7 homolog 3; B7-H4, B7 homolog 4; CD276, cluster of differentiation 276; dbSNP, Single Nucleotide Polymorphism Database; Ig, immunoglobulin; IgC2, immunoglobulin-like C2-type domain; IgV, immunoglobulin-like V-type domain; PTM, post-translational modification; TM, transmembrane; VTCN1, V-set domain-containing T-cell activation inhibitor 1. Created in BioRender. Mertowski, S. (2026) https://BioRender.com/5zo4a5t.

The biological properties of B7-H3 and B7-H4 are closely related to their structural organization. Both proteins are type I transmembrane glycoproteins belonging to the immunoglobulin superfamily. B7-H3 contains an extensive extracellular domain, a single transmembrane segment, and a relatively long cytoplasmic tail. A characteristic feature of the dominant human isoform, B7-H3, is four immunoglobulin-like domains that form two repeating IgV–IgC2 modules. B7-H4 has a shorter structure and includes two immunoglobulin-like domains in the extracellular portion, a transmembrane segment, and a very short cytoplasmic tail. The predominance of the 4Ig isoform of human B7-H3 is structurally relevant because the additional IgV–IgC2 pair increases the size and complexity of the extracellular region and may influence protein interactions and antibody accessibility; importantly, mice predominantly express the 2Ig form, which should be considered when interpreting preclinical studies using human-specific B7-H3-targeting agents. An integrated characterization of the membrane topology, domain organization, posttranslational modifications, sequence variants, and functional regions of B7-H3 and B7-H4 is presented in Figure 25 [60,61]. The described and predicted isoforms of both molecules are summarized in Supplementary Tables S26 and S27.

B7-H3 contains a signal peptide spanning residues 1–28, an extended extracellular domain spanning residues 29–466, a transmembrane segment spanning residues 467–487, and a cytoplasmic domain spanning residues 488–534. The extracellular portion contains the first IgV domain spanning residues 29–139, the first IgC2 domain spanning positions 145–238, the second IgV domain spanning positions 243-, and the second IgC2 domain spanning positions 363–456. This repetitive organization distinguishes human B7-H3 from many other B7 family members. The stability of the B7-H3 structure is ensured by disulfide bridges 50–122, 165–220, 268–340, and 383–438. The protein is extensively N-glycosylated at positions N104, N189, N215, N322, N407, and N433. Ubiquitination at K144 and K362 has also been described in the extracellular portion. The cytoplasmic tail contains phosphorylation sites S513, S523, and S525, and ubiquitination sites K514, K521, and K526. The 498–534 region is disordered, and the 498–510 fragment is enriched in acidic residues, which may favor interactions with regulatory proteins. Numerous natural variants have been identified in B7-H3 at positions 97, 111, 137, 160, 267, 279, 315, 329, and 378 and are recorded in the dbSNP database. Their biological significance remains incompletely defined; therefore, they should be considered primarily as a component of the molecule’s sequence variability. Among these structural features, N-glycosylation has the clearest demonstrated functional significance. Experimental studies have shown that glycosylation at paired sites N91/N309 and N104/N322 is required for efficient trafficking of B7-H3 from the endoplasmic reticulum to the cell surface; disruption of these sites promotes intracellular retention and ER-associated degradation, thereby reducing surface expression. The same glycosylation-dependent regulation affects B7-H3-mediated inhibition of T-cell proliferation and activation, directly linking a posttranslational modification to protein localization and immune function. B7-H4 contains a signal peptide at positions 1–24, an extracellular domain spanning residues 25–259, a transmembrane segment at positions 260–280, and a very short cytoplasmic tail spanning only residues 281–282. The extracellular portion contains two immunoglobulin-like domains: the first IgV domain at positions 35–146 and the second domain spanning residues 153–241. The protein structure is stabilized by disulfide bonds at positions 56–130 and 168–225, while an N-glycosylation site is located at position N216. In contrast to B7-H3, the best-characterized structural determinant of B7-H4 function is its overall glycosylation state rather than a defined receptor-binding interface. Differentially glycosylated B7-H4 forms display markedly different protein stability, with highly glycosylated protein showing a substantially longer half-life than less-glycosylated or unglycosylated forms, indicating that N-glycosylation protects B7-H4 from ubiquitin-dependent degradation [184,185,186]. This provides a direct link between a bioinformatic PTM annotation and experimentally demonstrated protein stability, cellular abundance, and persistence of B7-H4 at the tumor-cell surface. The mechanisms of action of B7-H3 and B7-H4 have not yet been fully elucidated, primarily due to the lack of clearly identified receptors. However, available data indicate that both molecules limit T cell activation, reducing their proliferation, IL-2 and IFN-γ production, and cytotoxic activity. This effect attenuates the antitumor response and promotes the maintenance of local immunosuppression [182,183,184,185,186].

B7-H3 also exhibits numerous activities independent of direct immune regulation. Its overexpression may support tumor cell proliferation and survival, epithelial–mesenchymal transition, angiogenesis, the maintenance of stem cells, and the development of invasion and metastasis. B7-H3 may also influence tumor metabolism and interactions among tumor cells, the endothelium, and the stroma, further strengthening its role in disease progression [187].

B7-H4 is more strongly associated with the maintenance of an immunosuppressive tumor microenvironment. Its expression on tumor cells and M2 macrophages may limit the activity of effector lymphocytes and promote a tolerogenic phenotype of myeloid cells. This molecule may also promote the survival of dormant tumor cells, especially after hormonal therapy for prostate cancer, suggesting its potential involvement in late disease relapse [187,188,189]. B7-H3 and B7-H4 therefore have partially distinct but complementary functions in the tumor microenvironment. B7-H3 exerts broad effects on tumor cells, the stroma, and the tumor vasculature, whereas B7-H4 is more strongly associated with inhibiting T cell responses and supporting immunosuppressive myeloid cells [187,188,189]. The glycosylation-dependent regulation of these proteins also has potential analytical and therapeutic implications. For B7-H3, glycan-dependent surface localization can influence the amount of antigen available for antibody-based detection and targeting, while glycoform-specific recognition may distinguish tumor-associated B7-H3 from other molecular forms of the protein [185]. Indeed, a monoclonal antibody preferentially recognizing glycosylated B7-H3 at N91/N309 and N104/N322 promoted B7-H3 internalization and degradation, illustrating how PTM-dependent differences in the molecular form of a checkpoint can be exploited for selective therapeutic targeting [186,187,188,189]. For B7-H4, the demonstrated relationship between glycosylation, ubiquitination, and protein half-life indicates that the measured abundance of the protein may depend not only on transcriptional expression but also on posttranslational regulation of its stability.

## 5. Therapeutic Significance and Biomarker Potential of Immune Checkpoints

Understanding the biology of immune checkpoints has fundamentally changed our understanding of the relationship between the immune system and cancer cells. Cancers exploit physiological mechanisms that limit excessive immune activation, weakening effector lymphocyte function and evading immune surveillance. The discovery of these relationships laid the foundation for the development of therapies that block co-inhibitory signals or enhance costimulatory signals, which currently constitute one of the pillars of treatment for many cancers [189,190,191]. However, the importance of checkpoints extends beyond their role as therapeutic targets. Receptor and ligand expression, cellular localization, coexistence with other regulatory molecules, and the presence of soluble forms can reflect the degree of immune cell activation or exhaustion, the severity of immunosuppression, and the properties of the tumor microenvironment. These parameters can therefore provide prognostic, predictive, and monitoring information [190,191,192].

### 5.1. Immune Checkpoints as Therapeutic Targets

Therapeutic strategies targeting immune checkpoints primarily include blocking co-inhibitory receptors and ligands and agonistic activation of costimulatory receptors. The former approach leads to the abolition of inhibitory signals and the reactivation of effector cells, while the latter aims to enhance their proliferation, survival, metabolic activity, and cytotoxic function [134]. The greatest clinical success has been achieved by blocking the PD-1/PD-L1 axis and CTLA-4. PD-1 and PD-L1 inhibitors restore the antitumor activity of T lymphocytes in the tumor microenvironment, while CTLA-4 blockade primarily affects the primary activation of lymphocytes in lymphoid organs. Combination therapy may enhance antitumor efficacy but is also associated with a higher risk of immune-mediated adverse events [71,79,81]. A reverse strategy is offered by CTLA4-Ig fusion proteins, which limit CD28-dependent costimulation and are used in the treatment of autoimmune diseases and transplantation [81].

Primary or acquired resistance to PD-1/PD-L1 inhibitors may result from compensatory activation of other inhibitory receptors. Therefore, therapies targeting BTLA, TIGIT, CD112R, CD96, TIM-3, LAG-3, and VISTA are being intensively developed. Their blockade is most often evaluated in combination with PD-1/PD-L1 inhibitors, as coexpression of multiple inhibitory receptors is characteristic of profoundly exhausted T cells and NK cells [88,95,96,97,105,111,115]. The most clinically advanced new checkpoint is LAG-3. The combination of relatlimab with nivolumab confirmed that simultaneous inhibition of multiple coinhibitory pathways can provide therapeutic benefits. Bispecific antibodies and strategies modulating LAG-3 interactions with FGL1 and MHC class II are also being developed [86,87]. In the case of VISTA, its activity in the acidic, myeloid-rich tumor microenvironment and its potential to contribute to adaptive resistance to PD-1/PD-L1 blockade are of particular importance [88,89,90]. B7-H3 and B7-H4 are being developed primarily as target antigens for antibody-drug conjugates, cytotoxic antibodies, and cell-based therapies. Their therapeutic value stems from their high expression in many cancers and relatively limited presence in normal tissues [188,189]. A second major direction in immunotherapy is the activation of costimulatory receptors, such as OX40, ICOS, 4-1BB, GITR, CD27, and CD40. Agonism of these receptors is expected to increase the survival and activity of effector lymphocytes, support memory responses, and improve NK cell function. However, the effectiveness of this approach depends on proper receptor clustering, signal localization in the tumor microenvironment and limited systemic toxicity [133,135,148,149,150,151,152,153,154,155,158].

A particularly important example is 4-1BB, whose signaling domain is widely used in CAR-T constructs due to its beneficial effects on cell survival, mitochondrial metabolism, and the development of a memory phenotype [148,149,150]. Modulation of the CD27/CD70 axis can involve both agonism of CD27 and blocking or directly targeting CD70 on cancer cells [162,166]. CD40 agonism primarily serves to activate and “license” dendritic cells, while blocking CD40/CD40L is developed in autoimmune and inflammatory diseases [168,171,174,175]. The therapeutic importance of the CD2/CD58 axis stems primarily from its role in organizing the immunological synapse. Loss of CD58 by cancer cells can impair contact with lymphocytes and lead to resistance to cell-based therapies. In turn, the use of CD2 signaling in CAR-T constructs can improve synapse stability and the cytotoxic activity of effector cells [180,181]. The most important therapeutic strategies, exemplary molecules, and their mechanisms of action are summarized in Table 5.

### 5.2. Immune Checkpoints as Potential Biomarkers

Checkpoints are promising candidate biomarkers because they reflect both the properties of tumor cells and the functional state of the immune response. Their expression may indicate lymphocyte activation or exhaustion, the dominance of regulatory populations, myeloid infiltration, and the initiation of adaptive resistance mechanisms [193,194,195,196]. The best-studied example is PD-L1, whose expression in selected tumors types can be used to inform treatment decisions regarding PD-1 or PD-L1 inhibitors. However, the value of this biomarker remains limited, as responses can also occur in patients with low expression, and some patients with high PD-L1 levels do not benefit from therapy [197,198]. In the case of new checkpoints, analysis of their co-expression may be more valuable than the determination of a single molecule. The concomitant presence of PD-1, TIM-3, LAG-3, TIGIT, CD112R, or VISTA may identify lymphocytes with a more pronounced exhaustion phenotype and may be associated with resistance to single-agent checkpoint blockade. Regarding costimulatory receptors, it is important to determine the cell population on which they are expressed. The presence of OX40 or ICOS on effector lymphocytes may have a different significance than their expression on Tregs [133,137,138,139,140,141,142,143,144,145]. The available evidence supporting the biomarker potential of individual immune checkpoint molecules varies substantially, from clinically established applications in specific contexts to findings based mainly on observational or experimental studies. A summary of the current level of clinical evidence for selected immune checkpoint receptors and ligands is provided in Table 6.

The expression of antigens that are direct therapeutic targets may also be of biomarker significance. This is particularly true for CD70, B7-H3, and B7-H4, as the level, location, and uniformity of their expression may influence the efficacy of antibody-drug conjugates and CAR-T therapy. High expression of B7-H3 or B7-H4 often correlates with disease progression, tumor biological aggressiveness, and shorter survival, although these associations remain tumor-type specific [186,187]. Changes in the expression of these molecules, including loss of expression in specific contexts, may also have prognostic relevance. CD58 deficiency impairs immunological synapse formation and may promote resistance to T lymphocytes, NK cells, and CAR-T therapy [180]. Similarly, changes in the expression of TIM-3, LAG-3, TIGIT, or VISTA during treatment with PD-1/PD-L1 inhibitors have been investigated in relation to adaptive resistance mechanisms.

Soluble forms of receptors and ligands, as well as molecules present in extracellular vesicles, are also potential biomarker sources. Their concentrations may reflect immune activation, proteolytic protein release, alternative transcript splicing, or tumor burden. Analysis of PD-L1 or B7-H3 in extracellular vesicles has been investigated as a potential approach for noninvasive monitoring of changes in the tumor microenvironment and treatment response [186,187]. Full utilization of the biomarker potential of checkpoints is limited by the spatial and temporal heterogeneity of their expression. Results obtained from a single tumor fragment may not reflect the entire microenvironment, and marker levels can change due to treatment, hypoxia, cytokines, infection, and disease progression. Differences among analytical platforms, diagnostic antibodies, positivity thresholds, and assessment methods remain a challenge [196,197].

Therefore, multiparameter signatures that integrate the expression of co-inhibitory and costimulatory receptors, the composition of the immune infiltrate, the presence of Tregs and myeloid cells, the molecular characteristics of the tumor, and the concentrations of soluble and vesicular forms are increasingly being investigated as an alternative to the analysis of individual molecules. Such approaches may help to improve the identification of resistance mechanisms and refine prognostic assessment, while their potential to guide treatment selection remains under investigation. Their implementation into clinical practice, however, requires standardization of methods and validation in large, prospective studies [196,197].

## 6. Conclusions

Immune checkpoints play a key role in maintaining the balance between activation and inhibition of the immune response, and dysregulation of these pathways contributes to the development and progression of cancer as well as other immune-mediated and inflammatory conditions. The rapid development of research into their biology has significantly expanded our understanding of the mechanisms regulating immune responses and provided the basis for the development of immune checkpoint-targeted therapeutic strategies. At the same time, a growing body of data indicates that the relevance of immune checkpoints extends beyond their role as therapeutic targets, encompassing their potential value as molecular indicators of immune activity and disease-associated immune dysregulation. Analysis of their expression, molecular characteristics, and interactions may provide valuable insights into tumor biology, the immune microenvironment, and inflammatory processes, and may support the identification of candidate diagnostic, prognostic, and predictive biomarkers. However, the clinical validity and applicability of these biomarkers remain highly context-dependent and vary substantially between individual immune checkpoint molecules, disease settings, and analytical approaches. Further development of molecular technologies, standardized analytical approaches, and comprehensive multiparameter analyses, together with prospective clinical validation, could facilitate the translation of immune checkpoint biology into precision medicine and more personalized management of cancer and immune-mediated diseases.

## Figures and Tables

**Table 1:** Molecular and physicochemical characteristics of co-stimulatory immune checkpoint receptors [16,29,30,31,32,33,34,35,36,37,38].

Protein (UniProt Accession No.)	Gene	Length (aa)	Molecular Weight (kDa)	Hydrophobic Residues (%)	Hydrophilic Residues (%)	Theoretical pI
GITR (Q9Y5U5)	*TNFRSF18*	241	26.000	61.00	39.00	5.66
CD2 (P06729)	*CD2*	351	39.448	43.02	56.98	8.80
CD28 (P10747)	*CD28*	220	25.066	53.95	46.05	8.93
ICOS (Q9Y6W8)	*ICOS*	199	22.625	54.77	45.23	7.94
OX40 (P43489)	*TNFRSF4*	277	29.341	56.68	43.32	7.37
4-1BB (Q07011)	*TNFRSF9*	255	27.899	52.55	47.45	6.85
CD27 (P26842)	*CD27*	260	29.137	52.31	47.69	6.77
CD40 (P25942)	*CD40*	277	30.619	51.62	48.38	5.30
CD226 (Q15762)	*CD226*	336	38.614	47.32	52.68	7.18
HVEM (Q92956)	*TNFRSF14*	283	30.392	55.83	44.17	6.32

Abbreviations: aa, amino acids; CD, cluster of differentiation; GITR, glucocorticoid-induced tumor necrosis factor receptor-related protein; HVEM, herpesvirus entry mediator; ICOS, inducible T-cell co-stimulator; kDa, kilodalton; OX40, tumor necrosis factor receptor superfamily member 4; pI, isoelectric point; TNFRSF, tumor necrosis factor receptor superfamily; UniProt, Universal Protein Resource; 4-1BB, tumor necrosis factor receptor superfamily member 9.

**Table 2:** Molecular and physicochemical characteristics of co-stimulatory immune checkpoint ligands [16,39,40,41,42,43,44,45,46,47,48].

Protein (UniProt Accession No.)	Gene	Cognate Receptor(s)	Length (aa)	Molecular Weight (kDa)	Hydrophobic Residues (%)	Hydrophilic Residues (%)	Theoretical pI
CD80 (P33681)	*CD80*	CD28, CTLA-4	288	33.480	37.50	62.50	6.82
CD86 (P42081)	*CD86*	CD28, CTLA-4	329	37.682	45.29	54.71	6.14
ICOSL (O75144)	*ICOSLG*	ICOS	302	33.349	48.14	51.86	5.02
OX40L (P23510)	*TNFSF4*	OX40	183	21.050	47.54	52.46	6.66
4-1BBL (P41273)	*TNFSF9*	4-1BB	254	26.625	64.57	35.43	6.27
GITRL (Q9UNG2)	*TNFSF18*	GITR	177	20.308	53.67	46.33	7.00
CD70 (P32970)	*CD70*	CD27	193	21.118	56.48	43.52	7.77
CD40L (P29965)	*CD40LG*	CD40	261	29.274	47.51	52.49	7.57
CD58 (P19256)	*CD58*	CD2	250	28.147	48.80	51.20	5.98
LIGHT (O43557)	*TNFSF14*	HVEM, LTβR; DcR3 as a decoy receptor	240	26.350	56.67	43.33	8.57

Abbreviations: aa, amino acids; CD, cluster of differentiation; CD40L, CD40 ligand; GITRL, glucocorticoid-induced tumor necrosis factor receptor ligand; ICOSL, inducible T-cell co-stimulator ligand; kDa, kilodalton; LIGHT, lymphotoxin-like, exhibits inducible expression, and competes with herpes simplex virus glycoprotein D for binding to HVEM, a receptor expressed by T lymphocytes; OX40L, OX40 ligand; pI, isoelectric point; TNFSF, tumor necrosis factor ligand superfamily; UniProt, Universal Protein Resource; 4-1BBL, 4-1BB ligand.

**Table 3:** Molecular and physicochemical characteristics of co-inhibitory immune checkpoint receptors [16,50,51,52,53,54,55,56,57,58,59,60,61,62,63].

Protein (UniProt Accession No.)	Gene	Length (aa)	Molecular Weight (kDa)	Hydrophobic Residues (%)	Hydrophilic Residues (%)	Theoretical pI
BTLA (Q7Z6A9)	*BTLA*	289	32.834	47.75	52.25	5.80
CTLA-4 (P16410)	*CTLA4*	223	24.656	63.23	36.77	6.30
PD-1 (Q15116)	*PDCD1*	288	31.647	57.64	42.36	7.26
TIGIT (Q495A1)	*TIGIT*	244	26.319	58.20	41.80	5.31
LAG-3 (P18627)	*LAG3*	525	57.449	60.38	39.62	7.17
TIM-3 (Q8TDQ0)	*HAVCR2*	301	33.394	59.14	40.86	5.39
VISTA (Q9H7M9)	*VSIR*	311	33.908	55.63	44.37	6.13
CD112R (Q6DKI7)	*PVRIG*	326	34.344	62.58	37.42	8.24
CEACAM1 (P13688)	*CEACAM1*	526	57.560	49.81	50.19	5.44
CD96 (P40200)	*CD96*	585	65.634	49.40	50.60	6.33
PSGL-1 (Q14242)	*SELPLG*	412	43.201	53.16	46.84	4.27
CD160 (O95971)	*CD160*	181	19.810	53.59	46.41	6.51

Abbreviations: aa, amino acids; BTLA, B- and T-lymphocyte attenuator; CD, cluster of differentiation; CD112R, CD112 receptor; CEACAM1, carcinoembryonic antigen-related cell adhesion molecule 1; CTLA-4, cytotoxic T-lymphocyte-associated protein 4; kDa, kilodalton; LAG-3, lymphocyte activation gene 3; PD-1, programmed cell death protein 1; pI, isoelectric point; PSGL-1, P-selectin glycoprotein ligand 1; TIGIT, T-cell immunoreceptor with immunoglobulin and ITIM domains; TIM-3, T-cell immunoglobulin and mucin-domain containing-3; UniProt, Universal Protein Resource; VISTA, V-domain immunoglobulin suppressor of T-cell activation.

**Table 4:** Molecular and physicochemical characteristics of co-inhibitory immune checkpoint ligands [16,64,65,66,67,68,69].

Protein (UniProt Accession No.)	Gene	Cognate Receptor(s)	Length (aa)	Molecular Weight (kDa)	Hydrophobic Residues (%)	Hydrophilic Residues (%)	Theoretical pI
PD-L1 (Q9NZQ7)	*CD274*	PD-1	290	33.275	51.03	48.97	6.44
PD-L2 (Q9BQ51)	*PDCD1LG2*	PD-1	273	30.957	51.59	44.87	7.26
Galectin-9 (O00182)	*LGALS9*	TIM-3	355	39.518	57.75	42.25	8.68
FGL1 (Q08830)	*FGL1*	LAG-3	312	36.379	49.36	50.64	5.40
CD155 (P15151)	*PVR*	TIGIT, CD96, CD226	417	45.303	57.55	42.45	5.79
CD112 (Q92692)	*NECTIN2*	CD112R, TIGIT, CD226	538	57.742	55.95	44.05	4.66

Abbreviations: aa, amino acids; CD, cluster of differentiation; CD112R, CD112 receptor; FGL1, fibrinogen-like protein 1; Galectin-9, galectin-9; kDa, kilodalton; LAG-3, lymphocyte activation gene 3; PD-1, programmed cell death protein 1; PD-L1, programmed death-ligand 1; PD-L2, programmed death-ligand 2; pI, isoelectric point; PVR, poliovirus receptor; TIGIT, T-cell immunoreceptor with immunoglobulin and ITIM domains; TIM-3, T-cell immunoglobulin and mucin-domain containing-3; UniProt, Universal Protein Resource.

**Table 5:** Main therapeutic strategies targeting ICPs.

Axis or Target	Type of Modulation	Examples of Drugs or Strategies	Main Mechanism of Action	Main Application or Development Direction
PD-1/PD-L1	Co-inhibitory blockade	Pembrolizumab, nivolumab, cemiplimab; atezolizumab, durvalumab, avelumab	Removal of inhibitory signaling and reactivation of T cells	Multiple solid and hematological malignancies [75,79]
CTLA-4	Co-inhibitory blockade	Ipilimumab	Increased availability of CD80/CD86 for CD28 and enhanced T-cell priming	Cancer immunotherapy, often combined with anti-PD-1 therapy [81]
CD28–CD80/CD86	Inhibition of co-stimulation	Abatacept, belatacept	Binding of CD80/CD86 and blockade of CD28 activation	Autoimmune diseases and transplantation [81]
BTLA/HVEM	Blockade or selective modulation	Icatolimab, tifcemalimab; HVEM modulators	Attenuation of BTLA signaling and restoration of lymphocyte activity	Combination therapies in oncology [88,89]
TIGIT/CD112R/CD96–CD226	Blockade of inhibitory receptors	Anti-TIGIT, anti-CD112R, and anti-CD96 antibodies	Restoration of the predominance of CD226-mediated co-stimulation	Combination therapies with anti-PD-1/PD-L1 agents [92,100,101]
TIM-3/Galectin-9/CEACAM1	Co-inhibitory blockade	Sabatolimab, cobolimab, LY3321367, Sym023, INCAGN02390	Reduction in lymphocyte exhaustion and resistance to PD-1 blockade	Solid and hematological malignancies [105,111,115]
LAG-3/FGL1–MHC-II	LAG-3 blockade or APC activation	Relatlimab, favezelimab, fianlimab, ieramilimab; eftilagimod alfa	Reactivation of lymphocytes or stimulation of APCs through MHC-II	Melanoma and other malignancies [119,120]
VISTA/PSGL-1	Blockade or agonism	SNS-101, KVA12123, HMBD-002; small-molecule inhibitors; VISTA agonists	Reversal of pH-dependent immunosuppression or induction of immune tolerance	Oncology, autoimmune diseases, and transplantation [122,127]
OX40/OX40L	OX40 agonism	Ivuxolimab, MOXR0916, MEDI0562, BMS-986178, INBRX-106	OX40 clustering and enhancement of lymphocyte survival and function	Cancer immunotherapy [133,135]
ICOS/ICOSL	Agonism or blockade	Vopratelimab, feladilimab; prezalumab, MEDI-570, acazicolcept	Enhancement of effector responses or suppression of Tfh/Treg responses	Oncology and autoimmune diseases [137,139]
4-1BB/4-1BBL	Targeted agonism	Urelumab, utomilumab; bispecific antibodies; 4-1BB signaling domain in CAR-T cells	Enhancement of cytotoxicity, mitochondrial metabolism, and memory formation	Immunotherapy and CAR-T-cell therapies [148,149,150]
GITR/GITRL	GITR agonism	TRX518, MK-4166, BMS-986156, AMG-228, MEDI1873	Activation of effector cells and potential reduction in Treg-mediated suppression	Cancer immunotherapy [153,154,155,156,157,158,159]
CD27/CD70	CD27 agonism or CD70 blockade	Varlilumab, cusatuzumab, ADCs, CD70 CAR-T cells	Lymphocyte activation or elimination of CD70-positive cells	Malignancies with CD70 overexpression [165,166]
CD40/CD40L	CD40 agonism or axis blockade	Anti-CD40 agonists; dapirolizumab pegol, VIB4920, letolizumab, TRAF-STOPs	APC maturation or suppression of pathological immune activation	Oncology, autoimmune diseases, and inflammation [168,171,174,175]
CD2/CD58	Modulation of adhesion and immune-synapse formation	Siplizumab, CD58 fusion proteins, CAR-T constructs incorporating CD2 signaling	Modulation of lymphocyte activation and immunological-synapse stability	Cellular therapies and immunosuppression [180,181]
B7-H3	ADCs, antibodies, CAR-T cells	Ifinatamab deruxtecan, vobramitamab duocarmazine, enoblituzumab, B7-H3 CAR-T cells	Selective elimination of B7-H3-positive cells	Solid tumors [188,189]
B7-H4	ADCs and antibodies	AZD8205 and other anti-B7-H4 agents	Delivery of a cytotoxic payload to B7-H4-positive cells	Malignancies with B7-H4 overexpression [188,189]

**Table 6:** Clinical evidence supporting the biomarker potential of selected immune checkpoint receptors and ligands.

Immune Checkpoint	Biomarker Application	Disease/Context	Current Clinical Evidence
PD-L1	Predictive; prognostic	NSCLC, melanoma, urothelial carcinoma, HCC	Clinically established in selected clinical contexts; assay- and cutoff-dependent [70,72,78,195,196,197,198]
PD-1	Prognostic; predictive candidate	NSCLC, melanoma, colorectal cancer	Investigated clinically; context-dependent evidence [23,24,79,195]
CTLA-4	Prognostic; predictive candidate	Melanoma, NSCLC, colorectal cancer	Investigated clinically; context-dependent evidence [18,23,25,80,192]
LAG-3	Predictive; prognostic	Melanoma, NSCLC, other solid tumors	Promising biomarker under clinical investigation [116,119,121,122,123,195]
TIGIT	Predictive; prognostic; resistance-associated	NSCLC, melanoma, colorectal cancer	Emerging biomarker; mainly observational/clinical-trial evidence [93,95,102,103,195]
TIM-3	Prognostic; predictive; treatment-monitoring candidate	NSCLC, HCC, melanoma	Emerging biomarker; mainly observational evidence [104,105,106,107,108,109,110,111,115,195]
VISTA	Prognostic; predictive candidate	Breast cancer, melanoma, NSCLC	Limited clinical validation [124,125,126,127,128,129]
B7-H3	Prognostic; predictive candidate	Prostate cancer, ovarian cancer, esophageal cancer	Mainly retrospective/observational evidence [185,186,187,188,189]
B7-H4	Prognostic; predictive candidate	Prostate cancer, ovarian cancer, esophageal cancer	Mainly retrospective/observational evidence [182,183,184,185,186,187,188]
CD70	Predictive; prognostic; treatment-selection candidate	Renal cell carcinoma, glioma, lymphoma, nasopharyngeal carcinoma	Emerging/experimental biomarker [161,162,163,164,165,166,167]
CD58	Resistance-associated; predictive candidate	Hematological malignancies, CAR-T therapy settings	Experimental biomarker candidate [180,181]

The evidence categories reflect the current maturity of available biomarker data and do not represent a formally validated grading system.

## Data Availability

No new data were created or analyzed in this study. Data sharing is not applicable to this article.

## Supplementary Materials

The following supporting information can be downloaded at: https://www.mdpi.com/article/10.3390/antib15050084/s1, Table S1: Bioinformatic characteristics of reviewed protein isoforms (PD-1/PD-L1/PD-L2); Table S2: Bioinformatic characteristics of predicted protein isoforms (PD-1/PD-L1/PD-L2); Table S3: Bioinformatic characteristics of reviewed protein isoforms (CTLA-4/CD28/CD80/CD86); Table S4: Bioinformatic characteristics of predicted protein isoforms (CTLA-4/CD28/CD80/CD86); Table S5: Bioinformatic characteristics of reviewed protein isoforms (CD160/BTLA–HVEM/LIGHT); Table S6: Bioinformatic characteristics of predicted protein isoforms (CD160/BTLA–HVEM/LIGHT); Table S7: Bioinformatic characteristics of reviewed protein isoforms (CD226/TIGIT/CD96/CD112R–CD155/CD112); Table S8: Bioinformatic characteristics of predicted protein isoforms (CD226/TIGIT/CD96/CD112R–CD155/CD112); Table S9: Bioinformatic characteristics of reviewed protein isoforms (TIM-3/Galectin-9/CEACAM1); Table S10: Bioinformatic characteristics of predicted protein isoforms (TIM-3/Galectin-9/CEACAM1); Table S11: Bioinformatic characteristics of reviewed protein isoforms (LAG-3/FGL1); Table S12: Bioinformatic characteristics of predicted protein isoforms (LAG-3/FGL1); Table S13: Bioinformatic characteristics of reviewed protein isoforms (VISTA/PSGL-1); Table S14: Bioinformatic characteristics of predicted protein isoforms (VISTA/PSGL-1); Table S15: Bioinformatic characteristics of reviewed protein isoforms (OX40/OX40L); Table S16: Bioinformatic characteristics of predicted protein isoforms (OX40/OX40L); Table S17: Bioinformatic characteristics of reviewed protein isoforms (ICOS/ICOSL); Table S18: Bioinformatic characteristics of predicted protein isoforms (ICOS/ICOSL); Table S19: Bioinformatic characteristics of predicted protein isoforms (4-1BB/4-1BBL); Table S20: Bioinformatic characteristics of predicted protein isoforms (GITR/GITRL); Table S21: Bioinformatic characteristics of reviewed protein isoforms (CD27/CD70); Table S22: Bioinformatic characteristics of predicted protein isoforms (CD27/CD70); Table S23: Bioinformatic characteristics of predicted protein isoforms (CD40/CD40L); Table S24: Bioinformatic characteristics of reviewed protein isoforms (CD2/CD58); Table S25: Bioinformatic characteristics of predicted protein isoforms (CD2/CD58); Table S26: Bioinformatic characteristics of reviewed protein isoforms (B7-H3 and B7-H4); Table S27: Bioinformatic characteristics of predicted B7-H3 and B7-H4 protein isoforms.

## Abbreviations

4-1BB	4-1BB receptor (CD137; TNFRSF9)
4-1BBL	4-1BB ligand (TNFSF9)
aa	Amino acid(s)
ADCC	Antibody-dependent cellular cytotoxicity
AML	Acute myeloid leukemia
AP-1	Activator protein 1
APC	Antigen-presenting cell
ATL	Adult T-cell leukemia/lymphoma
BCR	B-cell receptor
BTLA	B- and T-lymphocyte attenuator
CAR-T	Chimeric antigen receptor T cell
CD	Cluster of differentiation
CEACAM1	Carcinoembryonic antigen-related cell adhesion molecule 1
CRD	Cysteine-rich domain
CTLA-4	Cytotoxic T-lymphocyte-associated protein 4
CVID	Common variable immunodeficiency
Da	Dalton
DC	Dendritic cell
DNAM-1	DNAX accessory molecule-1 (CD226)
EMT	Epithelial–mesenchymal transition
ERK	Extracellular signal-regulated kinase
FcγR	Fc gamma receptor
FGL1	Fibrinogen-like protein 1
GITR	Glucocorticoid-induced TNFR-related protein
GITRL	Glucocorticoid-induced TNFR-related protein ligand
GVHD	Graft-versus-host disease
HAVCR2	Hepatitis A virus cellular receptor 2
HMGB1	High mobility group box 1
HVEM	Herpesvirus entry mediator
ICOS	Inducible T-cell costimulator
ICOSL	Inducible T-cell costimulator ligand
IFN-γ	Interferon gamma
IgSF	Immunoglobulin superfamily
IL	Interleukin (e.g., IL-2, IL-10, IL-12)
ICP	Immune checkpoint
irAEs	Immune-related adverse events
ITIM	Immunoreceptor tyrosine-based inhibitory motif
ITSM	Immunoreceptor tyrosine-based switch motif
ITT-like	Immunoreceptor tyrosine-based inhibitory motif-like motif
LAG-3	Lymphocyte activation gene 3
LFA-3	Lymphocyte function-associated antigen 3 (CD58)
LIGHT	Homologous to lymphotoxins, exhibits inducible expression, competes with HSV glycoprotein D for HVEM, a receptor expressed by T lymphocytes
MAPK	Mitogen-activated protein kinase
MDSCs	Myeloid-derived suppressor cells
MHC	Major histocompatibility complex
mAb	Monoclonal antibody
NECTIN2	Nectin cell adhesion molecule 2
NFAT	Nuclear factor of activated T cells
NK	Natural killer cell
OX40	OX40 receptor (TNFRSF4)
OX40L	OX40 ligand (TNFSF4)
PBMC	Peripheral blood mononuclear cells
PD-1	Programmed cell death protein 1
PD-L1	Programmed death-ligand 1
PD-L2	Programmed death-ligand 2
PI3K/AKT	Phosphoinositide 3-kinase/Protein kinase B signaling pathway
pI	Theoretical isoelectric point
PSGL-1	P-selectin glycoprotein ligand-1
PtdSer	Phosphatidylserine
PVR	Poliovirus receptor
PVRIG	Poliovirus receptor-related immunoglobulin domain-containing protein
ROS	Reactive oxygen species
SELPLG	Selectin P ligand gene
TAM	Tumor-associated macrophage
TCR	T-cell receptor
Tfh	T follicular helper cell
TIGIT	T-cell immunoreceptor with Ig and ITIM domains
TILs	Tumor-infiltrating lymphocytes
TIM-3	T-cell immunoglobulin and mucin-domain containing-3
TLS	Tertiary lymphoid structures
TMB	Tumor mutational burden
TME	Tumor microenvironment
TNF	Tumor necrosis factor
TNFRSF	Tumor necrosis factor receptor superfamily
TNFSF	Tumor necrosis factor superfamily
TRAF1	TNF receptor-associated factor 1
TRAF2	TNF receptor-associated factor 2
TRAF3	TNF receptor-associated factor 3
Treg	Regulatory T cell
VISTA	V-domain Ig suppressor of T-cell activation
VSIR	V-set immunoregulatory receptor
