# Supplementary material for: Molecular Architecture and Clinical Landscape of Immune Checkpoint Receptors and Ligands
Source: Antibodies (Basel). 2026 Sep 9;15(5):84. doi: 10.3390/antib15050084 (PMC13600086; doi:10.3390/antib15050084)
Supplement: Supplementary file 1 [file antibodies-15-00084-s001.zip › antibodies-4495186-supplementary.pdf]

Supplementary Material S1

PD-1/PD-L1/PD-L2 immune checkpoint axis

Supplementary Tables S1 and S2 present bioinformatic characteristics of reviewed PD-L1 and PD-L2 isoforms and predicted PD-1 and PD-L2 isoforms, respectively. The reported data include isoform identifiers, gene symbols, length (aa), molecular weight (Da), percentages of hydrophobic and hydrophilic residues, and theoretical isoelectric point (pI).

Supplementary Table S1. Bioinformatic characteristics of reviewed protein isoforms (PD-1/PD-L1/PD-L2).

Protein	Isoform	Gene	Length (aa)	Molecular weight (Da)	Hydrophobic residues (%)	Hydrophilic residues (%)	Theoretical isoelectric point (pI)
PD-L1	Q9NZQ7-2	*CD274*	176	20,246	46.24	53.76	6.74
	Q9NZQ7-3	*CD274*	178	20,454	55.06	44.94	5.49
	Q9NZQ7-4	*CD274*	245	28,050	51.6	48.4	6.04
PD-L2	Q9BQ51-2	*PDCD1LG2*	183	20,867	55.19	44.81	7.47
	Q9BQ51-3	*PDCD1LG2*	182	20,745	49.45	50.55	6.6

Supplementary Table S2. Bioinformatic characteristics of predicted protein isoforms (PD-1/PD-L1/PD-L2).

Protein	Isoform	Gene	Length (aa)	Molecular weight (Da)	Hydrophobic residues (%)	Hydrophilic residues (%)	Theoretical isoelectric point (pI)
PD-1	H0Y2W6	*PDCD1*	236	26,233	55.08	44.92	5.94
	A0ABB0MVH4	*PDCD1*	287	31,519	57.84	42.16	6.94
	A0ABB0MVI3	*PDCD1*	198	21,871	58.59	41.41	8.71
	A0ACI8QX60	*PDCD1*	274	30,052	58.03	41.97	7.52
	A0ACI8UC53	*PDCD1*	222	24,638	55.41	44.59	6.47
	E7ER21	*PDCD1*	66	7,420	62.12	37.88	3.92
	A0A0G2JRY2	*PDCD1*	122	13,720	49.59	50.41	8.07
PD-L2	A0ACI8VEQ9	PDCD1LG2	287	32,665	52.61	47.39	8.31

Supplementary Material S2

CTLA-4/CD28/CD80/CD86 immune checkpoint axis

Supplementary Tables S3 and S4 present bioinformatic characteristics of reviewed CTLA-4, CD80, and CD86 isoforms and predicted CTLA-4, CD28, CD80, and CD86 isoforms, respectively. The reported data include isoform identifiers, gene symbols, length (aa), molecular weight (Da), percentages of hydrophobic and hydrophilic residues, and theoretical isoelectric point (pI).

Supplementary Table S3. Bioinformatic characteristics of reviewed protein isoforms (CTLA-4/CD28/CD80/CD86).

Protein	Isoform	Gene	Length (aa)	Molecular weight (Da)	Hydrophobic residues (%)	Hydrophilic residues (%)	Theoretical isoelectric point (pI)
CTLA-4	P16410-2	*CTLA4*	56	6,560	66.07	33.93	7.51
	P16410-3	*CTLA4*	58	6,745	60.34	39.66	9.37
	P16410-4	*CTLA4*	79	8,855	62.03	37.97	9.71
	P16410-5	*CTLA4*	174	19,145	61.49	38.51	7.7
CD80	P33681-2	*CD80*	256	29,413	38.41	61.59	7.18
	P33681-3	*CD80*	162	18,761	41.98	58.02	8.64
CD86	P42081-2	*CD86*	110	12,818	48.18	51.82	7.83
	P42081-3	*CD86*	323	37,007	47.99	52.01	6.35
	P42081-4	*CD86*	275	31,293	45.45	54.55	5.6
	P42081-5	*CD86*	217	24,773	47.93	52.07	6.05
	P42081-6	*CD86*	247	28,420	45.34	54.66	7.29

Supplementary Table S4. Bioinformatic characteristics of predicted protein isoforms (CTLA-4/CD28/CD80/CD86).

Protein	Isoform	Gene	Length (aa)	Molecular weight (Da)	Hydrophobic residues (%)	Hydrophilic residues (%)	Theoretical isoelectric point (pI)
CTLA-4	A0A8Q3SIR7	CTLA4	247	27,248	62.35	37.65	7.04
	A0A8Q3WKZ2	CTLA4	203	22,476	63.05	36.95	5.84
CD28	A0ABB0MVH3	CD28	115	12,648	64.35	35.65	9.33
CD80	A0N0P2	*CD80*	288	33,048	47.57	52.43	6.82
CD86	H7C4F8	*CD86*	277	31,505	39.53	60.47	5.6
	C9JXS1	*CD86*	167	18,996	49.10	50.90	6

Supplementary Material S3

CD160/BTLA–HVEM/LIGHT immune checkpoint axis

Supplementary Tables S5 and S6 present bioinformatic characteristics of reviewed CD160, BTLA, and LIGHT isoforms and predicted CD160, HVEM, and LIGHT isoforms, respectively. The reported data include isoform identifiers, gene symbols, length (aa), molecular weight (Da), percentages of hydrophobic and hydrophilic residues, and theoretical isoelectric point (pI).

Supplementary Table S5. Bioinformatic characteristics of reviewed protein isoforms (CD160/BTLA–HVEM/LIGHT).

Protein	Isoform	Gene	Length (aa)	Molecular weight (Da)	Hydrophobic residues (%)	Hydrophilic residues (%)	Theoretical isoelectric point (pI)
CD160	O95971-2	*CD160*	72	7,801	59.72	40.28	5.29
	O95971-3	*CD160*	234	25,995	51.71	48.29	7.76
	O95971-4	*CD160*	125	13,986	53.60	46.40	8.58
BTLA	Q7Z6A9-2	*BTLA*	241	27,317	45.64	54.36	5.34
LIGHT	O43557-2	*TNFSF14*	204	22,395	52.45	47.55	8.18

Supplementary Table S6. Bioinformatic characteristics of predicted protein isoforms (CD160/BTLA–HVEM/LIGHT).

Protein	Isoform	Gene	Length (aa)	Molecular weight (Da)	Hydrophobic residues (%)	Hydrophilic residues (%)	Theoretical isoelectric point (pI)
CD160	A0ACI8R409	CD160	179	19,580	54.19	45.81	6.84
	A0ACI8UV32	CD160	135	14,650	55.56	44.44	6.8
	A0ACI8RPV6	CD160	26	2,641	80.77	19.23	4.19
HVEM	A0ACI8PNH0	TNFRSF14	281	30,187	55.87	44.13	6.13
	F6UPZ7	TNFRSF14	183	19,553	55.74	44.26	5.33
	A0ACI8QYH6	TNFRSF14	371	39,972	57.41	42.59	6.94
	A0A0D9SF52	TNFRSF14	25	2,909	20.83	79.17	7.47
	A0ACI8RFF3	TNFRSF14	262	28,200	41.22	58.78	6.54
	B9A034	TNFRSF14	193	20,753	37.82	62.18	5.53
	A0ACI8RVS6	TNFRSF14	290	31,131	42.07	57.93	6.32
	A0ACI8QCD6	TNFRSF14	356	37,911	39.90	60.10	6.86
	F6Q0M4	TNFRSF14	231	24,853	37.05	62.95	6.78
	A0ACI8R3R6	TNFRSF14	314	33,921	43.52	56.48	7.3

	A0ACI8VNY9	TNFRSF14	266	28,482	41.83	58.17	6.49
	A0ACI8VP62	TNFRSF14	269	28,794	41.64	58.36	6.49
	A0ACI8VP67	TNFRSF14	277	29,766	40.43	59.57	6.32
LIGHT	A0ABB0MVK2	*TNFSF14*	128	13,982	57.03	42.97	10.05

Supplementary Material S4

CD226/TIGIT/CD96/CD112R–CD155/CD112 immune checkpoint axis

Supplementary Tables S7 and S8 present bioinformatic characteristics of reviewed TIGIT, CD96, CD155, and CD112 isoforms and predicted CD226, TIGIT, CD96, CD112R, CD155, and CD112 isoforms, respectively. The reported data include isoform identifiers, gene symbols, length (aa), molecular weight (Da), percentages of hydrophobic and hydrophilic residues, and theoretical isoelectric point (pI).

Supplementary Table S7. Bioinformatic characteristics of reviewed protein isoforms (CD226/TIGIT/CD96/CD112R–CD155/CD112).

Protein	Isoform	Gene	Length (aa)	Molecular weight (Da)	Hydrophobic residues (%)	Hydrophilic residues (%)	Theoretical isoelectric point (pI)
TIGIT	Q495A1-2	*TIGIT*	170	18,432	61.76	38.24	5.48
CD96	P40200-2	*CD96*	569	63,888	49.74	50.26	6.33
CD155	P15151-2	*PVR*	372	40,125	55.65	44.35	5.43
	P15151-3	*PVR*	364	39,304	57.22	42.78	5.59
	P15151-4	*PVR*	392	42,864	58.93	41.07	6.05
CD112	Q92692-2	*NECTIN2*	479	51,359	57.83	42.17	4.91

Supplementary Table S8. Bioinformatic characteristics of predicted protein isoforms (CD226/TIGIT/CD96/CD112R–CD155/CD112).

Protein	Isoform	Gene	Length (aa)	Molecular weight (Da)	Hydrophobic residues (%)	Hydrophilic residues (%)	Theoretical isoelectric point (pI)
CD226	J3QQW1	*CD226*	276	31,254	52.17	47.83	5.6
	J3QR77	*CD226*	181	21,056	44.75	55.25	9.57
	J3QRQ4	*CD226*	132	15,187	50.76	49.24	4.71
	J3QL19	*CD226*	127	14,638	51.18	48.82	5.04
	J3QKM7	*CD226*	37	4,006	52.78	47.22	4.03

TIGIT	A0A0C4DGA4	*TIGIT*	311	33,926	60.13	39.87	6.11
	C9J0B0	*TIGIT*	169	18,333	55.62	44.38	5.66
	C9JZW6	*TIGIT*	163	17,616	56.44	43.56	6.22
	A0ACI8QDI9	*TIGIT*	134	14,425	61.94	38.06	6.93
	A0ACI8RPP9	*TIGIT*	133	14,296	62.41	37.59	6.47
	A0ACI8SDY8	*TIGIT*	243	26,191	58.44	41.56	5.18
CD96	E9PEJ1	*CD96*	568	63,849	49.82	50.18	6.33
	H7C4F0	*CD96*	146	16,209	46.90	53.10	7.4
	A0ACI8QMK2	*CD96*	537	60,722	50.47	49.53	6.77
	A0ACI8RTR3	*CD96*	481	54,016	50.73	49.27	6.25
	U3KPT0	*CD96*	18	2,364	66.67	33.33	8.62
	A0ACI8PFD4	*CD96*	582	65,320	50.17	49.83	6.09
	A0ACI8QB21	*CD96*	517	58,558	50.87	49.13	6.24
	F8WDV3	*CD96*	118	13,675	53.39	46.61	7.34
	A0ACI8Q221	*CD96*	505	56,777	49.70	50.30	6.05
	A0ACI8QVQ2	*CD96*	530	59,363	49.25	50.75	6.46
	F6RDM0	*CD96*	402	45,621	49.00	51.00	5.94
CD112R	A0A8V8TN58	*PVRIG*	306	32,271	62.42	37.58	8.93
	A0ACI8U3M8	*PVRIG*	211	22,674	63.51	36.49	11.15
	A0ACI8QTQ0	*PVRIG*	189	20,026	65.08	34.92	11.33
CD155	A0A0C4DG49	*PVR*	417	45,321	57.55	42.45	5.79
	A0A9L9PX39	*PVR*	439	47,915	59.91	40.09	6.83
	A0A0A0MSA9	*PVR*	392	42,882	58.93	41.07	6.05
	A0A9L9PXN8	*PVR*	285	31,038	57.19	42.81	5.36
CD112	K7EKE8	*NECTIN2*	210	22,754	51.20	48.80	4.68
	K7EK87	*NECTIN2*	56	6,066	50.0	50.0	4.93
	A0ACI8Q388	*NECTIN2*	567	60,870	56.26	43.74	4.76
	A0ACI8Q830	*NECTIN2*	585	62,950	55.90	44.10	4.87
	A0ACI8R3H6	*NECTIN2*	408	43,713	55.39	44.61	4.5
	A0ACI8UG70	*NECTIN2*	544	58,377	56.25	43.75	4.63
	A0ACI8QUB8	*NECTIN2*	562	60,277	56.23	43.77	4.7
	K7ERL3	*NECTIN2*	54	6,180	56.60	43.40	4.45
	A0ACI8Q390	*NECTIN2*	541	58,056	55.82	44.18	4.64
	A0ACI8SWJ0	*NECTIN2*	537	57,685	55.87	44.13	4.66
	A0ACI8SWM8	*NECTIN2*	546	58,521	55.86	44.14	4.75
	A0ACI8S907	*NECTIN2*	439	47,071	57.18	42.82	4.47

Supplementary Material S5

TIM-3/Galectin-9/CEACAM1 immune checkpoint axis

Supplementary Tables S9 and S10 present bioinformatic characteristics of reviewed and predicted isoforms of TIM-3, Galectin-9, and CEACAM1, respectively. The reported data include isoform identifiers, gene symbols, length (aa), molecular weight (Da), percentages of hydrophobic and hydrophilic residues, and theoretical isoelectric point (pI).

Supplementary Table S9. Bioinformatic characteristics of reviewed protein isoforms (TIM-3/Galectin-9/CEACAM1).

Protein	Isoform	Gene	Length (aa)	Molecular weight (Da)	Hydrophobic residues (%)	Hydrophilic residues (%)	Theoretical isoelectric point (pI)
TIM-3	Q8TDQ0-2	*HAVCR2*	142	16,149	62.68	37.32	4.99
Galectin-9	O00182-2	*LGALS9*	323	35,888	57.89	42.11	7.41
	O00182-3	*LGALS9*	311	34,691	56.59	43.41	7.41
	O00182-4	*LGALS9*	246	26,777	60.98	39.02	7.96
	O00182-5	*LGALS9*	234	25,579	59.40	40.60	7.96
	O00182-6	*LGALS9*	343	38,321	56.56	43.44	8.68
CEACAM1	P13688-2	*CEACAM1*	417	45,950	49.40	50.60	5.58
	P13688-3	*CEACAM1*	321	35,302	51.09	48.91	5.21
	P13688-4	*CEACAM1*	351	38,578	51.57	48.43	4.92
	P13688-5	*CEACAM1*	461	50,349	50.54	49.46	5.07
	P13688-6	*CEACAM1*	430	46,910	50.93	49.07	5.12
	P13688-7	*CEACAM1*	252	27,431	53.97	46.03	5.61
	P13688-8	*CEACAM1*	464	50,521	52.37	47.63	5.53
	P13688-9	*CEACAM1*	399	43,062	53.88	46.12	5.5
	P13688-10	*CEACAM1*	468	51,147	51.92	48.08	5.9
	P13688-11	*CEACAM1*	368	39,871	54.35	45.65	5.08

Supplementary Table S10. Bioinformatic characteristics of predicted protein isoforms (TIM-3/Galectin-9/CEACAM1).

Protein	Isoform	Gene	Length (aa)	Molecular weight (Da)	Hydrophobic residues (%)	Hydrophilic residues (%)	Theoretical isoelectric point (pI)
TIM-3	A0A8V8TMM7	*HAVCR2*	60	6,636	50.00	50.00	9.23
	A0A8Q3SJ15	*HAVCR2*	138	15,545	62.32	37.68	5.43
	A0A8Q3SJ74	*HAVCR2*	300	33,323	59.00	41.00	5.39
	A0A8Q3SIX1	*HAVCR2*	241	26,632	60.17	39.83	6.34
	E5RFR4	*HAVCR2*	184	20,272	55.98	44.02	5.95
	E5RHN3	*HAVCR2*	273	30,331	59.71	40.29	4.84
Galectin-9	J3KS82	*LGALS9*	175	19,131	58.86	41.14	7.49
	J3KSA0	*LGALS9*	137	15,175	61.76	38.24	8.31
	A0ACI8PNA0	*LGALS9*	297	33,123	55.22	44.78	7.4
	A0ACI8Q2U5	*LGALS9*	332	36,921	58.13	41.87	9.06
	A0ACI8QYU0	*LGALS9*	361	40,237	57.89	42.11	8.87
	A0ACI8RW51	*LGALS9*	329	36,607	58.05	41.95	7.68
	A0ACI8QBQ6	*LGALS9*	326	36,507	57.06	42.94	8.68
	A0ACI8QL53	*LGALS9*	317	35,409	56.78	43.22	7.68
	A0ACI8R050	*LGALS9*	329	36,528	58.06	41.94	7.41
	A0ACI8R7A5	*LGALS9*	300	33,291	58.33	41.67	7.94
	K7EPS0	*LGALS9*	115	12,895	60.87	39.13	10.61
	A0ACI8UIJ2	*LGALS9*	333	36,428	56.46	43.54	6.32
	J3QKK6	*LGALS9*	75	7,899	54.67	45.33	5.32
	J3QS92	*LGALS9*	125	13,839	58.87	41.13	9.91
CEACAM1	A0A7I2V3A7	*CEACAM1*	54	5,473	77.36	22.64	7.52
	A0ACI8TCL1	*CEACAM1*	345	37,780	51.30	48.70	5.11
	A0ACI8PK93	*CEACAM1*	252	27,471	53.57	46.43	5.69
	M0R109	*CEACAM1*	99	10,969	54.55	45.45	5.02
	A0ACI8RC08	*CEACAM1*	461	50,102	50.76	49.24	5.43
	A0ACI8S157	*CEACAM1*	465	51,255	47.96	52.04	5.22
	M0R2K4	*CEACAM1*	94	10,399	55.32	44.68	4.79

Supplementary Material S6

LAG-3/FGL1 immune checkpoint axis

Supplementary Table S11 presents bioinformatic characteristics of a reviewed LAG-3 isoform, whereas Supplementary Table S12 covers predicted LAG-3 and FGL1 isoforms. The reported data include isoform identifiers, gene symbols, length (aa), molecular weight (Da), percentages of hydrophobic and hydrophilic residues, and theoretical isoelectric point (pI).

Supplementary Table S11. Bioinformatic characteristics of reviewed protein isoforms (LAG-3/FGL1).

Protein	Isoform	Gene	Length (aa)	Molecular weight (Da)	Hydrophobic residues (%)	Hydrophilic residues (%)	Theoretical isoelectric point (pI)
LAG-3	P18627-2	*LAG3*	360	39,103	61.39	38.61	9.76

Supplementary Table S12. Bioinformatic characteristics of predicted protein isoforms (LAG-3/FGL1).

Protein	Isoform	Gene	Length (aa)	Molecular weight (Da)	Hydrophobic residues (%)	Hydrophilic residues (%)	Theoretical isoelectric point (pI)
LAG-3	A0ACI8P9V4	*LAG3*	355	38,892	58.59	41.41	5.43
	A0ACI8QKE4	*LAG3*	444	48,576	61.26	38.74	7.82
	A0ACI8RDQ2	*LAG3*	508	55,391	60.63	39.37	6.73
FGL1	A0ACI8SEY3	*FGL1*	309	36,074	49.51	50.49	5.51
	A0ACI8QZ19	*FGL1*	311	36,250	49.52	50.48	5.5
	A0ACI8RRJ1	*FGL1*	307	35,647	49.51	50.49	5.39
	A0ACI8RZ70	*FGL1*	226	26,470	49.12	50.88	5.41
	A0ACI8QZ35	*FGL1*	309	35,962	49.51	50.49	5.29
	A0ACI8REB8	*FGL1*	287	33,412	49.83	50.17	5.25
	A0ACI8S3D1	*FGL1*	325	37,878	50.46	49.54	5.4
	A0ACI8S0S9	*FGL1*	308	35,776	49.35	50.65	5.29
	A0ACI8S7T2	*FGL1*	302	35,217	49.34	50.66	5.62
	A0ACI8SGF8	*FGL1*	300	35,001	49.0	51.0	5.23

Supplementary Material S7

VISTA/PSGL-1 immune checkpoint axis

Supplementary Table S13 presents bioinformatic characteristics of a reviewed PSGL-1 isoform, whereas Supplementary Table S14 covers predicted VISTA and PSGL-1 isoforms. The reported data include isoform identifiers, gene symbols, length (aa), molecular weight (Da), percentages of hydrophobic and hydrophilic residues, and theoretical isoelectric point (pI).

Supplementary Table S13. Bioinformatic characteristics of reviewed protein isoforms (VISTA/PSGL-1).

Protein	Isoform	Gene	Length (aa)	Molecular weight (Da)	Hydrophobic residues (%)	Hydrophilic residues (%)	Theoretical isoelectric point (pI)
PSGL-1	Q14242-2	*SELPLG*	428	44,648	53.97	46.03	4.26

Supplementary Table S14. Bioinformatic characteristics of predicted protein isoforms (VISTA/PSGL-1).

Protein	Isoform	Gene	Length (aa)	Molecular weight (Da)	Hydrophobic residues (%)	Hydrophilic residues (%)	Theoretical isoelectric point (pI)
VISTA	A0ACI8S0D4	*VSIR*	245	26,402	55.92	44.08	5.76
	A0ACI8UY50	*VSIR*	312	33,979	55.77	44.23	6.13
	A0ACI8PTX9	*VSIR*	292	31,957	56.51	43.49	6.26
	A0ACI8QJH8	*VSIR*	381	42,089	56.17	43.83	7.43
	A0ACI8RLB2	*VSIR*	168	17,819	61.31	38.69	4.98
	A0ACI8R6M8	*VSIR*	149	15,868	63.76	36.24	5.15
	A0ACI8RAX3	*VSIR*	113	12,033	61.95	38.05	4.54
	A0ACI8UXT4	*VSIR*	323	35,143	56.35	43.65	6.13
PSGL-1	A0A0C4DFY0	*SELPLG*	402	42,148	53.48	46.52	4.28

Supplementary Material S8

OX40/OX40L immune checkpoint axis

Supplementary Table S15 presents bioinformatic characteristics of a reviewed OX40L isoform, whereas Supplementary Table S16 covers predicted OX40 and OX40L isoforms. The reported data include isoform identifiers, gene symbols, length (aa), molecular weight (Da), percentages of hydrophobic and hydrophilic residues, and theoretical isoelectric point (pI).

Supplementary Table S15. Bioinformatic characteristics of reviewed protein isoforms (OX40/OX40L).

Protein	Isoform	Gene	Length (aa)	Molecular weight (Da)	Hydrophobic residues (%)	Hydrophilic residues (%)	Theoretical isoelectric point (pI)
OX40L	P23510-2	*TNFSF4*	133	15,427	42.86	57.14	6.17

Supplementary Table S16. Bioinformatic characteristics of predicted protein isoforms (OX40/OX40L).

Protein	Isoform	Gene	Length (aa)	Molecular weight (Da)	Hydrophobic residues (%)	Hydrophilic residues (%)	Theoretical isoelectric point (pI)
OX40	A0A8V8TQH5	*TNFRSF4*	303	32,276	56.77	43.23	7.39
	A0A8V8TQH9	*TNFRSF4*	325	34,458	60.92	39.08	8.61
	A0A8V8TQV3	*TNFRSF4*	335	34,951	61.79	38.21	8.95
	A0A8V8TQV7	*TNFRSF4*	253	26,615	62.06	37.94	7.7
	A0A8V8TPH3	*TNFRSF4*	203	21,705	60.10	39.90	7.55
	A0A8V8TPH7	*TNFRSF4*	311	32,366	62.70	37.30	9.19
	A0A8V8TPN6	*TNFRSF4*	298	31,525	58.72	41.28	7.43
	A0A8V8TPP1	*TNFRSF4*	189	20,074	58.73	41.27	7.93
	A0A8V8TP52	*TNFRSF4*	355	37,070	60.0	40.0	7.38
	A0A8V8TP56	*TNFRSF4*	234	24,836	52.99	47.01	7.09
OX40L	A0AAQ5BI34	*TNFSF4*	93	10,548	54.84	45.16	6.54

Supplementary Material S9

ICOS/ICOSL immune checkpoint axis

Supplementary Table S17 presents bioinformatic characteristics of reviewed ICOSL isoforms, whereas Supplementary Table S18 covers predicted ICOS and ICOSL isoforms. The reported data include isoform identifiers, gene symbols, length (aa), molecular weight (Da), percentages of hydrophobic and hydrophilic residues, and theoretical isoelectric point (pI).

Supplementary Table S17. Bioinformatic characteristics of reviewed protein isoforms (ICOS/ICOSL).

Protein	Isoform	Gene	Length (aa)	Molecular weight (Da)	Hydrophobic residues (%)	Hydrophilic residues (%)	Theoretical isoelectric point (pI)
ICOSL	O75144-2	*ICOSLG*	309	34,225	48.33	51.67	4.87
	O75144-3	*ICOSLG*	185	20,120	55.14	44.86	5.33

Supplementary Table S18. Bioinformatic characteristics of predicted protein isoforms (ICOS/ICOSL)

Protein	Isoform	Gene	Length (aa)	Molecular weight (Da)	Hydrophobic residues (%)	Hydrophilic residues (%)	Theoretical isoelectric point (pI)
ICOS	A0ACI8QMD0	*ICOS*	87	10,021	60.92	39.08	8.24
ICOSL	A0A2R8Y5W7	*ICOSLG*	361	39,638	48.38	51.62	4.97
	K4DIA0	*ICOSLG*	473	51,925	54.76	45.24	7.19
	A0A8V8TQ42	*ICOSLG*	261	28,743	49.64	50.36	5.35
	A0A8V8TQ73	*ICOSLG*	240	26,598	50.42	49.58	5.2
	A0A8V8TQV9	*ICOSLG*	304	33,481	54.28	45.72	4.94
	A0A8V8TQW2	*ICOSLG*	214	23,425	53.74	46.26	4.86
	A0A096LPE5	*ICOSLG*	77	8,167	66.23	33.77	11.01
	A0ACI8QH76	*ICOSLG*	88	9,453	61.36	38.64	5.8
	A0A8V8TPM3	*ICOSLG*	217	23,718	53.92	46.08	4.97
	A0ACI8R2T6	*ICOSLG*	314	34,750	52.23	47.77	5.02
	A0A8V8TR62	*ICOSLG*	35	3,821	60.0	40.0	10.96

Supplementary Material S10

4-1BB/4-1BBL immune checkpoint axis

Supplementary Table S19 presents bioinformatic characteristics of predicted 4-1BB protein isoforms. The reported data include isoform identifiers, gene symbols, length (aa), molecular weight (Da), percentages of hydrophobic and hydrophilic residues, and theoretical isoelectric point (pI).

Supplementary Table S19. Bioinformatic characteristics of predicted protein isoforms (4-1BB/4-1BBL).

Protein	Isoform	Gene	Length (aa)	Molecular weight (Da)	Hydrophobic residues (%)	Hydrophilic residues (%)	Theoretical isoelectric point (pI)
4-1BB	K7EJ11	*TNFRSF9*	75	8,402	44.0	56.0	5.34
	K7EJQ2	*TNFRSF9*	87	9,435	57.47	42.53	8.89
	A0A6I8PRR4	*TNFRSF9*	115	12,493	48.70	51.30	6.73

Supplementary Material S11

GITR/GITRL immune checkpoint axis

Supplementary Table S20 presents bioinformatic characteristics of a predicted GITR protein isoform. The reported data include isoform identifiers, gene symbols, length (aa), molecular weight (Da), percentages of hydrophobic and hydrophilic residues, and theoretical isoelectric point (pI).

Supplementary Table S20. Bioinformatic characteristics of predicted protein isoforms (GITR/GITRL).

Protein	Isoform	Gene	Length (aa)	Molecular weight (Da)	Hydrophobic residues (%)	Hydrophilic residues (%)	Theoretical isoelectric point (pI)
GITR	J3KT02	*TNFRSF18*	169	18,485	59.17	40.83	5.34

Supplementary Material S12

CD27/CD70 immune checkpoint axis

Supplementary Table S21 presents bioinformatic characteristics of a reviewed CD70 isoform, whereas Supplementary Table S22 covers predicted CD27 and CD70 isoforms. The reported data include isoform identifiers, gene symbols, length (aa), molecular weight (Da), percentages of hydrophobic and hydrophilic residues, and theoretical isoelectric point (pI).

Supplementary Table S21. Bioinformatic characteristics of reviewed protein isoforms (CD27/CD70).

Protein	Isoform	Gene	Length (aa)	Molecular weight (Da)	Hydrophobic residues (%)	Hydrophilic residues (%)	Theoretical isoelectric point (pI)
CD70	P32970-2	*CD70*	208	23,438	57.21	42.79	8.02

Supplementary Table S22. Bioinformatic characteristics of predicted protein isoforms (CD27/CD70).

Protein	Isoform	Gene	Length (aa)	Molecular weight (Da)	Hydrophobic residues (%)	Hydrophilic residues (%)	Theoretical isoelectric point (pI)
CD27	A0ACI8QCE2	*CD27*	265	29,608	52.45	47.55	6.77
	A0ACI8R6I0	*CD27*	291	32,631	52.92	47.08	7.16
	A0ACI8RPH1	*CD27*	251	28,163	52.99	47.01	6.79
CD70	M0QZW2	CD70	65	7,104	64.62	35.38	6.32

Supplementary Material S13

CD40/CD40L immune checkpoint axis

Supplementary Table S23 presents bioinformatic characteristics of predicted CD40 and CD40L protein isoforms. The reported data include isoform identifiers, gene symbols, length (aa), molecular weight (Da), percentages of hydrophobic and hydrophilic residues, and theoretical isoelectric point (pI).

Supplementary Table S23. Bioinformatic characteristics of predicted protein isoforms (CD40/CD40L).

Protein	Isoform	Gene	Length (aa)	Molecular weight (Da)	Hydrophobic residues (%)	Hydrophilic residues (%)	Theoretical isoelectric point (pI)
CD40	A0A087X1D0	*CD40*	151	17,035	50.33	49.67	5.22
	H0YE23	*CD40*	159	17,914	50.0	50.0	5.93
	A0A8Q3SI60	*CD40*	237	25,769	51.05	48.95	4.98
	A0A8Q3WKP3	*CD40*	191	21,046	50.79	49.21	4.87
	A0A8Q3WKP7	*CD40*	48	5,370	64.58	35.42	4.03
	A0ACI8S9Q5	*CD40*	279	30,838	50.90	49.10	5.07
	A0ACI8UDT8	*CD40*	282	31,129	52.13	47.87	5.3
	A0ACI8RPH7	*CD40*	249	27,506	51.81	48.19	5.21

	A0ACI8RZ84	*CD40*	261	28,852	49.81	50.19	5.21
	A0ACI8SDV9	*CD40*	281	31,010	50.89	49.11	5.07
	A0ACI8TXF4	*CD40*	229	25,490	50.66	49.34	5.21
	A0ACI8TXI2	*CD40*	243	27,270	51.44	48.56	6.07
	A0ACI8RT92	*CD40*	275	30,491	51.27	48.73	5.39
	A0ACI8QD64	*CD40*	225	25,099	51.56	48.44	5.51
	A0ACI8QVG6	*CD40*	275	30,447	51.64	48.36	5.30
	A0ACI8RM31	*CD40*	250	27,739	51.60	48.40	5.52
CD40L	F6X5E6	*CD40LG*	240	27,150	48.33	51.67	7.8
	A0A8Q3WKP2	*CD40LG*	107	12,518	49.53	50.47	8.39
	A0A8Q3WL88	*CD40LG*	63	7,148	52.38	47.62	9.21

Supplementary Material S14

CD2/CD58 immune checkpoint axis

Supplementary Table S24 presents bioinformatic characteristics of reviewed CD58 isoforms, whereas Supplementary Table S25 covers predicted CD2 and CD58 isoforms. The reported data include isoform identifiers, gene symbols, length (aa), molecular weight (Da), percentages of hydrophobic and hydrophilic residues, and theoretical isoelectric point (pI).

Supplementary Table S24. Bioinformatic characteristics of reviewed protein isoforms (CD2/CD58).

Protein	Isoform	Gene	Length (aa)	Molecular weight (Da)	Hydrophobic residues (%)	Hydrophilic residues (%)	Theoretical isoelectric point (pI)
CD58	P19256-2	*CD58*	237	26,661	50.21	49.79	5.59
	P19256-3	*CD58*	248	27,960	49.19	50.81	6.22

Supplementary Table S25. Bioinformatic characteristics of predicted protein isoforms (CD2/CD58).

Protein	Isoform	Gene	Length (aa)	Molecular weight (Da)	Hydrophobic residues (%)	Hydrophilic residues (%)	Theoretical isoelectric point (pI)
CD2	Q5JVN7	*CD2*	133	15,327	33.3	66.7	5.98
CD58	B1AMW1	*CD58*	240	27,018	50.42	49.58	5.59
	A0ACI8S596	*CD58*	208	23,556	50.0	50.0	5.98
	H0YDI1	*CD58*	131	15,136	45.80	54.20	6.7

Supplementary Material S15

B7-H3/B7-H4 immune checkpoint axis

Supplementary Tables S26 and S27 present bioinformatic characteristics of reviewed and predicted isoforms of B7-H3 and B7-H4, respectively. The reported data include isoform identifiers, gene symbols, length (aa), molecular weight (Da), percentages of hydrophobic and hydrophilic residues, and theoretical isoelectric point (pI).

Supplementary Table S26. Bioinformatic characteristics of reviewed protein isoforms (B7-H3 and B7-H4).

Protein	Isoform	Gene	Length (aa)	Molecular weight (Da)	Hydrophobic residues (%)	Hydrophilic residues (%)	Theoretical isoelectric point (pI)
B7-H3	Q5ZPR3-2	*CD276*	316	33,791	61.49	38.51	4.62
	Q5ZPR3-3	*CD276*	493	52,761	57.20	42.80	5.11
	Q5ZPR3-4	*CD276*	533	57,165	56.29	43.71	4.77
B7-H4	Q7Z7D3-2	*VTCN1*	166	18,275	57.83	42.17	4.99
	Q7Z7D3-3	*VTCN1*	87	9,875	64.37	35.63	6.72
	Q7Z7D3-4	*VTCN1*	187	20,743	52.41	47.59	7.44

Supplementary Table S27. Bioinformatic characteristics of predicted B7-H3 and B7-H4 protein isoforms.

Protein	Isoform	Gene	Length (aa)	Molecular weight (Da)	Hydrophobic residues (%)	Hydrophilic residues (%)	Theoretical isoelectric point (pI)
B7-H3	A0A0C4DGH0	*CD276*	388	41,768	54.12	45.88	4.53
	A0ACI8RJC3	*CD276*	489	52,291	55.01	44.99	4.64
	H0YKQ9	*CD276*	51	5,226	72.55	27.45	5.28
	H0YN29	*CD276*	170	18,304	53.85	46.15	4.38
	H0YN85	*CD276*	136	14,456	61.76	38.24	5.04
B7-H4	A0ACI8RSK8	*VTCN1*	73	7,977	68.49	31.51	9.07
	Q5T2L0	*VTCN1*	285	31,259	54.39	45.61	5.56
